# MXene-based electrochemical devices applied for healthcare applications

**DOI:** 10.1007/s00604-023-06163-6

**Published:** 2024-01-11

**Authors:** Lenka Lorencova, Peter Kasak, Natalia Kosutova, Monika Jerigova, Eva Noskovicova, Alica Vikartovska, Marek Barath, Pavol Farkas, Jan Tkac

**Affiliations:** 1 Institute of Chemistry, Slovak Academy of Sciences, Dubravska cesta 5807/9, 845 38 Bratislava, Slovak Republic; 2 Center for Advanced Materials, Qatar University, P.O. Box 2713, Doha, Qatar; 3 International Laser Center, Slovak Center of Scientific and Technical Information, Ilkovicova 3, 841 04 Bratislava, Slovak Republic; 4 Department of Physical and Theoretical Chemistry, Faculty of Natural Sciences, Comenius University, Ilkovicova 6, Mlynska Dolina, 842 15 Bratislava, Slovak Republic

**Keywords:** MXene, Electrochemical (bio)sensors, Wearable electronics, Human activity monitoring

## Abstract

The initial part of the review provides an extensive overview about MXenes as novel and exciting 2D nanomaterials describing their basic physico-chemical features, methods of their synthesis, and possible interfacial modifications and techniques, which could be applied to the characterization of MXenes. Unique physico-chemical parameters of MXenes make them attractive for many practical applications, which are shortly discussed. Use of MXenes for healthcare applications is a hot scientific discipline which is discussed in detail. The article focuses on determination of low molecular weight analytes (metabolites), high molecular weight analytes (DNA/RNA and proteins), or even cells, exosomes, and viruses detected using electrochemical sensors and biosensors. Separate chapters are provided to show the potential of MXene-based devices for determination of cancer biomarkers and as wearable sensors and biosensors for monitoring of a wide range of human activities.

**Graphical Abstract:**

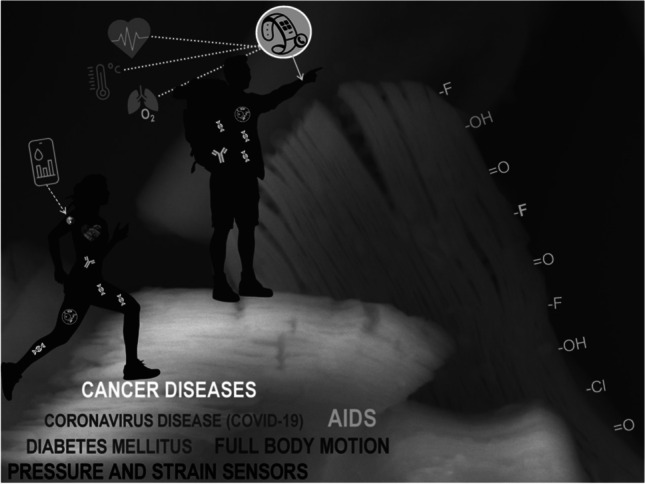

## Introduction

Many solution-processed two-dimensional (2D) materials were quite small in flake size owing to low mechanical strength leading to the fracture of 2D sheets during delamination [1]. A number of early day 2D materials were also hydrophobic [2] and unstable, when exposed to air [3–5]. Hence, the discovery of a family of 2D carbides and nitrides with metallic conductivity, hydrophilicity, ease of processing, relatively high yields, and large size flakes had a profound effect on the entire field of material science.

Ever since then, the realm of 2D materials [6] became much larger and a very dynamic and exciting research field. The fact that MXenes emerged early meant that they attracted significant attention to the field of 2D nanomaterials besides graphene. Soon thereafter, 2D nanomaterials made of Si, Ge, Sn, and several other elements with weakly bonded layered precursors were demonstrated [7]. The main initial practical applications of 2D nanomaterials were in microelectronics [8–10].

**Fig. 1:**
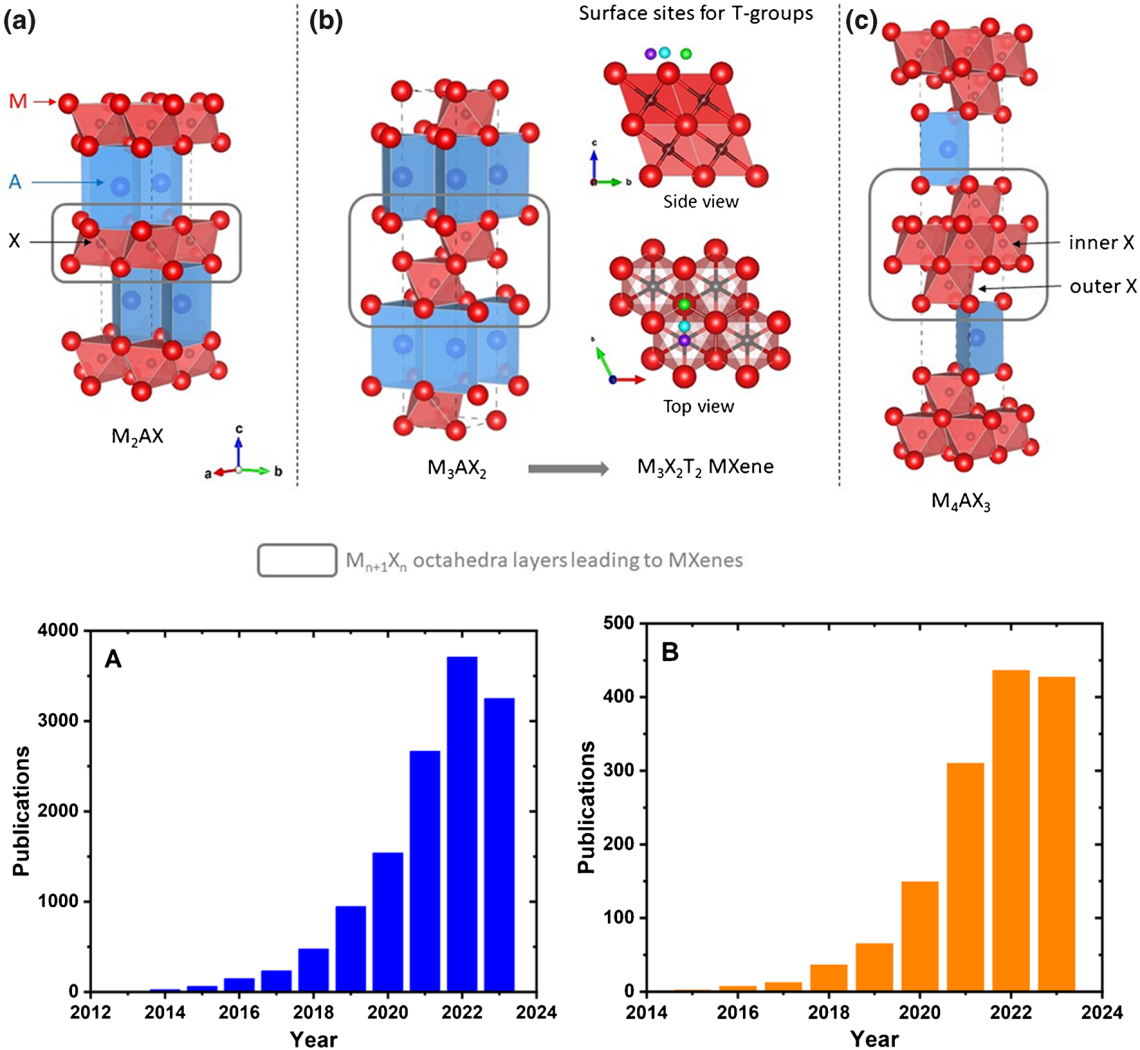
Crystallographic structures of MAX phases with *n* = 1 [**a**], 2 [**b**], and 3 [**c**] octahedral layers (highlighted in gray) between the A element layers (in blue). The octahedral layers forming the skeleton of the corresponding MXene are circled in gray. The M element is represented as red spheres and the X element as black ones. In [**b**], the three different sites considered for the T-groups on the MXenes’ surface are given: FCC (green), HCP (purple), and bridge (cyan). In order to ease their identification, only one surface group is sketched in these structural models, but all calculations were performed on fully functionalized surfaces, i.e., corresponding to M_*n*+1_X_*n*_T_2_ compositions (with *T* =  − O, − OH, − F, or − Cl); see the SI, part S2. One should notice that the Mo_2_Ga_2_C structure is different from those of the MAX phases with a double A element layer between the octahedral layers. Structural models were drawn with VESTA software [13]. Reproduced with permission from ref. [14]. Copyright 2023 American Chemical Society (upper image). Publication dynamics expressed as number of publications published for the term “MXene” (A) and a combination of the terms “MXene AND (healthcare OR medicinal OR medical OR biomedicinal OR biomedicine OR medicine)” (B). The search was performed using the Web of Science database (lower image)

Early transition metal carbides and nitrides were characterized by their high metallic electrical conductivity, hardness, and excellent chemical stability and they were used for decades as bulk ceramic materials mostly for high-temperature applications and as cutting tools. Reducing the dimensionality of metal carbides and nitrides turned out to be a daunting task mainly due to strong bond between the transition metal and carbon/nitrogen atoms (mostly covalent/metallic bonds). In 2011, it was showed that by simple immersing of Ti_3_AlC_2_ in hydrofluoric acid (HF) at room temperature, one could selectively etch the Al layers leaving behind a 2D nanomaterial made of titanium carbide (Ti_3_C_2_) for the first time [6]. At some point, it became clear that the synthesis of 2D nanomaterials does not necessarily require van der Waals bonded layered precursors and hence a number of new materials have been discovered including different types of MXenes (Fig. 1, upper image) [11]. In fact, Ti_3_C_2_ was the first MXene reported in 2011 [6] and shortly after the synthesis of other MXenes, e.g., Ti_2_C and Ta_4_C_3_, from their MAX phase precursors, demonstrating three types of possible structures (M_2_X, M_3_X_2_, and M_4_X_3_). The MAX phases are layered hexagonal (P_63_/mmc space group) materials and can be described as transition metal carbide/nitride sheets of octahedral blocks, where the *X* atoms are in the centers of the octahedrons, glued together with pure *A* layers. Back in 2011, there were approximately 70 MAX phases known; today, their number exceeds 150, with new ones discovered on a routine basis, proving a large number of precursors for MXene synthesis. Currently, more than 40 MXene compositions exist with the ultimate number being far greater [12].

The field of MXene-based applications is a very active scientific field, what can be documented by the number of publications published in the last 11 years since the first publication in 2011 (Fig. 1A, lower image). Application of MXene in healthcare is slightly lagged behind since the first publication was published in 2015, but since then the field is very dynamic (Fig. 1B, lower image). Thus, in this review paper, our aim was to provide overview of the advancements achieved by using MXene for the healthcare applications.

**Fig. 2:**
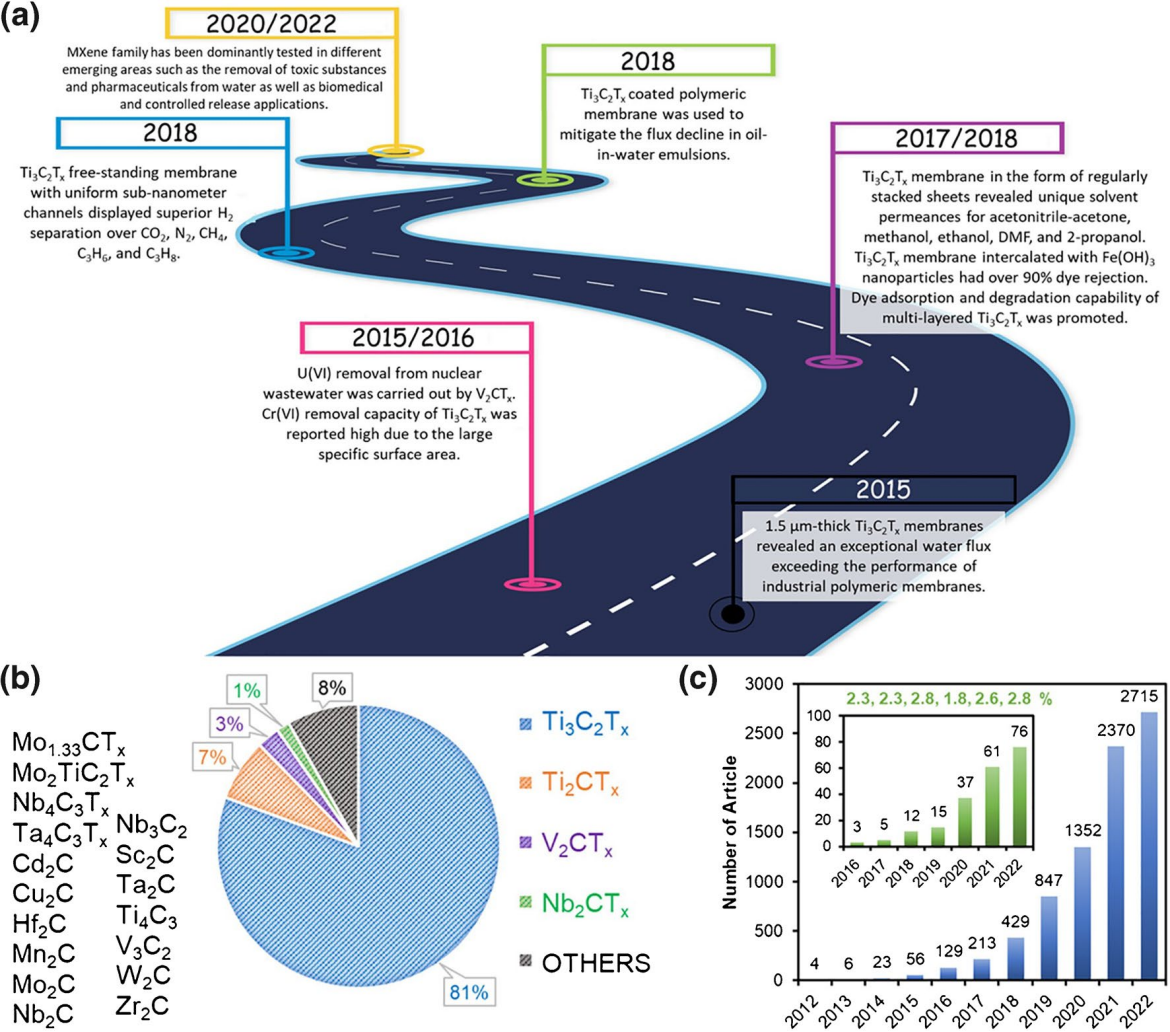
Literature survey of MXene nanomaterials. **a** Milestones of each separation application [18–36], **b** distribution of MXene types that were investigated for the separation application (other MXene types were listed on the left side of the figure), and **c** growth of the MXene-based studies in all fields on the Web of Science database reported on December of 2022 (reviews and patents were excluded). Inset figure of panel **c** represents the growth of the MXene literature in only separation applications, along with its percentage over all fields in each year. Reproduced with permission from ref. [37], which is an open access publication. Copyright 2023 American Chemical Society

A brief literature survey of MXene nanomaterials is shown in Fig. 2 [15]. When the “*A*” atoms of the MAX phase are etched, the freshly exposed and unsaturated transition metal atoms are immediately coordinated by anions present in the etchant, forming the surface terminations *T*_*x*_ with a chemical formula M_*n*+1_C_*n*_T_*x*_ [16]. MXenes are defined by their general structure of M_*n*+1_X_*n*_T_*x*_, where *M* is an early transition metal (Sc, Y, Ti, Zr, Hf, V, Nb, Ta, Cr, Mo, W), *X* is a carbon and/or nitrogen, and *T*_*x*_ stands for surface terminations, such as O, OH, F or Cl, and *n* = 1–4 [17]

MXenes’ electronic properties range from metallic to semimetal, semiconducting, and insulating [16]. MXenes’ unique properties, such as their metal-like electrical conductivity reaching ≈20,000 S cm^−1^, extended surface area make them an appealing choice in applications for energy storage, biomedicine, communications, and environmental applications. On the other hand, such high electrical conductivities combined with the surface terminations allow covalent or electrostatic anchoring of other molecules and nanoparticles to design interfaces with strongly associated (bio)polymers or nanoparticles [17].

Resistivity of Ti_3_C_2_T_*x*_ films (15.8 μΩ⋅m) is within an order of magnitude of resistivity of single flakes (2.3 μΩ m), demonstrating efficient charge transport between the flakes within the thin films. At the same time, Ti_3_C_2_T_*x*_ shows high (~ 2 × 10^21^ cm^−3^) intrinsic charge carrier density and relatively high (~ 34 cm^2^ V^−1^ s^−1^) carrier mobility, while Mo-based MXenes demonstrated lower intrinsic carrier densities (~ 10^20^ cm^−3^ for Mo_2_Ti_2_C_3_T_*x*_ and ~ 10^19^ cm^−3^ for Mo_2_TiC_2_T_*x*_). Ti_3_C_2_T_*x*_ has hence attracted attention as a material for making electronic device contacts, electron emitters, transparent conductor layers in perovskite solar cells, and light-emitting diodes (LED). Further, Ti_3_C_2_T_*x*_ demonstrates negative magnetoresistance, and Mo-containing MXenes typically exhibit positive magnetoresistance [38]. Theory predicts that the bandgap and the magnetic properties could be engineered by adjusting the thin-film chemistry and terminations [39].

MXenes are characterized by high electronic conductivity and wide range of interesting optical absorption properties. These unique properties are the result of quantum confinement effect in the atomically thin 2D layers and are strongly dependent on the layer thickness and composition. The individual titanium oxide nanosheets exhibit large dielectric constant and electronic permittivity making MXenes suitable for applications such as electromagnetic interference (EMI) shielding [40–43], pressure and molecular sensors [44, 45], and transparent conductors [46].

The electronic properties of MXenes such as metal-to-insulator transition, ultralow work function, topological insulator, large electronic anisotropy, and massless Dirac dispersion near the Fermi level have been formerly extensively investigated computationally. Bare MXenes are metallic but some become semiconductors upon surface functionalization. The outer transition metal layers (M′ in M′_2_ M″C_2_T_*x*_ and M′_2_ M″_2_C_3_T_*x*_) in ordered multi-elemental transition metal MXenes play a more important role in electronic properties than the M″ inner core metals. OH- and F-terminations were predicted to have similar effect on MXenes’ electronic structure because they can only receive one electron from the surface metal. OH-termination leads to negative surface dipole moment, and thus decrease in the work function. Hydroxyl-terminated MXenes are expected to have an ultralow work function and thus can be efficient electron emitters that are attractive as field emitter cathodes in field effect transistors. Some MXenes are predicted to be 2D topological insulators with potential applications ranging from basic spintronic devices to quantum computing. Since strong spin–orbit coupling (SOC) is required for topological insulators, MXenes with heavy 4d and 5d transition metals (Mo, W, Zr, and Hf) are suitable candidates [47, 48].

MXenes being van der Waals materials exhibit anisotropy of electronic conductivity in the in-plane and out-of-plane directions. It was shown that the in-plane conductivity is an order of magnitude higher than the out-of-plane conductivity. Moreover, effective mass of electrons and holes in the basal plane were calculated to be quite small (< 0.5 *m*_0_), while that of electrons and holes perpendicular to the layers were estimated to be infinite.

Ti_3_C_2_T_*x*_ shows optical absorption at 0.8 eV and 1.7 eV that were previously attributed to surface plasmons and interband electronic transitions is located below 1.6 eV and above 3 eV. Moreover, Ti_3_C_2_T_*x*_ is 93% transparent at thicknesses of about 4 nm, which makes it a great candidate for transparent electrodes [38].

The optical and plasmonic properties of such nanomaterial are attractive for applications in ultrafast lasers [49, 50], optical communication [51, 52], in surface-enhanced Raman spectroscopy (SERS) [53, 54], as broadband absorbers [55], and in light-to-heat conversion [56, 57]. Ti_3_C_2_T_*x*_ exhibits nonlinear light absorption (saturable absorption); i.e., the transmission increases nonlinearly with increasing illuminating intensity. Additionally, nonlinear absorption coefficients of Ti_3_C_2_T_*x*_ as high as − 10^−21^ m^2^ V^−2^ were measured indicating potential use in optical switching applications and hence metallic Ti_3_C_2_T_*x*_ and Ti_3_CNT_*x*_ were used in femtosecond mode-locked lasers. The nonlinear optical performance of MXenes is comparable, if not superior, to other 2D materials such as transition metal dichalcogenides, graphene, and black phosphorus.

Ti_3_C_2_T_*x*_ exhibits attractive plasmonic properties potentially applicable in SERS applications. Electron energy loss spectroscopy analysis has shown that multi-layered Ti_3_C_2_T_*x*_ has intense surface plasmons with energy range from 0.3 to 1 eV that dominate over bulk plasmons even at 45-nm layer thickness. The bulk plasmon peak is independent of the layer thickness, unlike other 2D materials where the bulk plasmon peak blue shifts when going from few layers to a bulk state [47, 48].

Mechanically, MXenes offer high strength and module of elasticity; Young’s module of single layers can be as high as 330 and 390 MPa for Ti_3_C_2_T_*x*_ and Nb_4_C_3_T_*x*_, respectively—higher than for graphene oxide or MoS_2_. At the same time, these numbers are the highest among all solution-processable materials, which further supports the use of MXenes in composite applications [38]. Furthermore, MXenes provide a combination of conductivity with interesting redox properties [16]. Importantly MXenes show no cytotoxicity, and upon degradation they turn into nontoxic products, such as TiO_2_, CO_2_, or CH_4_.

In order to boost MXenes’ functionality, they can be combined with, e.g., metal nanoparticles, polymers [58–62]. Among the abovementioned behavior, the interactions of MXenes with various electrolytes, offering insight into the obstacles [63] and potential related to their practical application [2, 64, 65], were also studied. The uniqueness of MXene’s properties makes them suitable for a variety of applications including but not limited to energy storage [66–72]; sensors including volatile organic compound (VOC) and biosensors [73–79] (employing antibodies [80], aptamers [81], enzymes [82], and nucleic acid [83]); photo- and electrocatalysis [84–90]; transparent electrodes/conductors [91–94]; photothermal therapy agents [81, 95, 96]; plasmonics [51, 97, 98]; thermoelectrics [99–101]; and water purification [102–106]. Furthermore, due to the ultra-thin thickness of their films, MXenes are good candidates for construction of high-performance engineered transistors and photoelectric devices [107–109].

## Synthesis of MXenes

The first MXene generation nanomaterials were synthesized using a selective etching of metal layers from the MAX phases, layered transition metal carbides, and carbonitrides using hydrofluoric acid [6] but alternative synthesis approaches are accessible now. These include selective etching in a mixture of fluoride salts [110] and various acids [111], non-aqueous etchants [112, 113], halogens [114], and molten salts [115], allowing to synthetize new MXenes with a better control over their surface chemistries.

MXenes can be produced in a range of forms from multilayer powders to inks of delaminated flakes [116] in water that in turn can be printed [117–119], sprayed [120–122], drawn into fibers [123, 124], or filtered into freestanding films [125–128]. MXenes’ hydrophilicity and ability to disperse easily in water without any surfactant simplify their processing. They are prone to oxidation at high temperatures and under oxidizing environments, which can lead to novel architectures of nanohybrid structures of oxides/carbon or oxide/carbon/MXenes with promising use as electrodes for energy storage and conversion.

MXenes are typically synthesized (derived) topochemically from their parent MAX phases via selective etching of the *A* element (Al, Si, or Ga). Synthesis of MXenes is a multi-step process. It starts with preparation of the precursor (MAX or another layered ceramic) often followed by etching and delamination in order to obtain a colloidal dispersion of single-layer MXene. MXenes are produced from layered ceramic precursors with four primary structures: M_2_AX, M_3_AX_2_, M_4_AX_3_, and M_5_AX_4_. There are many approaches for synthesizing MAX phases and other non-MAX precursors to MXenes, including high-temperature reaction of a powder mixture in a furnace [129–131], hot isostatic pressing [132–135], self-propagating high-temperature synthesis [136–140], microwave synthesis [141–144], molten metal synthesis [145–147], spark-plasma sintering [148–152], magnetron sputtering, and others [153–159], but preferentially high temperature synthesis is used.

The conversion from MAX to MXene (even in a multilayer form) leads to a distinct, visual color change: while MAX phases are usually gray in color, all MXenes will have their distinct colors which are related to their optical properties, depending on their structure and composition. With delaminated MXenes, the concentrated solutions appear to be black; however, when diluted (< 0.5 mg mL^−1^), a color specific to each MXene becomes apparent [38].

Early on, when the first generations of MXenes were prepared, such MXenes were all synthesized by selectively etching the Al layer from different MAX phases while modification of etching conditions such as acid concentration, temperature, and etching time for each MAX precursor allowed a limited control over the process. MXenes are multilayered materials with a morphology that resembles vermiculite clay—these multilayers are held together by a mixture of hydrogen and van der Waals bonds. This configuration allows to intercalate several chemicals between the layers, e.g., intercalation of dimethyl sulfoxide (DMSO, please note that DMSO is not intercalated into all types of MXenes) in Ti_3_C_2_T_*x*_. When such solutions are sonicated, the result is a colloidal solution of delaminated Ti_3_C_2_T_*x*_ dispersible in water. On the other hand through spontaneous intercalation of cations, large-scale delamination of various MXenes was achieved by intercalating large cations from organic phase solutions such as tetrabutylammonium hydroxide [160], choline hydroxide, and n-butylamine. Other groups have focused on the intercalation of increasingly large alkylammonium ions and other large structures into MXenes, often leading to unique properties of such nanomaterials [38]. Cation-intercalated engineering allows controlling the interlayer distance, which is directly proportional to the hydration size of the intercalated species, and tuning of the mechanical and actuation properties of Ti_3_C_2_ MXene. This in turn brings an enhancement of the capacitance and tunes interfacial properties for (bio)sensing purposes [39]. The surface chemistry (which depends on etching conditions), intercalated species, and even the flake size significantly affect MXene properties [16, 38, 161].

**Fig. 3:**
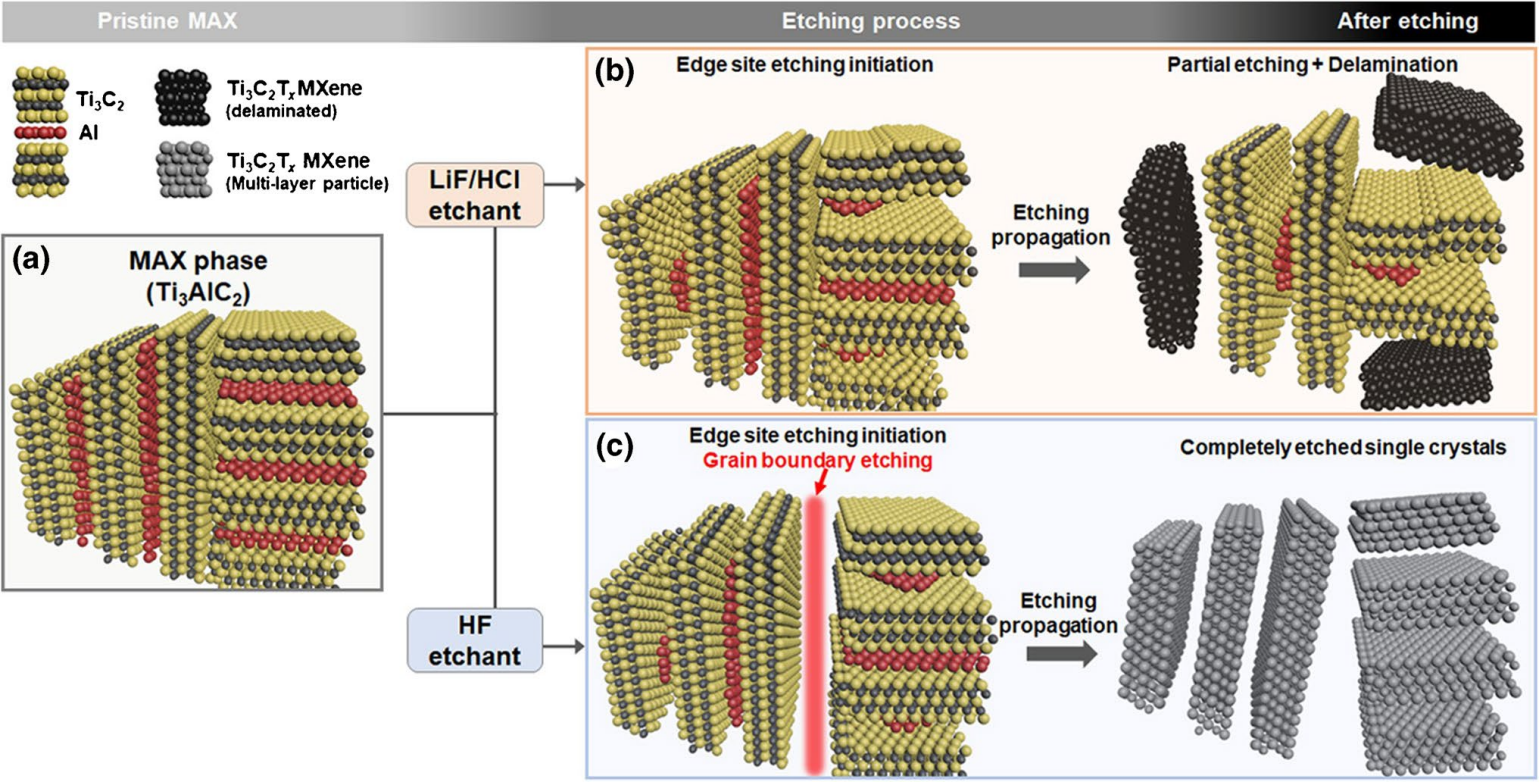
Schematic of the Al etching mechanism for LiF/HCl and HF solutions. (**a**) Polycrystalline particle of the pristine Ti_3_AlC_2_ MAX phase before the etching process. The etching mechanism for polycrystalline MAX particles in (**b**) LiF/HCl and (**c**) HF solutions. Reproduced with permission from ref. [38]. Copyright 2021 American Chemical Society

Microscopically, the etching behavior of the Ti_3_AlC_2_ MAX phase, when using different etchants, at the atomic scale has been studied by Naguib et al. [17] using focused ion beam and electron microscopy. They have looked at the structural changes in the Ti_3_AlC_2_ phase as a function of etching time and etchant type (LiF/HCl, HF, or NH_4_HF_2_) to reveal the etching mechanism for the first time. Apparently, the propagation of the etching front occurs in the direction normal to the inner basal plane of MAX phase for all etchants and it was revealed that HF and NH_4_HF_2_ etch the grain boundaries of polycrystalline MAX particles to expose more edge sites to the etchant, which is not observed for LiF/HCl etching pair. In contrast, for the LiF/HCl etchant, Li^+^ ions spontaneously intercalate between MXene layers, where they increase the interlayer spacing between MXene sheets and weaken their interaction, eventually resulting in delamination of the MXene sheets during the washing process after etching [17]. The scheme of the overall observed mechanism for etching monoatomic Al layers from Ti_3_AlC_2_ MAX depending on the type of etchant, LiF/HCl, or HF is demonstrated in Fig. 3.

Combination of fluoride salts such as LiF and more benign acids compared to HF such as HCl as etchants was a major breakthrough in the field. The in situ formation of HF not only converted the MAX phase to MXene, but the resulting product behaved like a clay from a rheological point of view and it could be processed into different shapes. Another optional etchants are, e.g., ammonium bifluoride (NH_4_HF_2_), hydrolyzed F–containing liquids, and molten fluoride salts*.* Other fluoride-free option for Ti_3_AlC_2_ includes aqueous electrolytes of 1.0 M ammonium chloride and 0.2 M tetramethylammonium, hydrothermal treatment by using 27.5 M NaOH at 270 °C, and iodine dissolved in anhydrous acetonitrile at 100 °C to form Ti_3_C_2_I_2_. Fluoride-free synthesis can also be achieved using a Lewis-acidic molten salt such as ZnCl_2_ or CuCl_2_ in the 500–750 °C temperature range, depending on the salt. Ti_2_SC can be thermally reduced to produce Ti_2_CT_*x*_. A salt-solution-based acoustic synthesis of Ti_3_C_2_T_*x*_ from Ti_3_AlC_2_ that utilized LiF in water with surface acoustic waves was shown to produce delaminated MXenes in seconds. Variations of etching conditions such as the ratio of fluoride salt to acid, or bubbling nitrogen gas during etching can change the properties of the resulting MXene significantly. MAX phase chemistry matters, e.g., having excess of Al during the synthesis of Ti_3_AlC_2_, will lead to the formation of highly stoichiometric MAX and MXene. There is a limited number of nitrogen containing MAX phases and synthesis of nitride MXenes is generally difficult, as the nitride layers tend to dissolve in the acids.

In summary, when aqueous HF is used, mixed = O, –OH, and –F interfacial terminations are usually found with different ratios, depending on the type of MXene and etching conditions. When molten chloride salts are used, –Cl terminations dominate; when water-free NH_4_HF_2_ is used, F-rich surfaces prevail. Moreover, electrochemical study confirmed a significant difference in the negative charge density on the surface of MXene and also in the electrocatalytic activity depending on the etchant (HF or in situ–generated HF from mixture of LiF and HCl) used in the preparation of MXenes [162].

MXenes are prone to oxidation at high temperatures and under oxidizing environments, which can lead to novel architectures of nanohybrid structures of oxides/carbon or oxide/carbon/MXenes that are found promising for use in electrodes for energy storage and conversion. It was shown that Ti_3_C_2_T_*x*_ begins to transform to cubic carbide with loss of surface oxygen at ~ 860 °C in a protective environment, and the thermal stability is somewhat dependent on the etching protocol [38]. A higher coverage by oxygen-containing species in combination with higher processing temperatures results in amorphization of the sheet and/or formation of TiO_2_ phases although the 2D nature of the flake persists. Finally, with extended oxidation at 450 °C, the MXene sheet was structurally transformed into crystalline titanium and amorphous Ti(CO)_2_ and while the MXene transforms into titanium layer, species such as H_2_O and CO_2_ are desorbed from the surface. MXenes are prone to intercalate and physisorb H_2_O; however, physisorbed water is weakly bonded and desorbs after heating above 200 °C [163]. MXene processing steps include exfoliation, size selection, concentration, and deposition. Processing begins with liquid-phase exfoliation. The MXene lateral flake size can be measured directly by microscopy methods or indirectly by dynamic light scattering (DLS). Colloidal stability can be measured by zeta potential (ζ-potential) through electrophoretic mobility measurements and since MXenes are negatively charged, the value of zeta potential is expected to be lower than − 30 mV in a wide range of pH values.

To measure chemical stability, one should determine how much of the material is degraded over time. V_2_CT_*x*_ or Ti_2_CT_*x*_ degrade quickly when dispersed in water and should be used immediately after synthesis [38]. Several studies demonstrated successful surface functionalization of Ti_3_C_2_T_*x*_ with carboxyl or glycine groups and silane coupling agents resulting in improvement of the Ti_3_C_2_T_*x*_ stability and charge percolation [39].

It is important to note that dense dry films have a much higher stability and a very long lifetime (years), unlike single-layer flakes in solution. There are multiple methods to deposit MXene on surfaces from a solution using vacuum-assisted filtration, spray-coating, spin-coating, dip-coating, drop casting, electrophoretic deposition, blade-coating, screen printing, inkjet printing, 3D printing, and electrospinning [38].

With versatility in MXene synthesis methods and suitable etching, MXenes can be easily transformed into quantum dots, nanosheets, and MXenes composites. Optical properties of MXenes enable biosensing applications, which are based on different optical transduction principles (e.g., photoluminescence, colorimetry, surface plasmon resonance, surface-enhanced Raman scattering, and electrochemiluminescence) [164]. Besides biosensors [165], MXenes found applications in luminescent imaging, diagnosis, photoacoustic imaging, computed tomography (CT) imaging, magnetic resonance imaging (MRI), therapy, drug delivery systems, photothermal therapy, photodynamic therapy, and immunotherapy, as antibacterial agents and in implants [165, 166].

A number of techniques are available to determine composition, structure, and properties of MXenes including energy-dispersive X-ray spectroscopy (EDS) [167], X-ray diffraction (XRD), X-ray photoelectron spectroscopy (XPS), Raman spectroscopy [168], scanning electron microscopy (SEM), and scanning transmission electron microscopy (STEM) [169]. Oxidation on the surface can be detected with Raman spectroscopy or XPS.

**Fig. 4:**
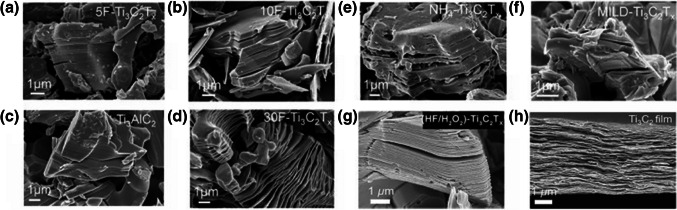
SEM images of MAX and MXene powders. Reproduced from an open access publication [166]. SEM images of multilayer Ti_3_C_2_T_*x*_ powders synthesized by etching with 30 wt% (**a**), 10 wt% (**b**), and 5 wt% HF (**c**). SEM images of Ti_3_AlC_2_ (MAX) powder (**d**). SEM images of Ti_3_C_2_T_*x*_ powders synthesized with ammonium hydrogen fluoride (**e**) and 10 M LiF in 9 M HCl (**f**). **a**–**f** Reproduced with permission from ref. [38]. Copyright 2021 American Chemical Society. SEM images of Ti_3_C_2_T_*x*_ generated by (HF/H_2_O_2_)-treated Ti_3_SiC_2_ (**g**). A cross-sectional SEM image of Ti_3_C_2_T_x_ films made by vacuum-assisted filtration of a colloidal solution of Ti_3_C_2_T_*x*_ in TMAOH (**h**). **g**–**h** Reproduced with permission from ref. [172]. Copyright 2018 John Wiley and sons

Basic characterization of MXenes is frequently carried out by scanning electron microscopy (SEM) as shown in Fig. 4 often complemented by EDS. Additional techniques of choice include pair distribution function analysis, X-ray absorption spectroscopy, and atomic force microscopy (AFM). For investigation of MXene composition, especially surface chemistry, X-ray photoelectron spectroscopy (XPS), Raman spectroscopy, electron energy loss spectroscopy, and nuclear magnetic resonance (NMR) are often applied. Moreover, secondary ion mass spectrometry (SIMS) was successfully applied, as well, providing mass spectra, 2D images, and depth profiles [170, 171]. Since EDS cannot distinguish between O and OH groups on the surface of MXenes, TEM instruments equipped with electron energy loss spectroscopy could be used for elemental analysis of MXenes.

On the other hand, XPS became the popular choice to determine the average material composition, due to its low penetration depth, thus surface sensitivity, and ability to acquire information about chemical composition and elemental oxidation states. The regions of interest with respect to MXenes are metal regions, O1s and C1s, and depending on synthesis method also F1s and Cl2p regions are present as well. Multiple oxidations states are possible, complex peak splitting can occur, and peaks can be asymmetric; for instance, the Ti 2p region of Ti_3_C_2_T_*x*_ is typically fit by multiple components, which represent various oxidation states of Ti (Ti^0^, Ti^2+^, Ti^3+^, Ti^4+^). The problem in XPS analysis can be the loss of water and OH terminations in high vacuum [38].

## Application of MXene-modified interfaces

The promising MXene nanomaterials, 2D layered carbides, and nitrides offering a number of alternative compositions, simple processing, relatively high yields and large flakes, hydrophilicity, metal-like electrical conductivity, rich functional groups, and unique optical properties have a profound effect on the entire field of material science. Furthermore, MXene Ti_3_C_2_T_*x*_ with redox active centers proved as an excellent electrochemical catalyst in, e.g., electrochemical reduction of H_2_O_2_, oxygen reduction reactions [170], and detection of small redox molecules [173]. In recent years, an immense increase in a number of affinity-based biosensors [174] employing MXene interfaces [175] has been observed. However, there is a need to pay attention to select appropriate strategies for patterning the MXene interface and subsequent immobilization of target biomolecules. Broad absorption band, favorable energy levels, and plasmon resonance in the visible or near-infrared range make MXenes promising candidates for optical, photothermal, and photoelectrochemical biosensing applications. For example Ti_3_C_2_ MXenes serve as fluorescence quenchers and SERS substrates [176].

In order to support the applicability of MXene-modified interfaces in biosensors, interfacial modification of the MXene should be implemented. To achieve this goal and prevent non-specific binding, the modification of Ti_3_C_2_T_*x*_ MXene interfaces by applying aryldiazonium-based grafting with derivatives bearing a sulpho-(SB) or carboxy-(CB) betaine pendant moiety was established [177]. Grafting of aryldiazonium-terminated molecules to MXene was possible due to presence of free electrons (plasmons) in MXene allowing a spontaneous reductive grafting of aryldiazonium-terminated molecules [177].

### Analysis of low molecular weight analytes

#### Glucose

Diabetes [178] is a chronic disease that causes high blood glucose levels, which can lead to a variety of serious health issues and therefore diligent and precise blood glucose monitoring becomes critical in the management and prophylaxis of hyperglycaemia [179]. Electrochemical glucose (bio)sensing is performed by either enzymatic biosensors or non-enzymatic sensors.

##### Non-enzymatic glucose sensing

Non-enzymatic glucose sensors are based on the use of many noble and transition metals such as Pt, Au, Ni, or Cu. The surface modifications of MXene additionally provide direct ion-exchange sites and plasmons within MXene can serve as stable reductant of metallic ions to form metal nanoparticles (NPs) on the surface of MXene. Enhanced surface area provides significant increase of the adsorption rates of the analyte species on the surface of the nanocomposites. To anchor metallic nanoparticles on the surface of MXenes, two strategies have been used: self-reduction and reduction of precursor metallic salt in the presence of an external reducing agent such as NaBH_4_, HCHO, and CO. The reduction of noble-metal ions without the need of an external reducing agent has attracted a lot of interest by forming nanoparticles made of Au, Pd, Pt, and Ag. Electro-reduction is still another way of reducing metallic salts to metallic nanoparticles [179].

Cupric oxide (CuO) NPs have been studied in conjunction with mono, double, and multilayered MXenes nanosheets for non-enzymatic glucose sensing applications. Additionally, due to strong electrostatic interactions, MXene-graphene hybrid composites can be easily synthesized by a simple mixing of the components [180].

Hu et al. prepared non-enzymatic MXene/chitosan/Cu_2_O electrode for simultaneous detection of glucose and cholesterol with LOD of 52.4 μM (the sensitivity of 60.3 μA·L/(mmol·cm^2^)) and 49.8 μM (the sensitivity of 215.71 μA·L/(mmol·cm^2^)), respectively [181].

**Fig. 5:**
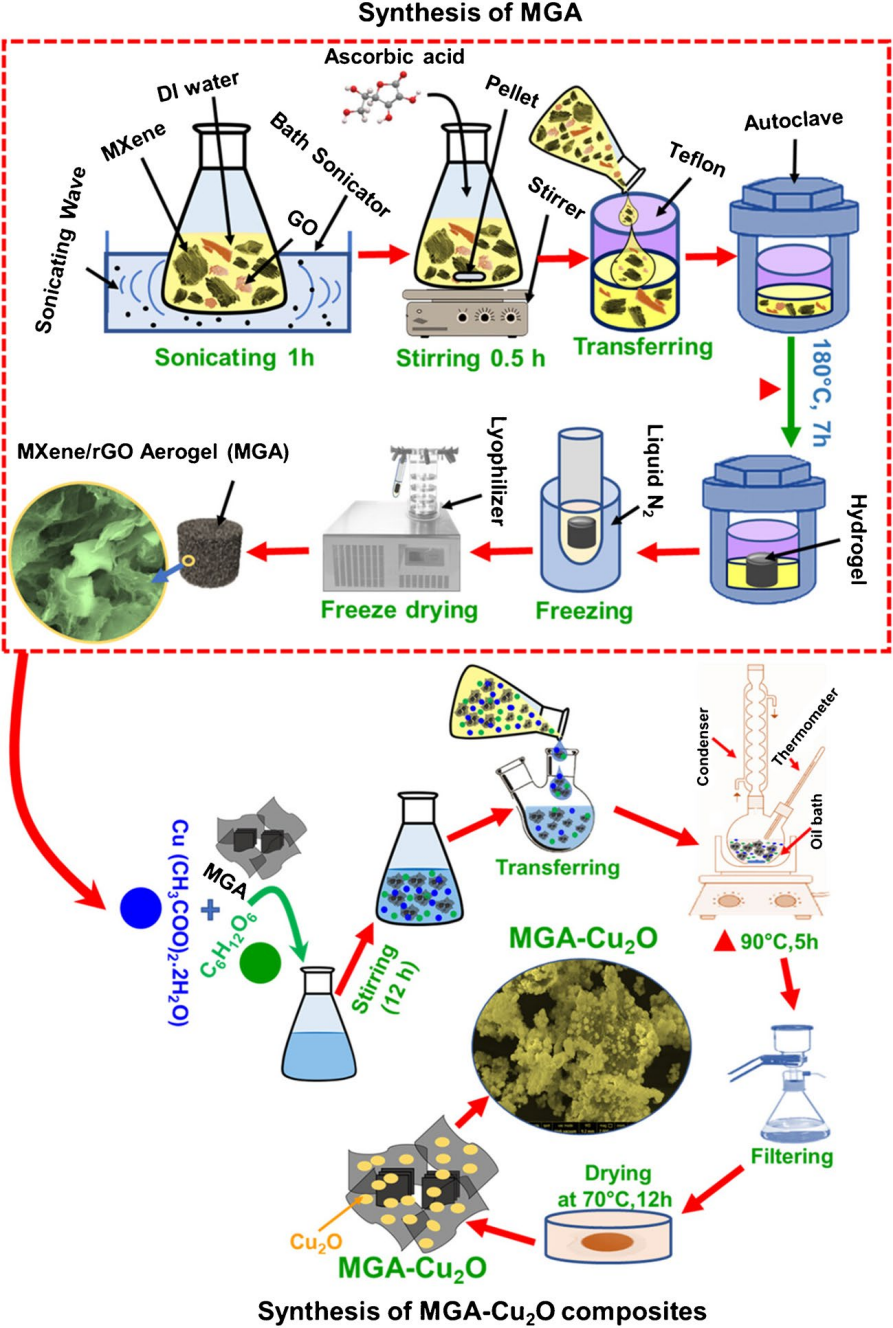
Scheme of MXene graphene aerogel synthesis and aerogel (MGA)–Cu_2_O composite synthesis. Reproduced with permission from ref. [182]. Copyright 2023 American Chemical Society

Alanazi et al. prepared a composite of aerogel based on MXene and reduced graphene oxide (rGO) nanosheets through hydrothermal method and subsequently added Cu_2_O by a coprecipitation method resulting in a 3D ternary composite with a large surface area and a porous structure (aerogel − Cu_2_O composite, Fig. 5) [182]. The fabricated electrode patterned by MXene/rGO/Cu_2_O as the nonenzymatic glucose sensor proved LOD of 1.1 μM and with two wide linear ranges of 0.1–14 mM and 15–40 mM [182].

##### Enzymatic glucose biosensing

Ti_3_C_2_T_*x*_ MXene nanosheet composites provide substantial surface area for enhanced enzyme immobilization, rapid electron transfer, and the availability of active redox centers. Generally speaking, MXene composites outperform bare MXenes as electrochemical sensors for glucose quantification. Enzymatic glucose biosensors are constructed using an active glucose oxidase (GOx), which catalyzes oxidation of glucose [160]. The selectivity and sensitivity of the enzymatic biosensors are strongly affected by the enzyme contamination, inadequate enzyme immobilization, and denaturation [179].

Delamination of MXene with tetrabutylammonium hydroxide (TBAOH) led to the formation of single and few layers thick MXene, which decreases the distance between the enzyme and the electrode as compared to the bulk and exfoliated counterparts. This allowed a faster electron transfer between the electrode and GOx enzyme. Restacking of the MXene layers is also impeded when MXenes and transition metal oxides are coupled, increasing the interfacial interaction between the electrolyte and electrode during electrochemical sensing analysis.

The amperometric glucose biosensor with the immobilized GOx on Nafion solubilized Au/MXene nanocomposite over glassy carbon electrode (GCE) was developed by Rakhi et al. [183]. The GOx/Au/MXene/Nafion/GCE biosensor detected glucose with a relatively high sensitivity of 4.2 μA mM^−1^ cm^−2^ and a detection limit of 5.9 μM with the linear concentration range from 0.1 to 18 mM [183].

**Fig. 6:**
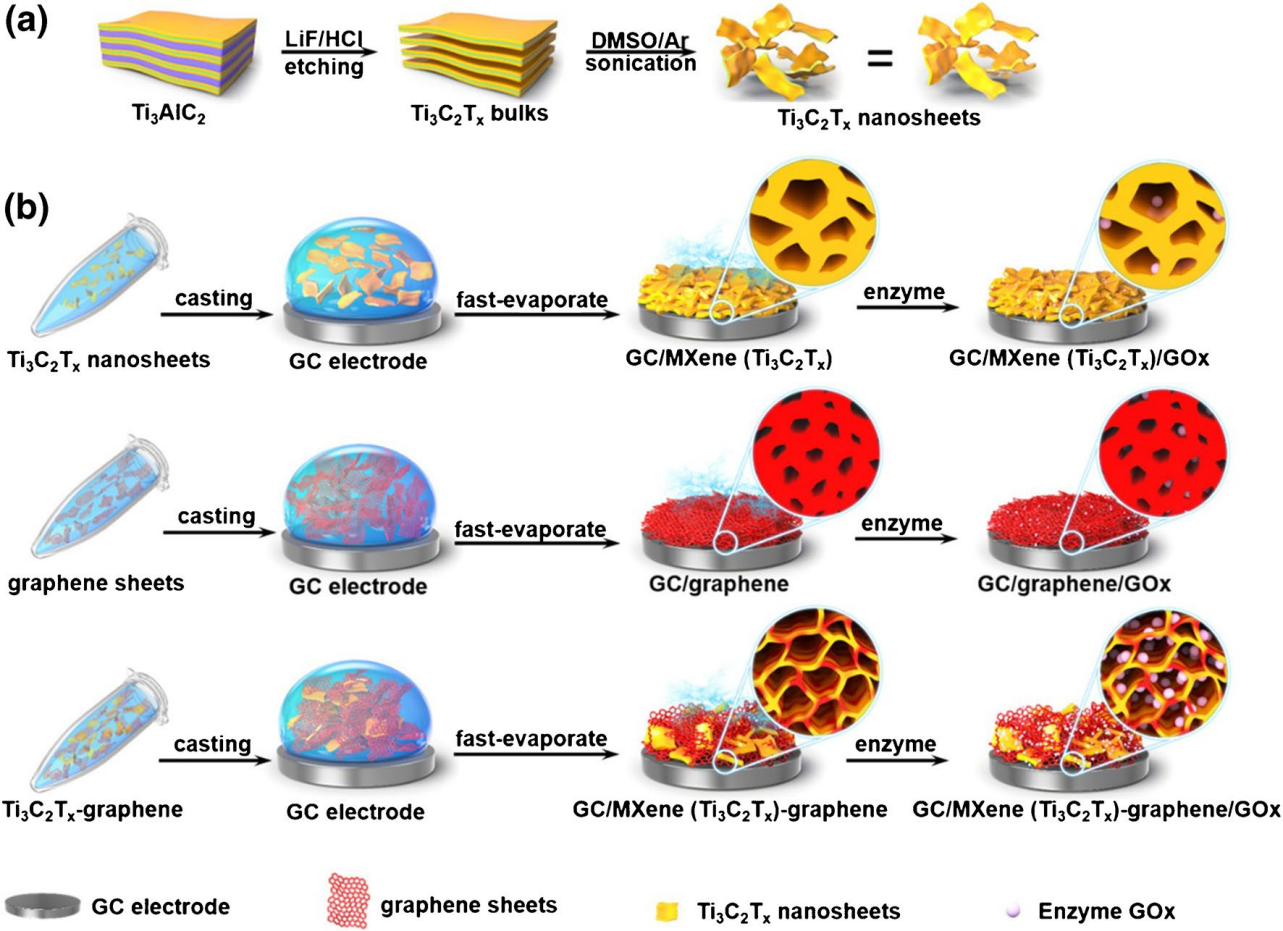
Construction of a glucose biosensor. Preparation of **a** Ti_3_C_2_T_*x*_ nanosheets; **b** pure Ti_3_C_2_T_*x*_ film, pure graphene film, and a hybrid film for enzyme immobilization. Reproduced with permission from ref. [184]. Copyright 2019 American Chemical Society

A 3D porous hybrid film, fabricated from Ti_3_C_2_T_*x*_ MXene and graphene sheets (weight ratio of 1:2 and 1:3), supplied an open structure to facilitate GOx entering the internal pores, which probably enhanced the stable immobilization and retaining of the GOx in the film (Fig. 6) [184]. As a result, the biosensor exhibited prominent electrochemical catalytic capability toward glucose biosensing, which was finally applied for glucose assay in sera. The detection limit of the biosensor in air-saturated and O_2_-saturated PBS was calculated to be 0.10 and 0.13 mM, respectively. The proposed biosensor revealed high specificity for glucose analysis over the potential interference species present in biological systems including amino acids, active biological species, and metal ions [184].

Murugan et al. fabricated an enzymatic biosensor by immobilization of GOx using chitosan onto a composite modified electrode [185]. An amperometric biosensor determined glucose with the LOD of 22.5 µM within a linear range of 0.5–8 mM. Further, a good reproducibility after continuous use of the biosensor for 20 days was demonstrated [185].

Gao et al. boosted the long-term stability of the enzyme biosensors employing sodium hyaluronate as a protective/biocompatible film, MXene-Ti_3_C_2_/GOx as the reaction layer, and chitosan/rGO film as the adhesion layer [186]. The practical and simple hyaluronate protective layer offered high biocompatibility and could be also applied for construction of other types of biosensors. The layered structure could effectively enhance the fixation between the active layer and the electrode, improving electron transfer between the enzyme and the electrode [186].

Laser scribing of porous graphene electrodes on flexible substrates is another option for developing disposable electrochemical biosensors. A CO_2_ laser scribing process was performed under ambient conditions to produce the porous graphene electrodes from lignin [187]. The obtained nitrogen doped laser-scribed graphene is a binder-free, hierarchical, and conductive while the interconnected carbon network displayed enhanced electrochemical activity with improved heterogeneous electron transfer rate. Furthermore, the electrodes were decorated with MXene/Prussian blue composite via a simple spray-coating process, designed for sensitive detection of analytes. The final electrodes were functionalized with catalytic enzymes for detecting glucose, lactate, and alcohol. The enzyme electrodes exhibited remarkably enhanced electrochemical activity toward the detection of the analytes. Such types of devices have high potential for applications in personalized healthcare, opening the door toward point-of-care monitoring and personalized sensors [187]. Methods like drop-casting, inkjet printing, screen printing, direct pencil drawing, the laser scribing process, and wire or fiber attachment were developed to obtain miniaturized electrodes on paper substrates—an alternative to advanced laboratory instruments, especially for use in remote regions, for emergencies, or for home healthcare applications. These are perfect candidates for analysis of glucose, lactate, and alcohol present in sweat. In order to detect *diabetes mellitus*, detecting glucose from sweat has been performed by immobilizing GOx onto a patterned electrode. Glucose could be detected down to 0.3 μM (sensitivity of 49.2 μA mM^−1^ cm^−2^) and lactate down to 0.5 μM (sensitivity of 21.6 μA mM^−1^ cm^−2^). Hence, a multianalyte detection was demonstrated from a single sweat sample using a low-cost approach avoiding additional material waste [187].

##### Wearable glucose (bio)sensors

For diabetes treatment, continuous glucose monitoring provides an efficient, real-time, and long-term self-monitoring technique using a wearable device that gives glucose measurements from the interstitial fluid at predetermined regular time intervals. Such a device is usually composed of three parts: a sensor, a transmitter, and a receiver (or a smart device app). The data from the sensor are sent to the transmitter, which then send them to a receiver or a smart device app. The term non-invasive and continuous glucose monitoring using MXene-based glucose biosensors describes measurement of human blood glucose without inflicting tissue damage. The idea comes from the fact that, in addition to glucose in human blood, significant amount of glucose is also found in other body fluids like saliva, tears, sweat, urine, and interstitial fluids. Wearable sensors can be easily affixed to the skin for real-time, continuous, and out-of-clinic health monitoring.

**Fig. 7:**
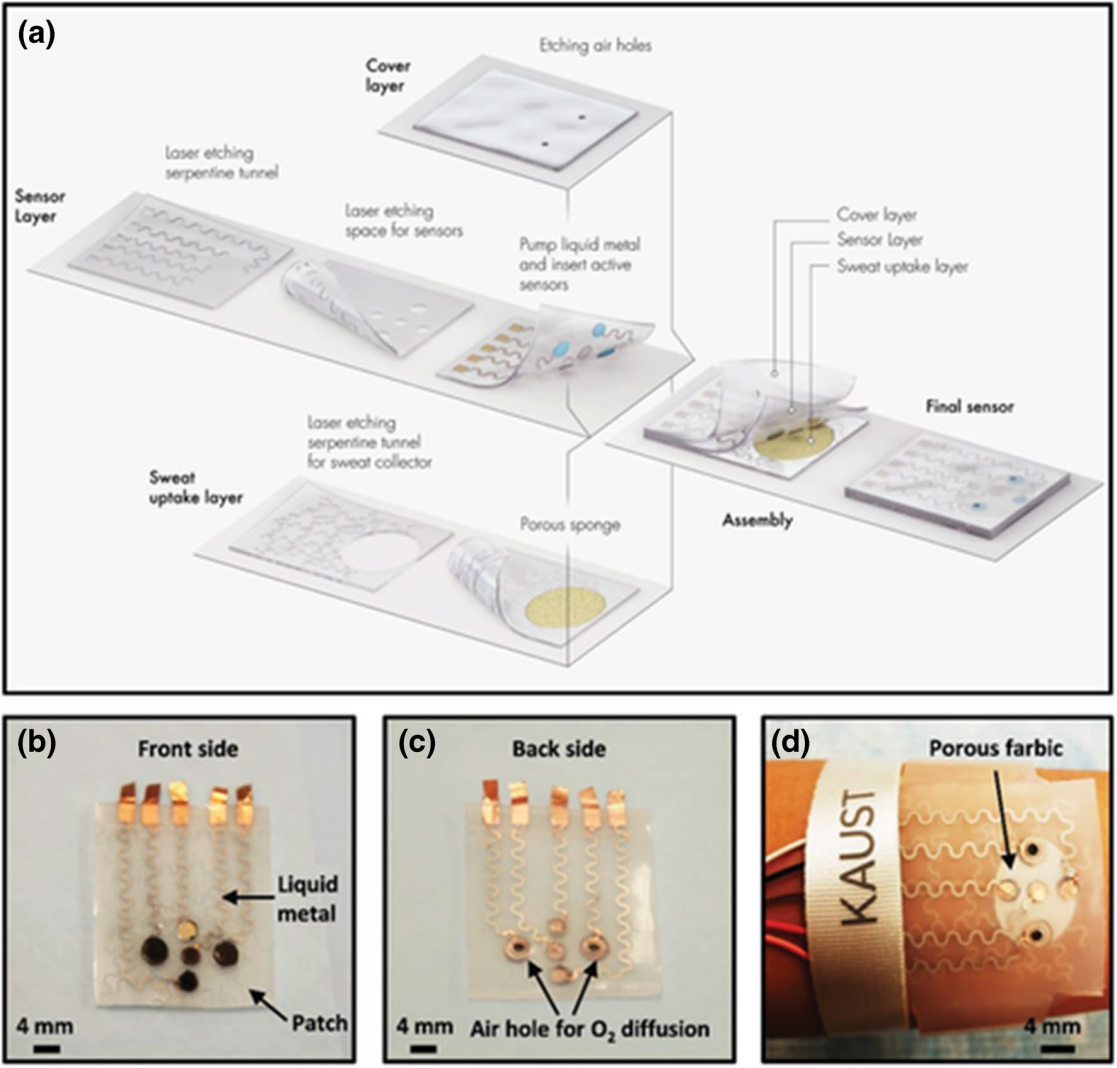
Schematic drawings and corresponding images of the wearable biosensor patch. **a** Schematic illustration of the sensor patch system, which is composed of a sweat-uptake layer, a sensor layer, and a cover layer. **b** Front-side optical image of the sensor array (left and right), reference electrode (top), counter electrode (middle), and pH sensor (bottom). **c** Back-side optical image of the sensor array. **d** Optical images of the sensor wristband laminated on human skin. Reproduced with permission from ref. [188]. Copyright 2019 John Wiley and Sons

For instance, the development of a stretchable, wearable, and modular multifunctional biosensor has been reported comprising MXene/Prussian blue composite for a long-term and sensitive detection of glucose and lactate metabolites in sweat (Fig. 7) [188]. Sweat-based sensing still poses several challenges, including easy degradation of enzymes and biomaterials with repeated testing, limited detection range, and sensitivity of enzyme-based biosensors caused by oxygen deficiency in sweat, and a poor stability of biosensors using all-in-one working electrodes patterned by traditional techniques (e.g., electrodeposition and screen printing).

A novel stretchable, wearable, and modular multifunctional biosensor was developed, incorporating a innovative composite designed for durable and sensitive detection of biomarkers (e.g., glucose and lactate) in sweat. The implemented solid–liquid–air three-phase interface design led to superior sensor performance and stability. Typical electrochemical sensitivities of 35.3 μA mM^−1^ cm^−2^ for glucose and 11.4 μA mM^−1^ cm^−2^ for lactate were achieved using artificial sweat. Terminal groups like –OH could be introduced into MXene structures, offering the possibility of immobilizing biological recognition proteins in an oriented way. The applied MXene increased immobilization efficiency of immobilized enzyme and permeability of oxygen into a biosensing layer. These sensors were integrated within flexible polymeric structures and used as wearable biosensing devices for the determination of lactose and glucose in a concentration range of 1–20 mM [188].

**Fig. 8:**
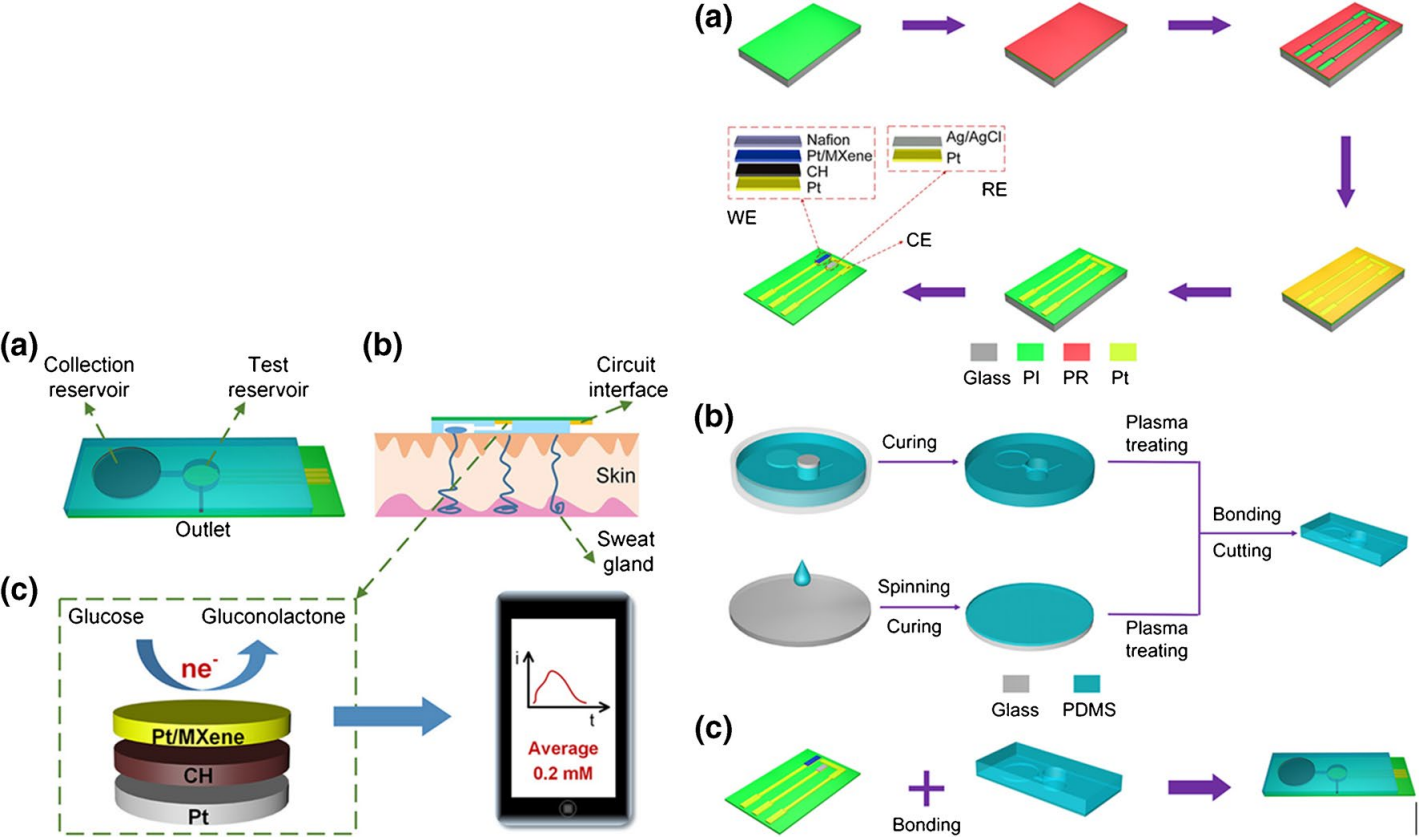
**a** Conceptual scheme of the proposed flexible wearable non-enzymatic continuous glucose detection sensor. (**b**) Cross-sectional view of the proposed flexible wearable sensor on skin. (**c**) Diagram of oxidation reaction with glucose on Pt/MXene interface (left image). (**a**) Fabrication process of the flexible sensor. (**b**) Fabrication process of the microfluidic patch. (**c**) Integration of the flexible sensor and microfluidic patch (right image). Reproduced with permission from ref. [189]. Copyright 2023 American Chemical Society

Li et al. developed a flexible wearable non-enzymatic electrochemical sensor for personalized diabetes treatment and management via glucose detection in sweat [189]. The sensor consisted of Pt/MXene nanocomposite immobilized onto a conductive hydrogel and microfluidic patches (Fig. 8) that were seamlessly integrated to improve the robustness and stability of the electrochemical sensors. Glucose was determined with LOD of 29.15 μmol L^−1^ and sensitivity of 3.43 μA mM^−1^ cm^−2^ in a linear concentration range of 0−1 mM (S/N = 3) by a chronoamperometric method [189].

#### Biosensors for analysis of other low molecular weight analytes

Continuous measurements of a wide range of chemicals/biomolecules in vivo are of great significance since real-time data are key indicators providing clinicians a valuable window into patients’ health and their response to therapeutics. Electrochemical sensors, due to their low cost, easy operation, high sensitivity, etc., are a suitable candidate device for continuous biomarker measurement, wherein modification of electrodes with other agents is beneficial and even indispensable to enhance and ensure sensing performance.

**Fig. 9:**
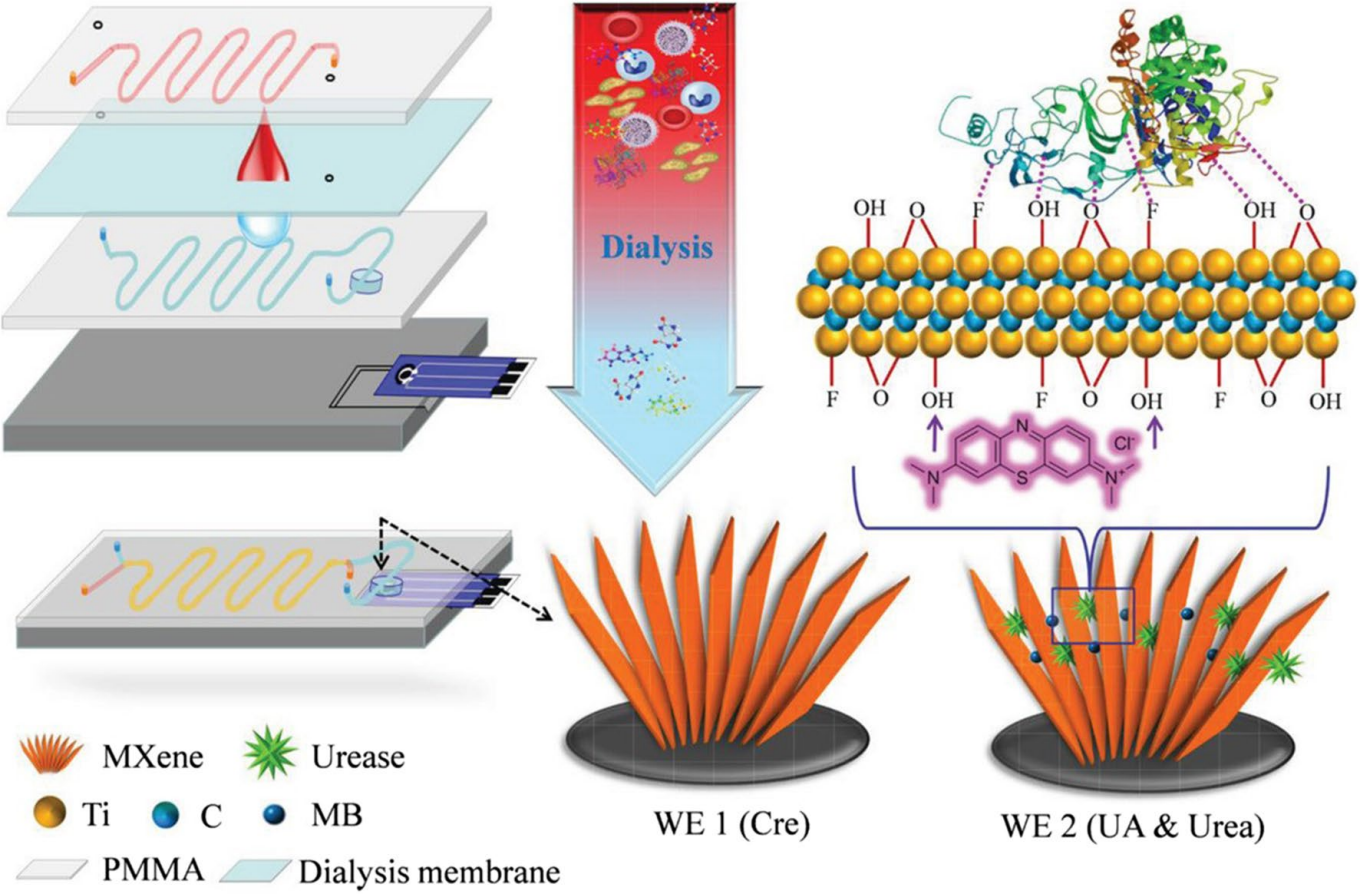
Schematic illustration showing fabrication of the MXene-based microfluidic chip. Reproduced with permission from ref. [190]. Copyright 2019 John Wiley and Sons

Using MXene-modified screen-printed electrode (SPE) in a microfluidic chip, continuous measurement of multiple analytes was realized and the sensor system featured miniaturization and automatization [190]. In one instance, MXene-Ti_3_C_2_T_*x*_-based SPE incorporated with a dialysis microfluidic chip was constructed for a direct and continuous multicomponent analysis of whole blood. The three biomarkers (uric acid, urea, and creatinine) in renal function examination were tested as model analytes by using the newly developed sensor. These analytes are also important indicators for patients with severe kidney injury and requiring hemodialysis treatment. The chip consisted of four layers, the channel in the top layer is set aside for blood flow, and the second layer is a dialysis membrane that allows penetration of molecules smaller than 1000 Da, like urea, uric acid, and creatinine (Fig. 9). Subsequently, the third layer contained the flow channel for isotonic solutions and the detection chamber. The analytes in blood can be dialyzed into this channel and gathered in the detection chamber, and the sensing electrode located in the bottom layer could capture these targets and generate the signals. Urea was detected with the average sensitivity of ~ 0.34 μA μM^−1^ with LOD (S/N = 3) of 5 × 10^−6^ M. Creatinine was analyzed in the range of 10–400 × 10^−6^ M with LOD down to 1.2 × 10^−6^ M (S/N = 3). Multicomponent detection proved to be accurate, reliable, and interference-free method, which can perfectly meet the clinical and user requirements. Moreover, the microfluidic chip also showed the great potential as a promising assay device for point-of-care test in terms of cost, stability, adaptability in different/adverse detection environments, miniaturization, and automation of the tests [190].

**Fig. 10:**
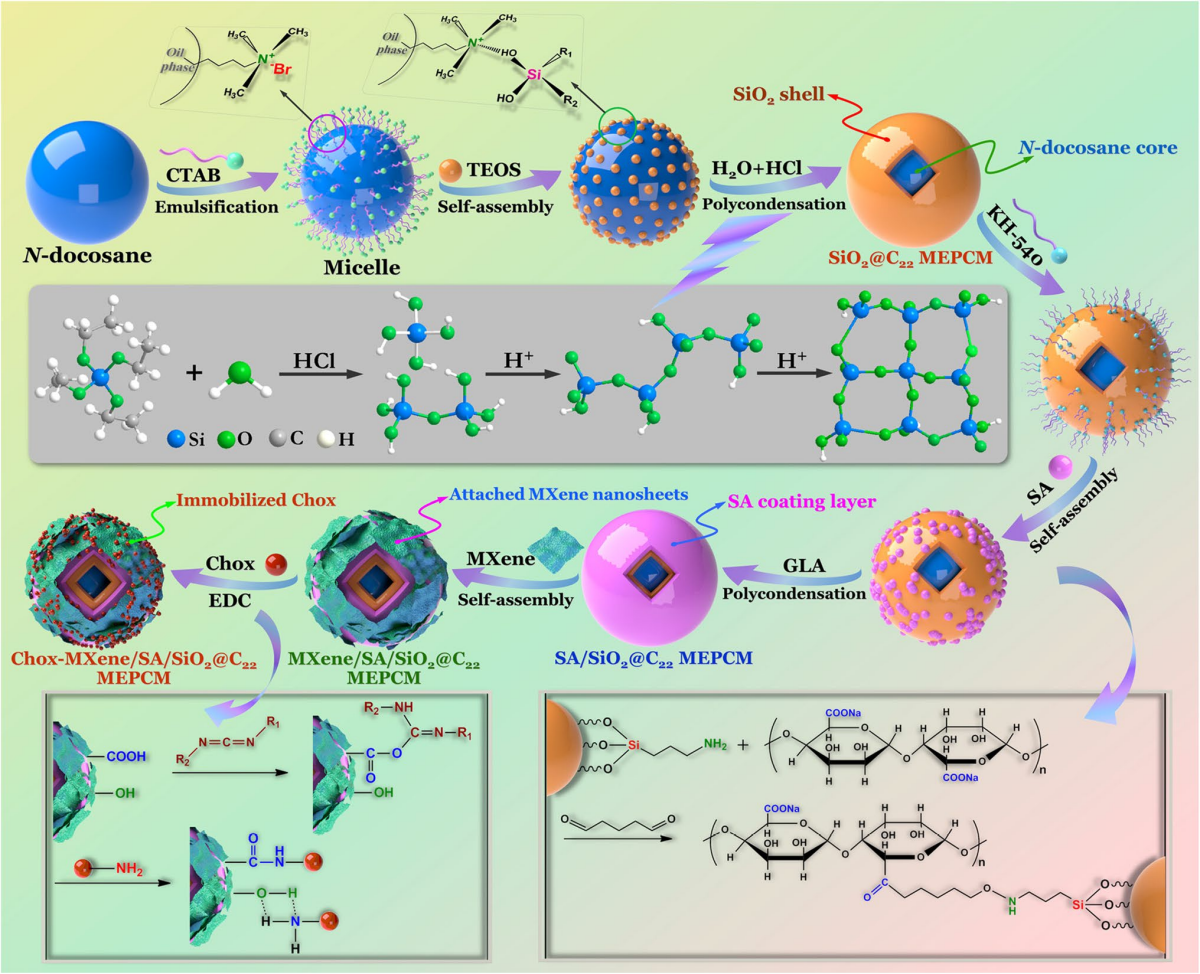
Schematic fabrication strategy for construction of a cholesterol biosensor. Reproduced with permission from ref. [191]. Copyright 2023 Royal Society Chemistry Publishing

Zhang et al. [191] have developed cholesterol oxidase-immobilized MXene/sodium alginate/silica@ n-docosane hierarchical microcapsules as a thermoregulatory electrode material to design electrochemical biosensors to meet the requirement of ultrasensitive detection of cholesterol at high temperature (Fig. 10). The developed biosensor achieved a higher sensitivity of 4.63 µA mM^−1^ cm^−2^ and a low LOD of 0.081 mM at high temperature, providing highly accurate and reliable detection of cholesterol for real biological samples over a wide temperature range [191].

In the work of Xu et al. [192], a biosensor for determination of H_2_O_2_ was prepared using an horseradish peroxidase (HRP)/Ti_3_C_2_/Nafion film-modified GCE. The biosensor offered a wide linear range (5–8000 μM) and low LOD of 1 μM (S/N = 3). The biosensor was used to detect H_2_O_2_ in clinical serum samples of normal controls and patients with acute myocardial infarction before and after percutaneous coronary intervention [192].

Three-dimensional (3D) porous laser-scribed graphene is a potential electrode material for construction of flexible electrochemical sensors due to its high efficiency and low cost [193]. 2D MXene nanosheets were applied to functionalize 3D laser-scribed graphene sheets with a C–O–Ti covalent crosslink obtaining a hybrid scaffold. As a proof of concept, the obtained hybrid nanocomposite was used to detect ascorbic acid (10–1600 μM), dopamine (12–240 μM), and uric acid (8–100, 200–800 μM) with low detection limits achieved, i.e., 3 μM for ascorbic acid, 0.13 μM for dopamine, and 1.47 μM for uric acid [193].

A photoreduction technique was used to increase the surface enhanced Raman spectroscopy (SERS) activity of MXene and to increase the ability to detect antipsychotic drugs [194]. Due to a cooperative action of chemical and electromagnetic mechanisms, MXene anchored with gold nanoparticles (AuNPs) caused a strong SERS amplification. The platform was used to detect chlorpromazine with LOD of 3.92 × 10^−11^ M in a wide linear range of 10^−1^–10^−10^ M [194].

**Fig. 11:**
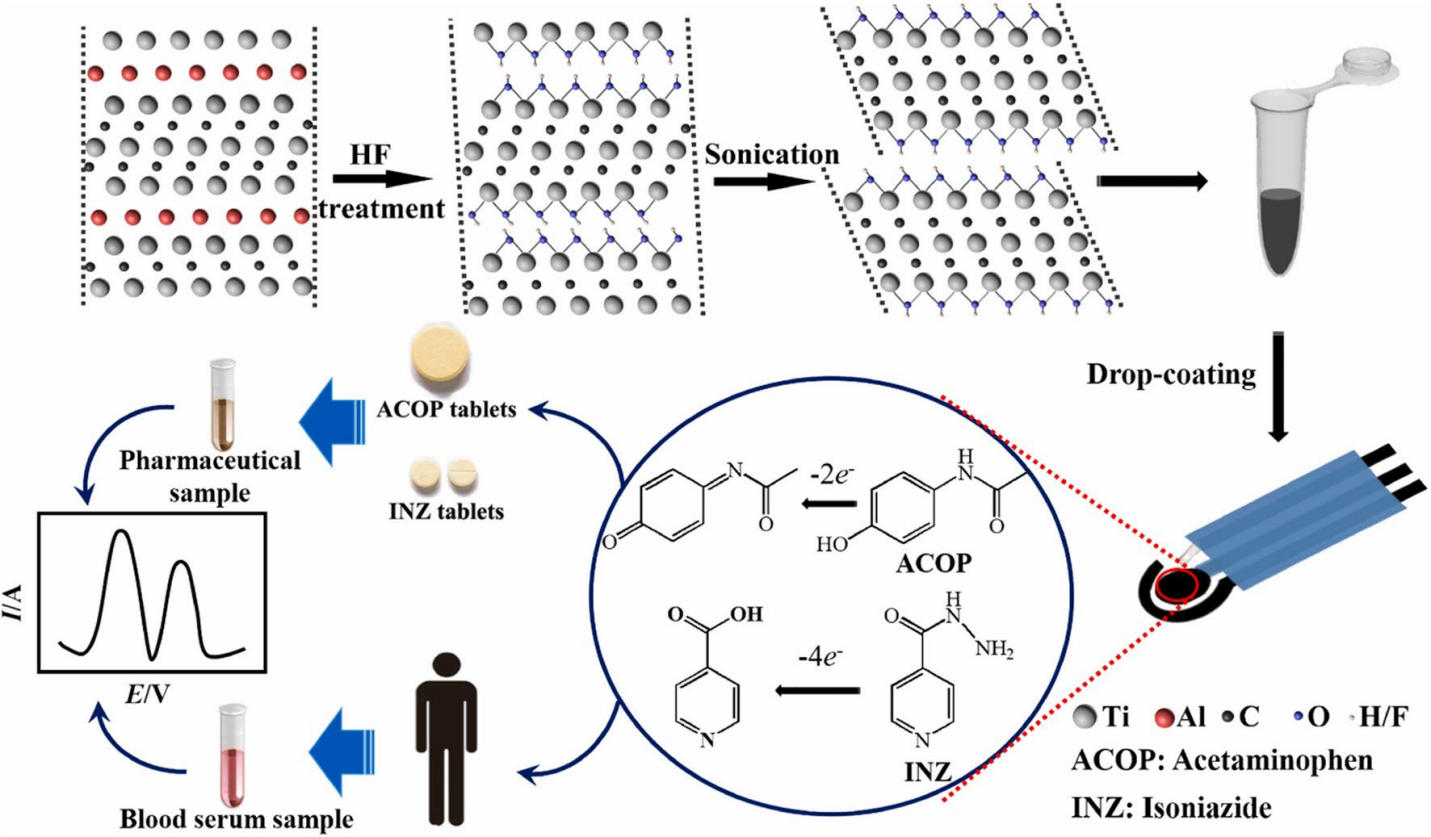
Schematic representation of MXene synthesis process, mechanism of electrocatalytic oxidation, and the utilization of MXene/SPE sensor for the detection of acetaminophen and isoniazid. Reproduced with permission from ref. [195]. Copyright 2019 Elsevier

The ordinary used drugs such as acetaminophen and isoniazid were simultaneously determined by applying disposable, miniaturized and portable MXene-modified SPE (Fig. 11) with LOD of 0.048 μM (linear range of 0.25–2000 μM) and 0.064 mM (linear range of 0.1–4.6 mM), respectively [195].

**Fig. 12:**
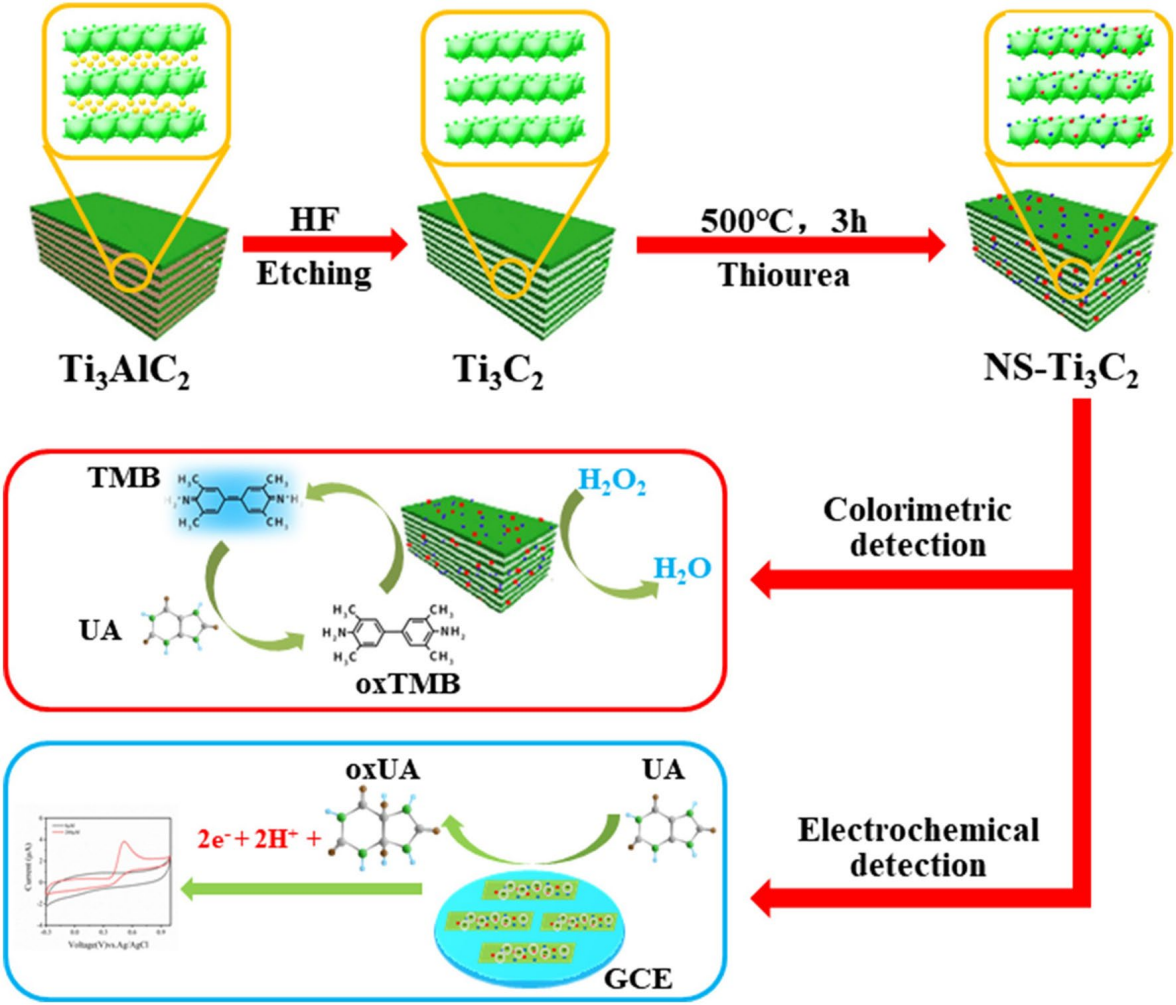
Schematic illustration of the synthesis and application of Ti_3_C_2_ nanosheets. Reproduced with permission from ref. [196]. Copyright 2022 Elsevier

Chen with co-workers coupled benefits of colorimetry and electrochemical methods to distinguish uric acid with LOD of 0.19 μM in the linear range of 2–400 μM [196]. The peroxidase-like activity and electrocatalytic activity of nitrogen and sulfur co-doped Ti_3_C_2_ nanosheets (Fig. 12) were successfully proved by the dissociation and adsorption of H_2_O_2_ and by the protonation of H_2_O_2_-containing peroxidase substrate 3,3′,5,5′-tetramethylbenzidine (TMB) [196].

**Fig. 13:**
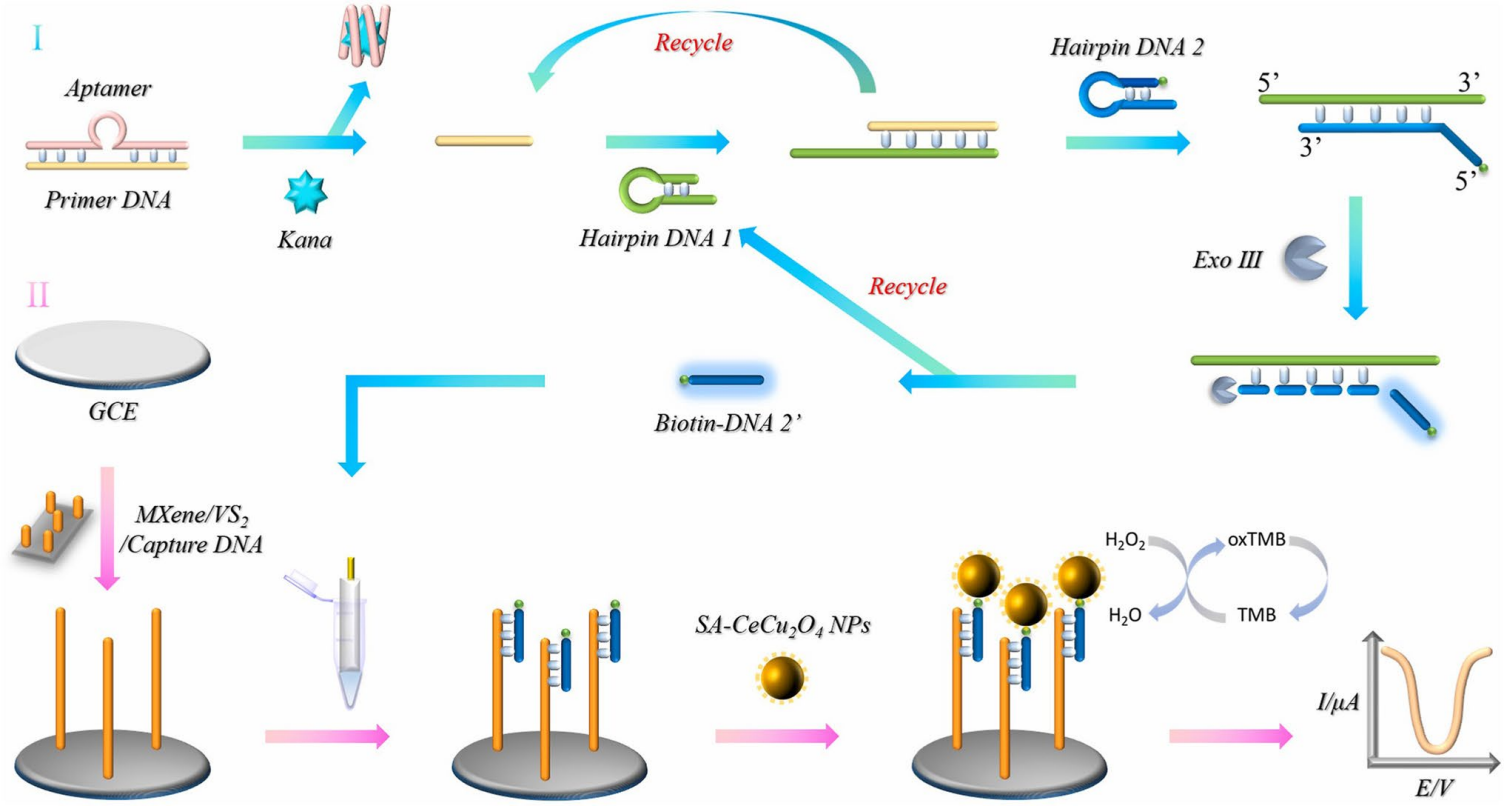
Schematic illustration showing preparation of the biosensor and the electrochemical detection strategy for analysis of kanamycin. Reproduced with permission from ref. [197]. Copyright 2023 Elsevier

The signal amplification sensing strategy relying on the electrode surface area modified with MXene/VS_2_ nanocomposite and CeCu_2_O_4_ bimetallic nanoparticles as nanozyme was performed by Tian et al. (Fig. 13) [197]. Kanamycin presenting an aminoglycoside antibiotic and effectively inhibiting Gram-positive and Gram-negative bacteria was detected with a high specificity by profiling five other antibiotics, with LOD of 0.6 pM (linear range from 5 pM to 5 μM) [197].

A nonsteroidal, estrogenic mycotoxin zearalenone was detected by SPE coated with MXene/chitosan layer with LOD of 0.4 pg mL^−1^ [198].

An enzymatic biosensor composed of Ti_3_C_2_T_*x*_ nanosheets and β-hydroxybutyrate dehydrogenase was able to determine β-hydroxybutyrate used for the diagnosis of diabetic ketoacidosis/diabetic ketosis with LOD of 45 μM and a sensitivity of 0.480 μA mM^−1^ cm^−2^ (a linear range of 0.36–17.9 mM) [199].

Further, Elumalai et al. applied a label-free AuNP@Ti_3_C_2_T_*x*_ nanocomposite patterning GCE electrode to detect simultaneously uric acid and folic acid. LODs of 11.5 nM for uric acid (a linear range of 0.03–1520 μM) and 6.20 nM for folic acid (a linear range of 0.02–3580 μM for FA) were reached, respectively [200].

#### Biosensors for detection of high-molecular weight analytes

As a proof of concept, MXene@PAMAM-based nanobiosensing platform was applied to develop an immunosensor for detecting human cardiac troponin T [201]. A fast, sensitive, and highly selective response toward the target in the presence of a [Fe(CN)_6_]^3−/4−^ redox marker was realized, ensuring a wide detection range of 0.1–1000 ng mL^−1^ with a LOD of 0.069 ng mL^−1^. Moreover, the sensor’s signal only decreased by 4.38% after 3 weeks, demonstrating that it exhibited satisfactory stability and better results than previously reported MXene-based biosensors [201].

A sensitive dual-signal sandwich-type electrochemical immunosensor was designed for neutrophil gelatinase–associated lipocalin detection using a square wave voltammetry (SWV) and current–time (*i*–*t*) curves [202]. MXene-loaded polyaniline nanocomposites were fabricated and utilized as the sensing platform for anchoring AuNPs and immobilizing primary antibodies. The biosensor exhibited optimal analytical performance in the linear range of 0.00001–10 ng mL^−1^ with LODs of 0.0074 pg mL^−1^ (SWV) and 0.0405 pg mL^−1^ (*i* − *t*) for the analyte determination [202].

**Fig. 14:**
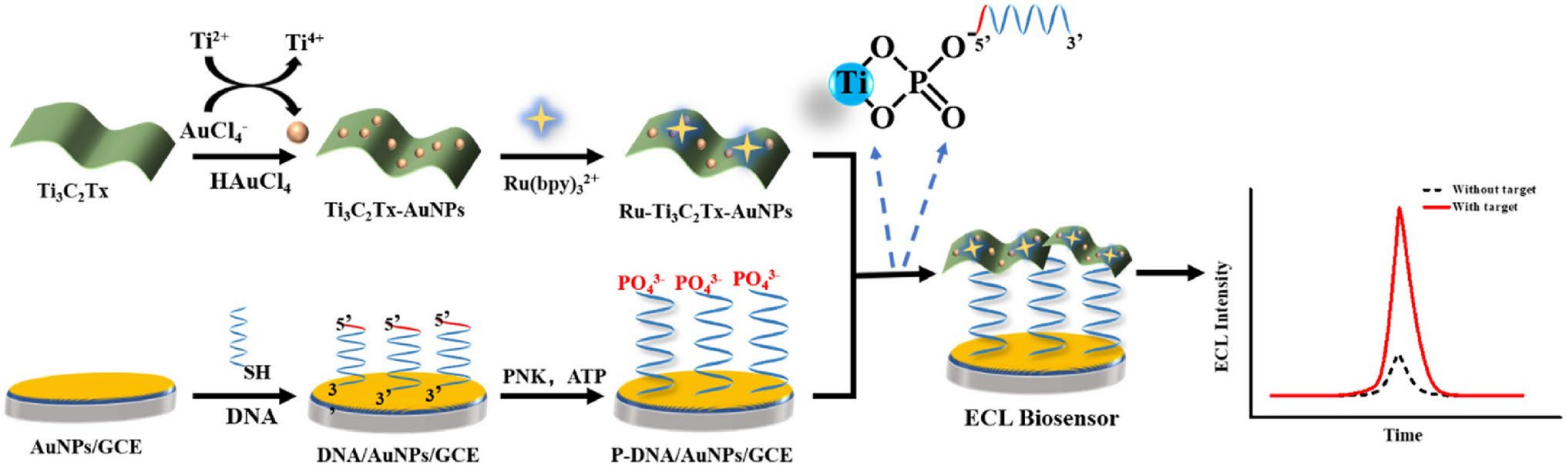
Schematic representation of the fabrication and function of the electrochemiluminescence biosensor for detection of polynucleotide kinase activity based on the Ti_3_C_2_T_*X*_ nanosheets. Reproduced with permission from ref. [203]. Copyright 2022 Elsevier

The abnormal expression of polynucleotide kinase, an enzyme playing a crucial role in phosphorylation-related DNA repair, can lead to cardiovascular disease, central nervous system disorders, Rosemond-Thomson syndrome, etc. For this purpose, Wang et al. proposed electrochemiluminescence biosensor based on Ti_3_C_2_T_*X*_ nanosheets patterned by AuNPs and Ru(bpy)_3_^2+^ (Fig. 14) [203]. The DNA phosphorylated by the enzyme was successfully recognized by the chelation between Ti and phosphate group with LOD of 0.0002 U mL^−1^ and with a linear range from 0.002 to 10 U mL^−1^ [203].

The electrochemical rat liver microsome biosensor employing Au@MXene nanocomposite determined aflatoxin B1, carcinogenic, embryotoxic, mutagenic, teratogenic, and hepatotoxic metabolite to humans, with LOD of 2.8 nM in the linear range of 0.01–50 μM [204].

2D MXene together with bovine serum albumin previously denatured by urea resulted in the anti-fouling sensing surface for IgG determination with LOD of 23 pg mL^−1^ and offering a linear concentration range of 0.1 ng mL^−1^–10 μg mL^−1^ [205].

Beta-human chorionic gonadotropin (β-hCG) was detected through the Ag/Ti_3_C_2_T_*x*_-based immunosensor with LOD of 9.5 × 10^–3^ mIU mL^−1^ in a linear range of 5.0 × 10^–2^–1.0 × 10^2^ mIU mL^−1^ [206].

### Biosensors for detection of cancer biomarkers

Cancer diseases present an enormous problem with 19.3 million new cancer cases and 10.0 million cancer-associated deaths worldwide in 2020 and the number of deaths will increase by 47% by 2040 [207]. Thus, there is high demand for ultrasensitive and selective sensing platforms able to detect cancer biomarkers down to very low levels.

The (bio)sensors based on functionalized MXene surface due to their specific properties and complex layered structure in combination with electrochemical methods allow achieving low LOD and high specificity of analysis [208]. MXene-enabled electrochemical aptasensors have shown great promise for the cancer biomarkers detection with LODs down to fM level [209].

The 2D MXene-based interfaces with a large surface area are suitable for glycoprofiling of cancer biomarkers or glycans (complex carbohydrates). The efficient MXene-cartridge-based columns for specific and selective enrichment of cancer-associated sialylated and bisecting *N*-glycans present in complex serum samples were utilized [210].

#### Small molecules

Sarcosine, *N*-methylglycine, presents an intermediate metabolite involved in glycine synthesis and degradation. The correlation between changed sarcosine levels and prostate cancer was referred in a number of studies [211, 212]. Since significantly elevated levels of sarcosine can be present in urine (from 20 nM to 5 µM), urine is the biofluid of choice allowing non-invasive detection of cancer biomarker. The amperometric miniaturized portable enzymatic nanobiosensor for the ultrasensitive analysis of sarcosine was designed [213]. Disposable screen-printed carbon electrodes together with MXene Ti_3_C_2_T_*x*_@chitosan composite and sarcosine oxidase provided a reliable, sensitive, and quick detection nanoplatform. A satisfactory LOD value of 10.4 nM was achieved by the biosensor during measurement in a drop of 100 μL. The as-fabricated biosensor had shown a good stability with only a 6.8% decrease in a current response within a period of at least 5 weeks after its preparation [213].

Moreover, an enzymatic biosensor based on Ti_3_C_2_T_*X*_/Pt–Pd nanocomposite developed by Ran et al. was able to detect sarcosine with LOD of 0.16 μM and a sensitivity of 84.1 μA mM^−1^ cm^−2^ with a linear range of 1–1000 μM [214].

#### DNA/RNA and microRNA

2D MXene nanosheet–anchored AuNP-decorated biomimetic bilayer lipid membrane biosensor was introduced for the attachment of thiolated single-stranded DNA for detection of DNA [215]. The biosensor gave hybridization signals to the complementary DNA sequence within a linear range from 10 zM to 1 μM with LOD of 1 zM. The BRCA1 gene mutation related to breast cancer was successfully detected [215].

The label-free electrochemical biosensor combining MXene-MoS_2_ heteronanostructure with a catalytic hairpin assembly amplification approach was applied for detection of microRNA-21 [216]. Thionine together with AuNPs was applied for patterning the surface of MXene-MoS_2_ heteronanostructure. The biosensor exhibited LOD of 26 fM and could be applied for detection of microRNA-21 in a concentration range from 100 fM to 100 nM [216].

The novel electrochemical biosensor amplified with hierarchical flower-like gold, poly(n-butyl acrylate), and MXene nanocomposite and activated by highly special antisense single-stranded DNA determined miRNA-122 with unprecedented LOD of 0.0035 aM [217].

**Fig. 15:**
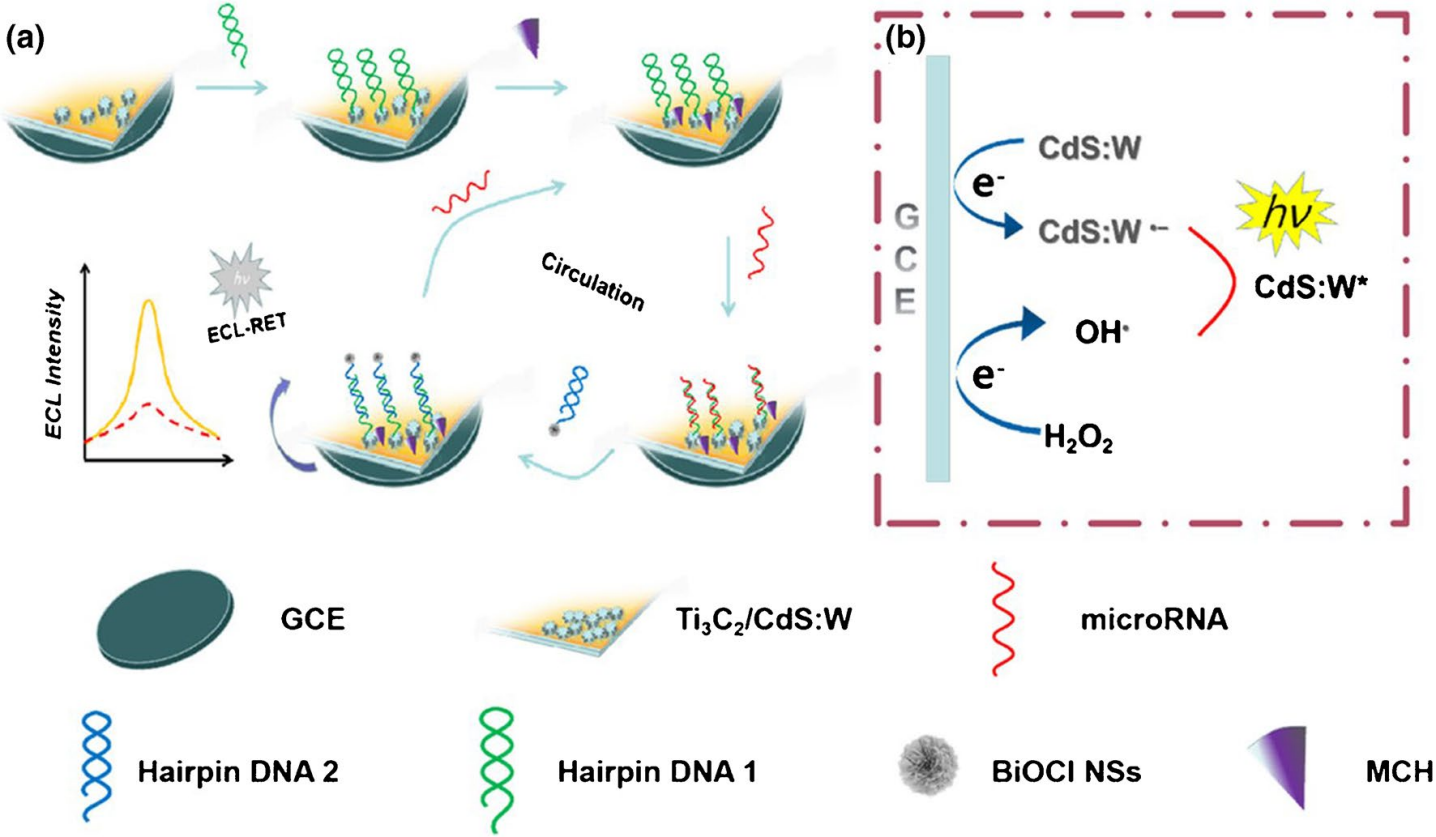
The construction process for the biosensor (**a**) electrochemiluminescent signal generation within the nanocomposite with a co-reactant H_2_O_2_ **(b)**. Reproduced with permission from ref. [218]. Copyright 2022 Springer

The performance of the electrochemiluminescent biosensor toward miRNA-141 detection was enhanced through Ti_2_C_3_ MXene-based hybrid nanocomposite [218]. The nanocomposite exhibiting UV absorption was utilized as the resonance energy transfer acceptor (Fig. 15). The miRNA-141 could be detected in the range from 0.6 pM to 4000 pM with LOD of 0.26 pM [218].

**Fig. 16:**
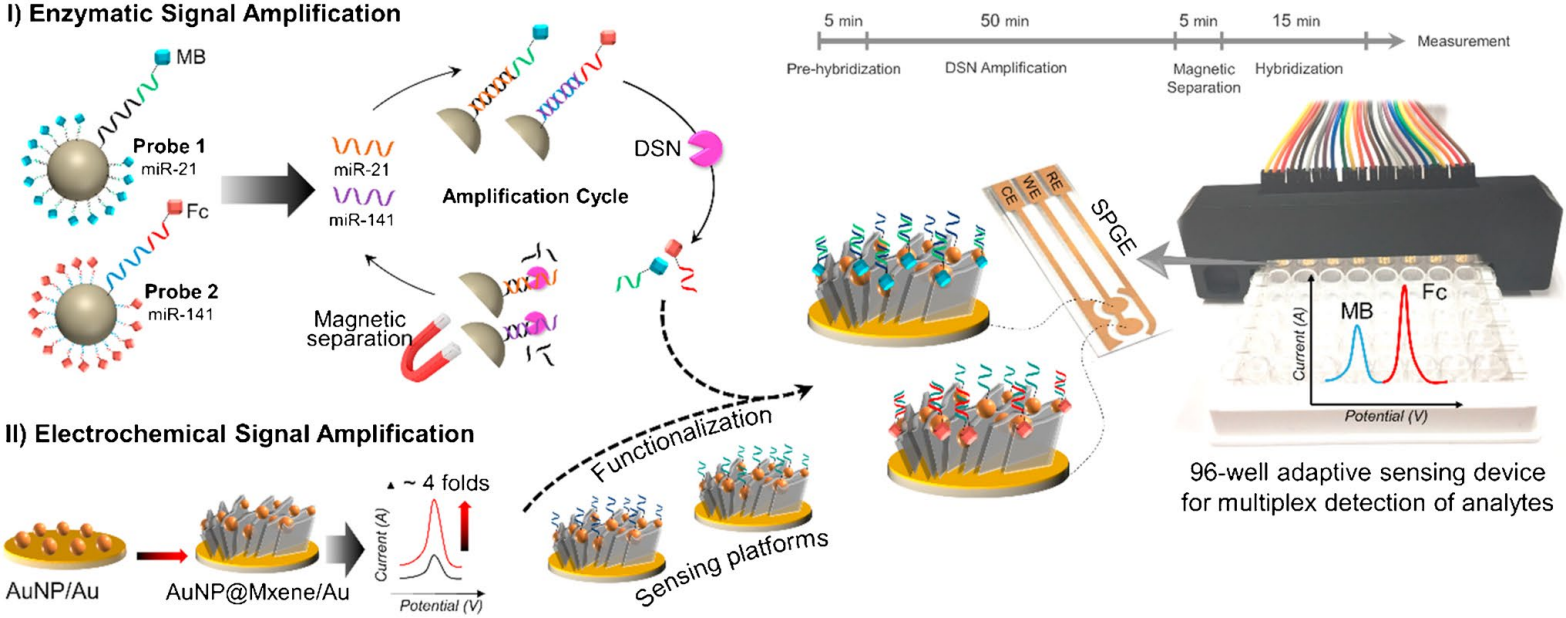
Schematic diagram representing the whole assay procedure for multiplex detection of miR-21 and miR-141. Reproduced with permission from ref. [219]. Copyright 2020 Elsevier

Mohammadniaei and colleagues combined MXene-based electrochemical signal amplification and a duplex-specific nuclease-based amplification system for rapid, attomolar, and concurrent quantification of multiple microRNAs on a single platform in total plasma (Fig. 16) [219]. Presence of MXene provided biofouling resistance and enhanced the electrochemical signals by almost fourfold of magnitude, attributed to its surface area and remarkable charge mobility. This synergetic strategy reduced the assay time to 80 min and provided multiplexing, antifouling activity, substantial sensitivity, and specificity (single mutation recognition). The LOD for the proposed biosensor for microRNA-21 and microRNA-141 was 204 aM and 138 aM, respectively, and able to detect analytes up to 50 nM [219].

Meng et al. patterned the surface area of the indium tin oxide electrode with ZnSe nanodisks:Ti_3_C_2_ MXene complex to detect the non-small-cell cancer biomarker ctDNA KRAS G12D with LOD of 0.2 fM within the linear range of 0.5 ~ 100 fM [220].

**Fig. 17:**
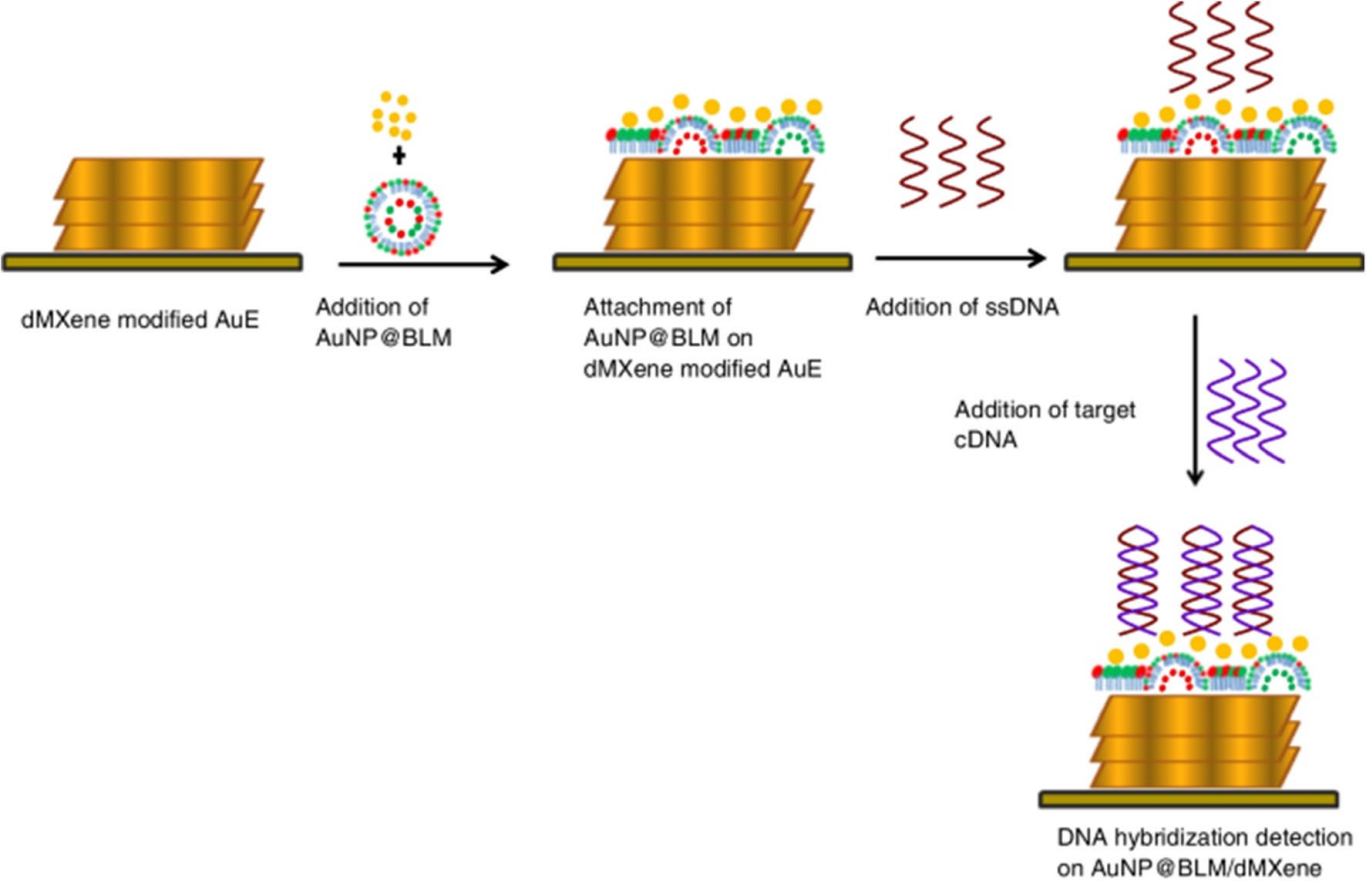
Schematic representation of the constructed biosensor for BRCA1 gene hybridization detection for breast cancer prognosis and diagnosis. Reproduced with permission from ref. [215]. Copyright 2023 Springer

Divya et al. introduced a 2D MXene nanosheet–anchored gold nanoparticle-decorated biomimetic bilayer lipid membrane (AuNP@BLM) biosensor for the attachment of thiolated single-stranded DNA (HS-ssDNA) targeting hybridization detection of BRCA1 biomarker (Fig. 17) [215]. The developed biosensor confirmed hybridization signals only to the complementary DNA (cDNA) sequence with LOD of 1zM in a linear range of 10 zM–1 μM. Moreover, a good specificity of biosensor was proved using non-complementary (ncDNA) and double-base mismatch oligonucleotide DNA (dmmDNA) sequences [215].

#### Proteins

GCE modified by MXene Ti_3_C_2_T_*x*_ interface was further patterned with a mixed zwitterionic carboxy and sulfobetaine layer deposited on the surface by an electrochemical trigger with subsequent covalent immobilization of anti-CA15-3 antibody as a bioreceptive probe for detection of a breast cancer biomarker [221]. CA 15–3, a candidate breast cancer biomarker with a molecular weight of 290–400 kDa, occurs normally at level of 3–30 U mL^−1^ in serum [222]. The designed immunosensor was able to detect glycoprotein-based CA 15–3 biomarker in a clinically relevant concentration window of up to 50 U mL^−1^ [221]. Moreover, it was confirmed, that Ru(NH_3_)_6_Cl_3_ redox probe has a potential to be applied for better understanding of interfacial properties onto the proteins modifying electrode surfaces [221].

**Fig. 18:**
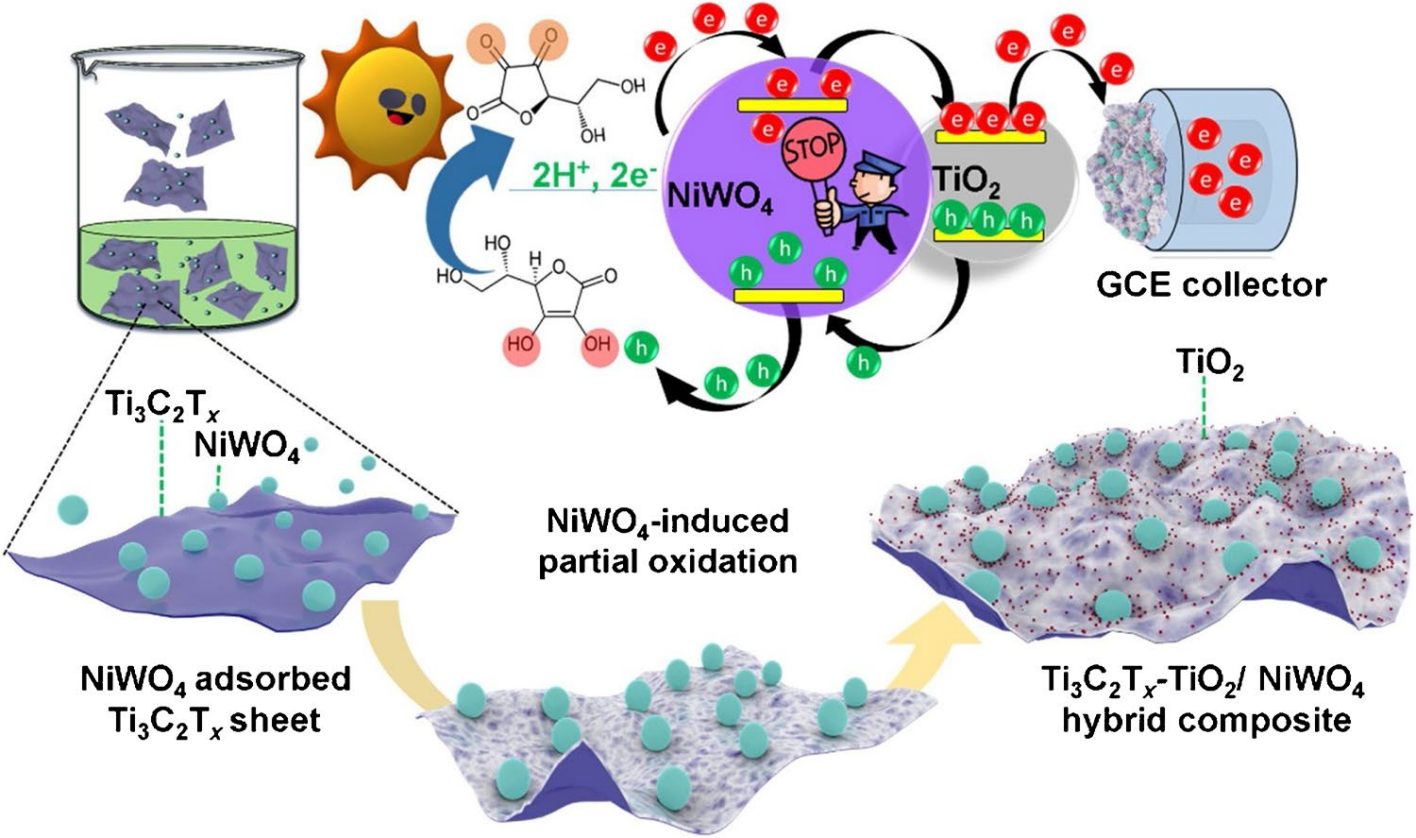
Surface adsorption of NiWO_4_ NPs over ultra-thin Ti_3_C_2_T_*x*_ sheets in solution, with surface-bound interactions leading to surface fracturing and, ultimately, partial surface oxidation of Ti_3_C_2_T_*x*_, realizing in situ TiO_2_ formation in MX-NiWO_4_. Corresponding heterojunction shows efficient charge-carrier transfer at the in situ engineered interface during photo-catalytic oxidation of mediator. Reproduced with permission from ref. [223]. Copyright 2021 Elsevier

Soomro with co-workers applied photo-active NiWO_4_ NPs to induce partial surface oxidation of Ti_3_C_2_T_*x*_, sheets resulting in the formation of a hybrid composite (Fig. 18) [223]. The developed biosensor with photo-electrochemical characteristics of the hybrid composite was able to detect prostate specific antigen with LOD of 0.15 fg mL^−1^ in a wide concentration range from 1.2 fg mL^−1^ to 0.18 mg mL^−1^ [223].

The nanocomposite of MXene loaded with AuNPs and methylene blue (MB) exhibited excellent conductivity, where the AuNPs were able to capture biomolecules containing sulfhydryl terminus, and the MB molecules were used to generate an electrochemical signal [224]. In the presence of a model target prostate specific antigen (an enzyme, i.e., protease), the recognizing sequence was recognized and cleaved, and the ratiometric signal of Fc and MB indicated the concentration of the analyte accurately with high sensitivity within a detection range from 5 pg mL^−1^ to 10 ng mL^−1^ and with LOD down to 0.83 pg mL^−1^. The electrochemical biosensor possessed high selectivity, accuracy, and sensitivity even in real complex biological samples because of the excellent antifouling ability [224].

Song et al. developed a label-free and aptamer-based sensitive assay platform detecting carcinoembryonic antigen with LOD of 0.32 fg mL^−1^ by applying the trimetallic nanoparticle-decorated MXene nanosheet–modified electrode as the catalytic interface and an exonuclease III-assisted dual-amplification strategy [225].

**Fig. 19:**
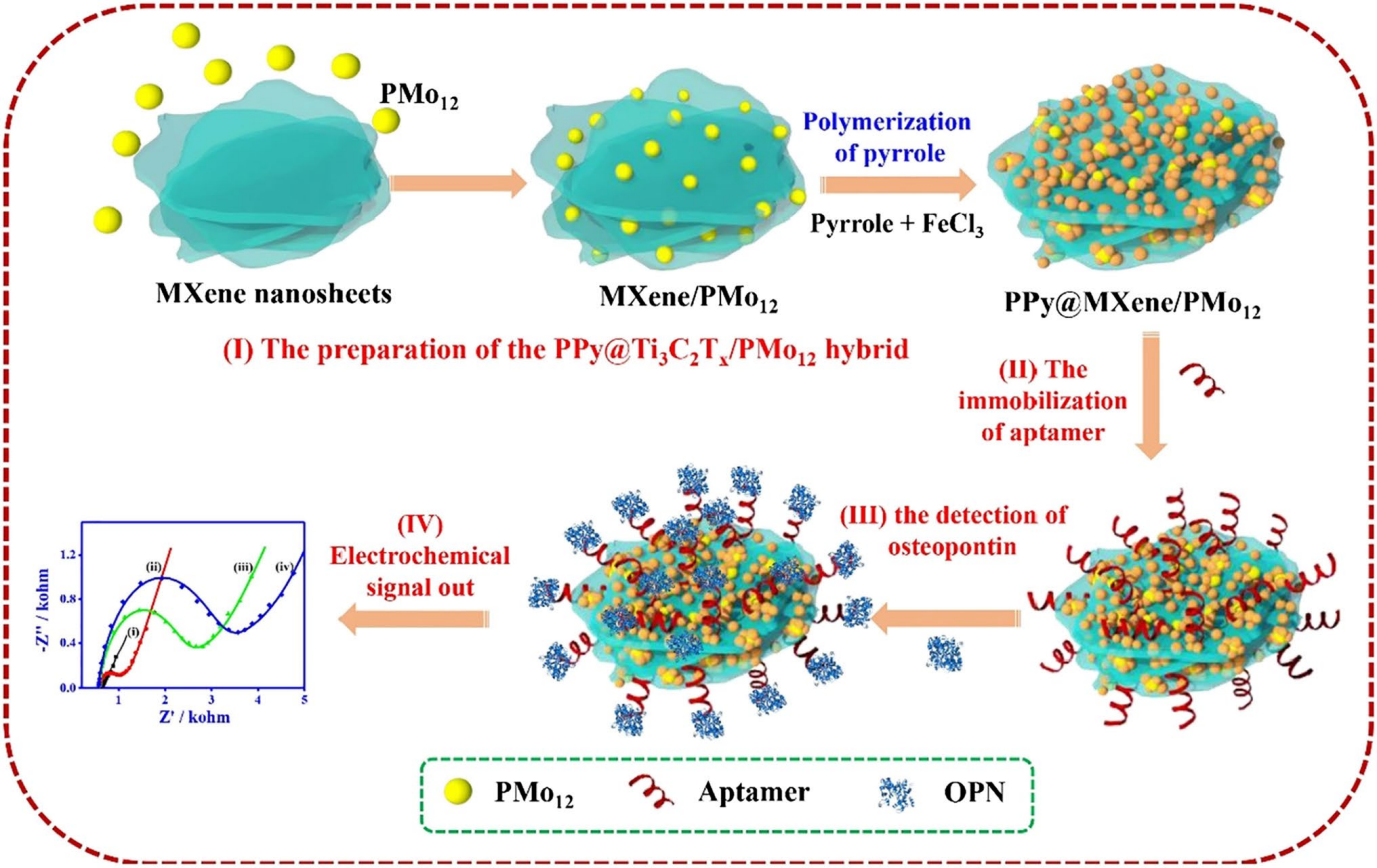
Schematic diagram of the aptasensor fabrication based on PPy@Ti_3_C_2_T_*x*_/PMO_12_ for the osteopontin detection, including (I) the preparation of the PPy@Ti_3_C_2_T_*x*_/PMo_12_ hybrid, (II) the aptamer immobilization, (III) the osteopontin detection, and (IV) the electrochemical signal reading. Reproduced with permission from ref. [226]. Copyright 2019 Elsevier

The polypyrrole-modified hybrid NP-based aptasensor (Fig. 19) could detect a phosphoprotein osteopontin associated with human cervical cancer in a sensitive way with LOD of 0.98 fg mL^−1^ within a linear concentration range of 0.05 pg mL^−1^ to 10.0 ng mL^−1^ [226].

The affinity-based biosensor (BSA/anti-CEA/f-Ti_3_C_2_-MXene/GCE) was applied for detection of carcinoembryonic antigen, a cancer biomarker related to different types of cancer diseases, with LOD of 0.000018 ng mL^−1^ within a linear concentration range of 0.0001–2000 ng mL^−1^ [227].

The amplification of the amperometric signal and transistor’s performance was performed by Xu et al. detecting survivin related to osteosarcoma, an aggressive malignant cancer affecting the health of children, adolescents, and young adults, by applying MXene/PEDOT:PSS-based organic electrochemical transistor biosensor offering LOD down to 10 pg mL^−1^ [228].

**Fig. 20:**
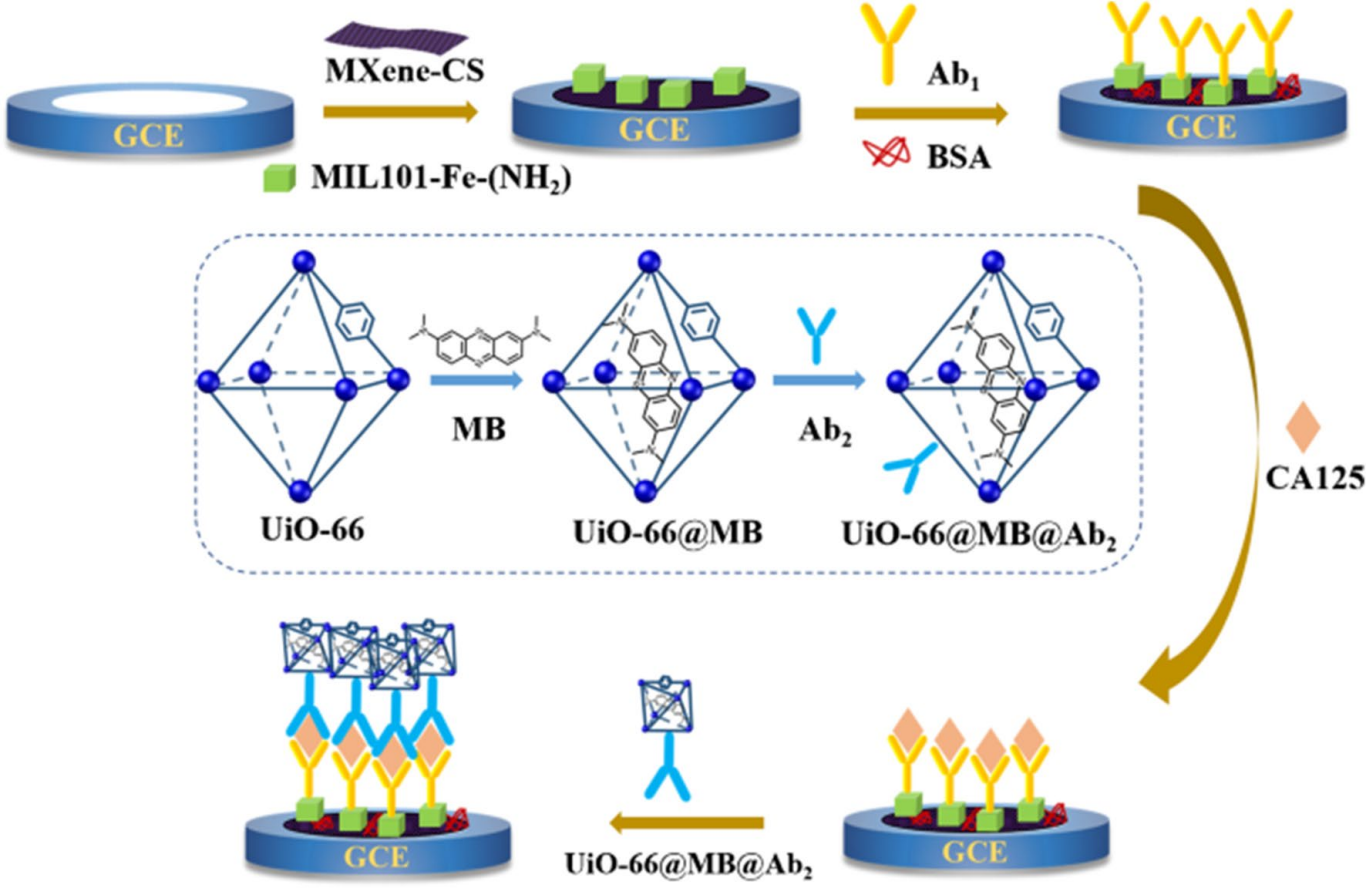
Fabrication of the device followed by detection of CA125. Reproduced with permission from ref. [80]. Copyright 2023 Springer

Qu et al. described an electrochemical immunosensor evaluating carbohydrate antigen 125 (CA125) within serum via the dual metal–organic framework (MOF) sandwich strategy [80]. The composite combined electrically conductive uniform MXene together with mesoporous and catalytically active MIL-101(Fe)-NH_2_ material containing rich amino groups to attach primary antibodies. MOF loaded with methylene blue (MB) as a signal tag increased the loading rates of the secondary antibody and generated a redox signal (Fig. 20). The LOD of 0.006 U mL^−1^ or CA125 was achieved with the proposed immunosensor [80].

**Fig. 21:**
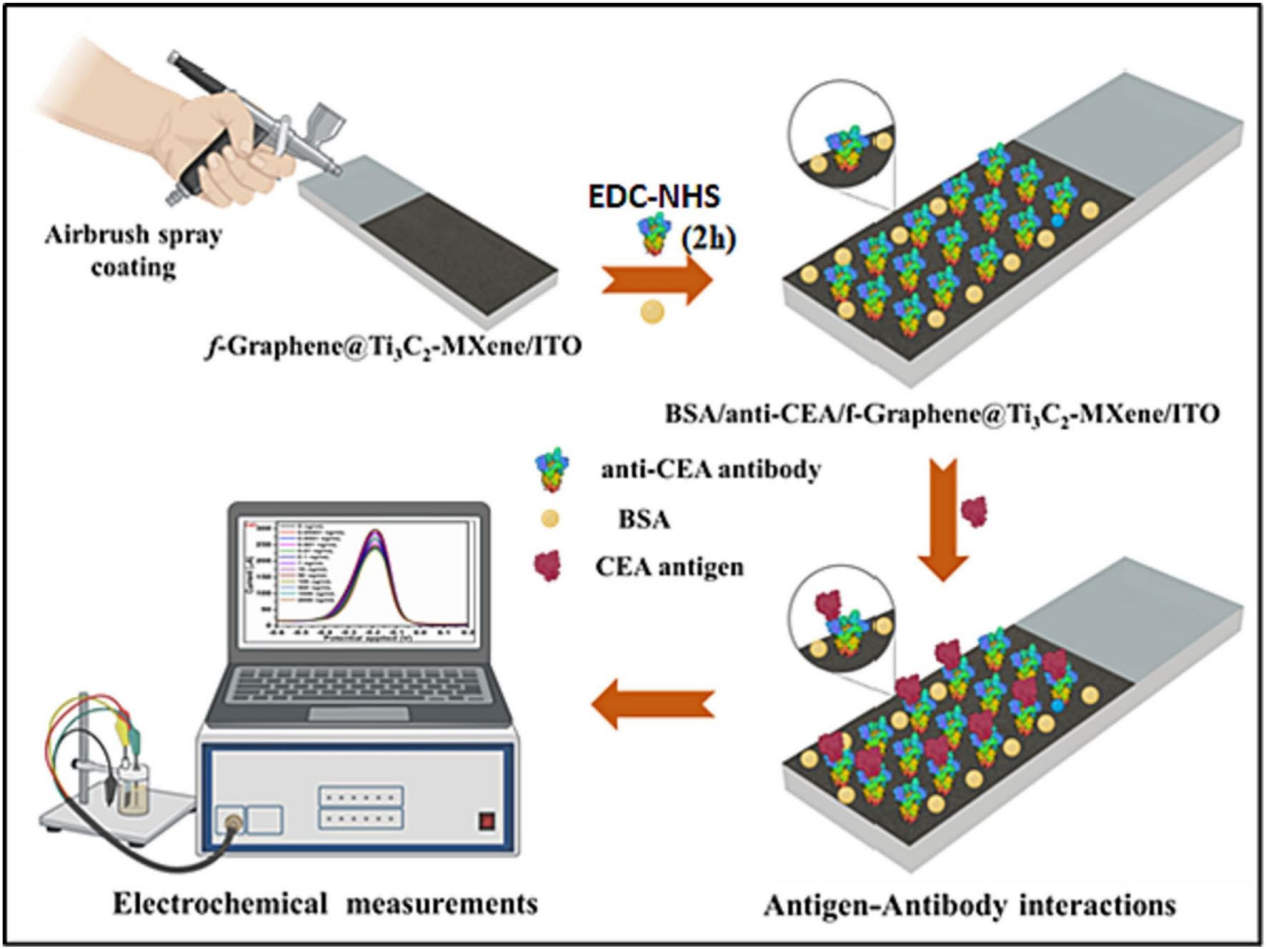
Pictorial representation and stepwise fabrication of BSA/anti-CEA/f-graphene@Ti_3_C_2_-MXene/ITO immunoelectrode for electrochemical detection of CEA biomarker. Reproduced with permission from ref. [229]. Copyright 2023 Elsevier

Kalkal et al. employed the air-brush spray coating technique to deposit the uniform thin films of amine functionalized graphene (f-graphene) and Ti_3_C_2_-MXene nanohybrid on ITO-coated glass substrate for efficient carcinoembryonic antigen (CEA) detection [229]. The monoclonal anti-CEA antibodies were attached onto the deposited thin films through the EDCNHS chemistry and further the non-specific binding sites were blocked with BSA (Fig. 21). An electrochemical BSA/anti-CEA/*f*-graphene@Ti_3_C_2_-MXene/ITO immunoelectrode was able to detect CEA biomarker with LOD of 0.30 pg mL^−1^ and a sensitivity of 28.88 μA [log (pg mL^−1^)]^−1^ cm^−2^ in a linear range from 0.01 pg mL^−1^ to 2000 ng mL^−1^ [229].

### Analysis of cells/exosomes/viruses

**Fig. 22:**
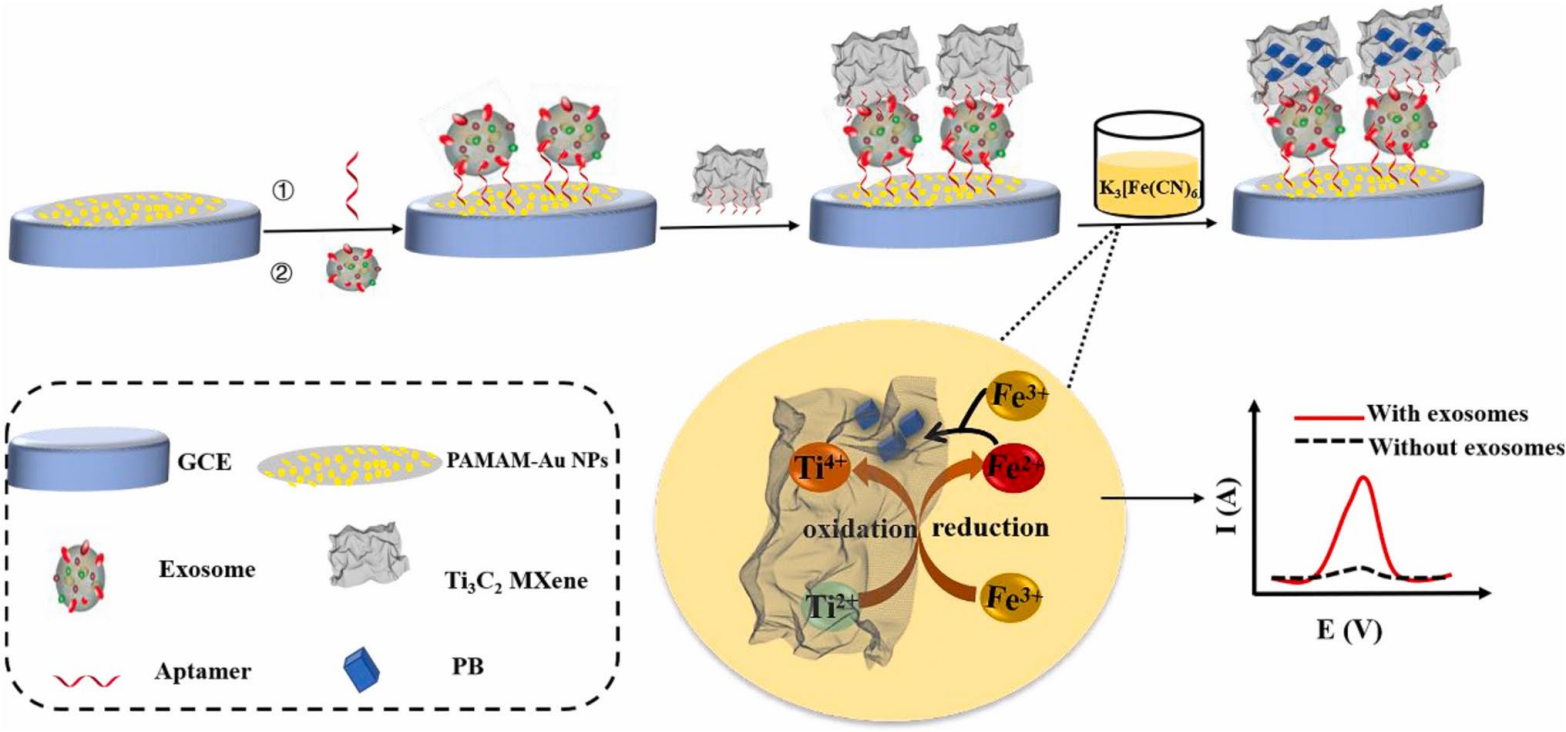
The principle of the electrochemical biosensor for exosomes activity detection using a signal amplification strategy. Reproduced with permission from ref. [230]. Copyright 2021 Elsevier

Exosomes as the novel carrier of potential cancer biomarkers were analyzed by Zhang et al. with electrochemical hybrid nanoprobe prepared by in situ generated Prussian Blue on the surface of Ti_3_C_2_ MXene [230]. A CD63 aptamer-modified poly(amidoamine) (PAMAM)-AuNP electrode interface can specifically interact with the CD63 protein on the exosomes derived from OVCAR cells (Fig. 22). The achieved LOD was 229 particles μL^−1^ and exosomes could be determined in a wide a linear range from 5 × 10^2^ particles μL^−1^ to 5 × 10^5^ particles μL^−1^ [230]. MXene-based nanoplatforms capable of in vitro detection of tumor markers such as exosomes and CEA have been successfully verified [231].

**Fig. 23:**
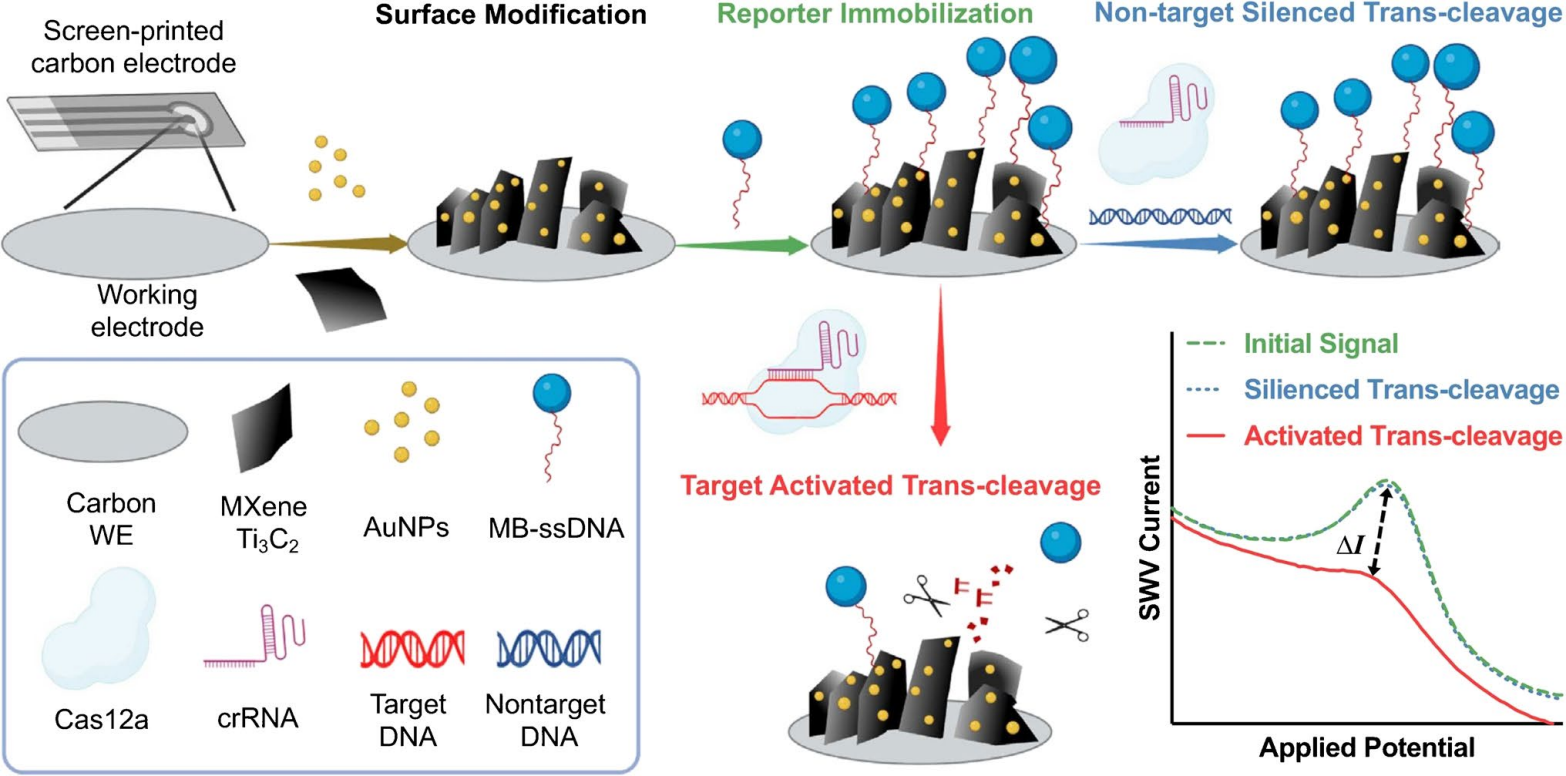
Schematic illustration of AuNPs/MXene Ti_3_C_2_-assisted biosensor for viral DNA detection. Reproduced with permission from ref. [76]. Copyright 2022 American Chemical Society

Duan with co-workers demonstrated AuNPs/MXene Ti_3_C_2_-based clustered regularly interspaced short palindromic repeats powered electrochemical sensor for detection of human papillomavirus 18 (HPV-18) DNA (Fig. 23) with LOD of 1.95 pM in a linear concentration range from 10 pM to 500 nM [232]

Wang together with colleagues produced an electrochemical luminescence biosensor based on Ti_3_C_2_T_*x*_/ZIF-8 nanocomposite as an emitter to determine human immunodeficiency virus (HIV-1 protein) causing acquired immune deficiency syndrome (AIDS) with LOD of 0.3 fM in the linear range from 1 fM to 1 nM. In this approach, K_2_S_2_O_8_ as the co-reactant and conductive carbon black combined with magnetic nanoparticles as the quenching agent were employed [233].

**Fig. 24:**
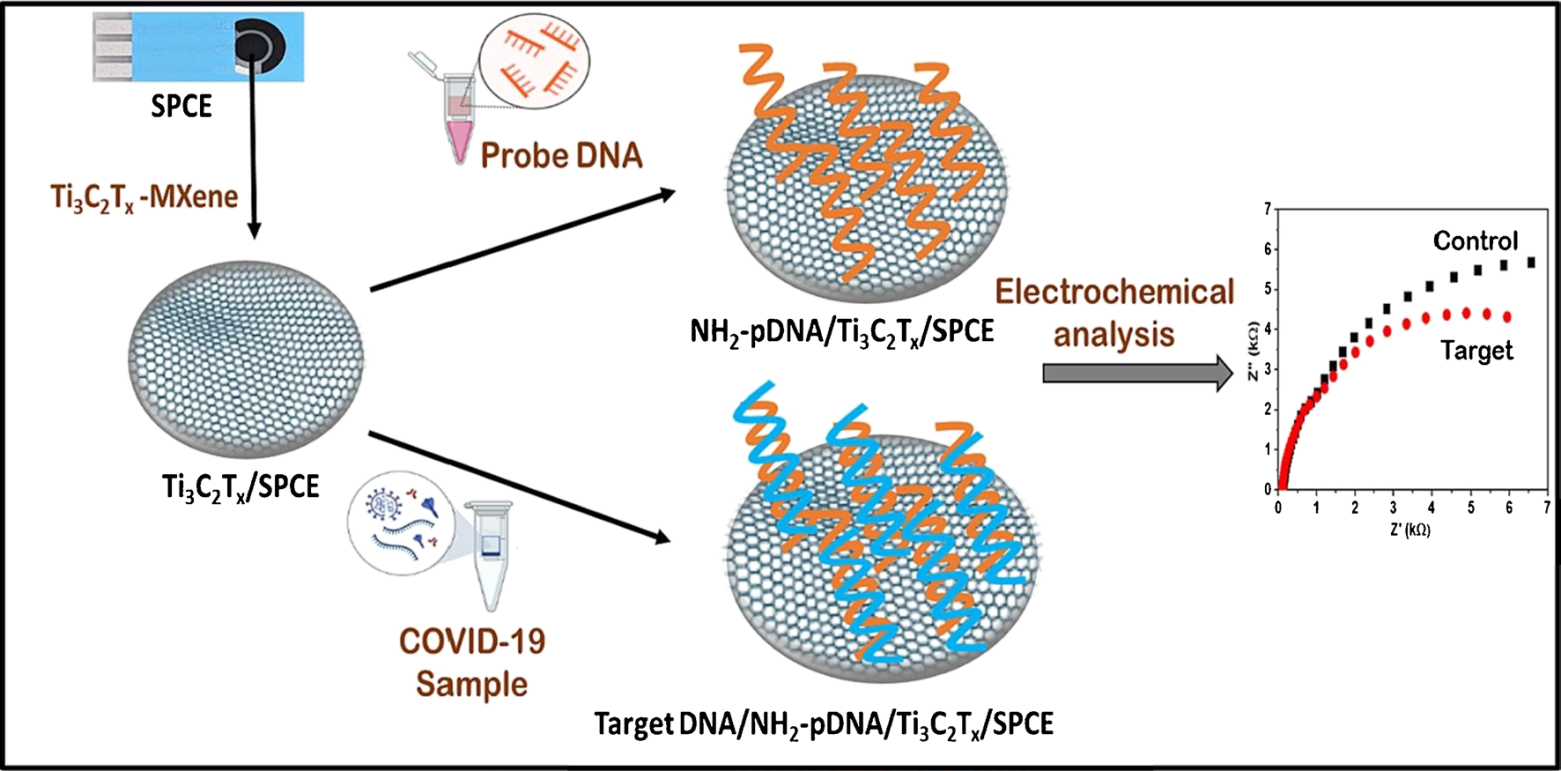
Application of screen printed carbon electrodes for detection of SARS-CoV-2 using impedimetric assays. Reproduced with permission from ref. [83]. Copyright 2023 Elsevier

Bharti et al. utilized a disposable screen printed carbon electrode (SPCE) modified with Ti_3_C_2_T_*x*_ MXene nanosheets followed by amino-functionalized probe DNA (NH_2_-pDNA) as a robust surface for the sensing of SARS-CoV-2 (Fig. 24) [83]. The NH_2_-pDNA/Ti_3_C_2_T_*x*_/SPCE bioelectrode determined SARS-CoV-2 by applying electrochemical impedance spectroscopy method within target DNA concentration of 0.1 pM–1 μM and with LOD of 0.004 pM. Moreover, LOD of 0.003 pM was obtained for SARS-CoV-2 target in a spiked serum sample. The shelf life up to 40 days at storage temperature of 4 °C was observed [83].

Liu et al. utilized 2D bimetallic CoCu–zeolite imidazole framework and zero-dimensional Ti_3_C_2_T_*x*_ MXene-derived carbon dots to prepare a suitable interface for anchoring B16-F10 cell–targeted aptamer strands. The cytosensor could detect B16-F10 cells in a concentration range of 1 × 10^2^–1 × 10^5^ cells mL^−1^ with LOD of 33 cells mL^−1^ [234].

In an effort to improve antifouling and biocompatible properties of electrochemically active surface, Lian et al. developed a sandwich-type immunoassay utilizing platelet membrane/Au nanoparticle/delaminated V_2_C nanosheets as the sensing electrode interface and methylene blue/aminated metal organic framework as an electrochemical signal probe. The LOD for CD44-positive cancer cell in complex liquids reached 1.4 pg mL^−1^ in a linear range from 0.5 to 500 ng mL^−1^ [235].

### Different wearable sensors

Advances in wearable sensors with their ability to sense various body parameters precisely have helped in accelerating the personalized healthcare revolution. Sensing materials for wearable applications, in general, are expected to be flexible, biocompatible, electrically conducting, electrochemically active, and of low cost. The discovery of MXenes has opened up new prospects in wearable sensing as most MXenes are predicted to have metallic conductivity, while a few combinations exhibit semiconductor behavior. Importantly, the surface functional groups are strongly coupled to the electronic properties of MXene. Moreover, the structural defects and mixed surface groups introduced during the synthesis of MXene influence its electrical conductivity. The etching process and intercalation method can also have an impact on the conductivity of MXene as intercalation of the Li^+^ cation results in better conductivity than organic intercalation. The high electrical conductivity of MXene with controlled alignment of 2D sheets enables the piezoresistive sensing mechanism suitable for wearable sensing applications [236].

There is an increased demand for flexible, soft, highly efficient and high-performance sensing devices [237, 238]. Specifically, stretchable, wearable, and highly sensitive or responsive strain sensors have gained enormous research interest owing to their potential applications in soft robotics, monitoring human health, monitoring human activity, and human–machine interfacing. Generally, flexible wearable sensors encompass piezoelectric, piezoresistive, capacitive, and triboelectric sensors. Piezoresistive sensors transduce applied pressure into a resistance signal and are thus ideally suited for portable healthcare monitoring. Ti_3_C_2_-MXene-based sensors were applied to monitor joint bending, swallowing, and coughing, for the recognition of various human activities (to monitor the subtle movement caused by microexpression) such as eye blinking, cheek bulging, and throat swallowing as well as variation in the current for the bending-releasing activity of the elbow, fingers, and ankle. The corresponding sensor was attached in series to a microcircuit embedded with a Bluetooth system for transforming various current or resistance variations into wireless electromagnetic wave signals. MXenes and graphene-based wearable biochemical sensors were applied in a number of areas including but not limited to electrolyte monitoring, glucose monitoring, micro/macromolecular organics metabolite, volatile gases monitoring, and humidity sensing [239].

Ti_3_C_2_ MXene-cotton textile-based flexible piezoresistive pressure sensor has been demonstrated by a simple and low-cost dip-coating method [240]. The as-fabricated highly flexible sensors were attached to the radial artery of the wrist using a scotch tape. It exhibited high sensitivity with a rapid response time (26 ms) and exceptional cyclic stability for 5600 cycles. The sensor was utilized for real time monitoring of human physiological signals namely wrist pulse, voice detection, and finger motions [240].

**Fig. 25:**
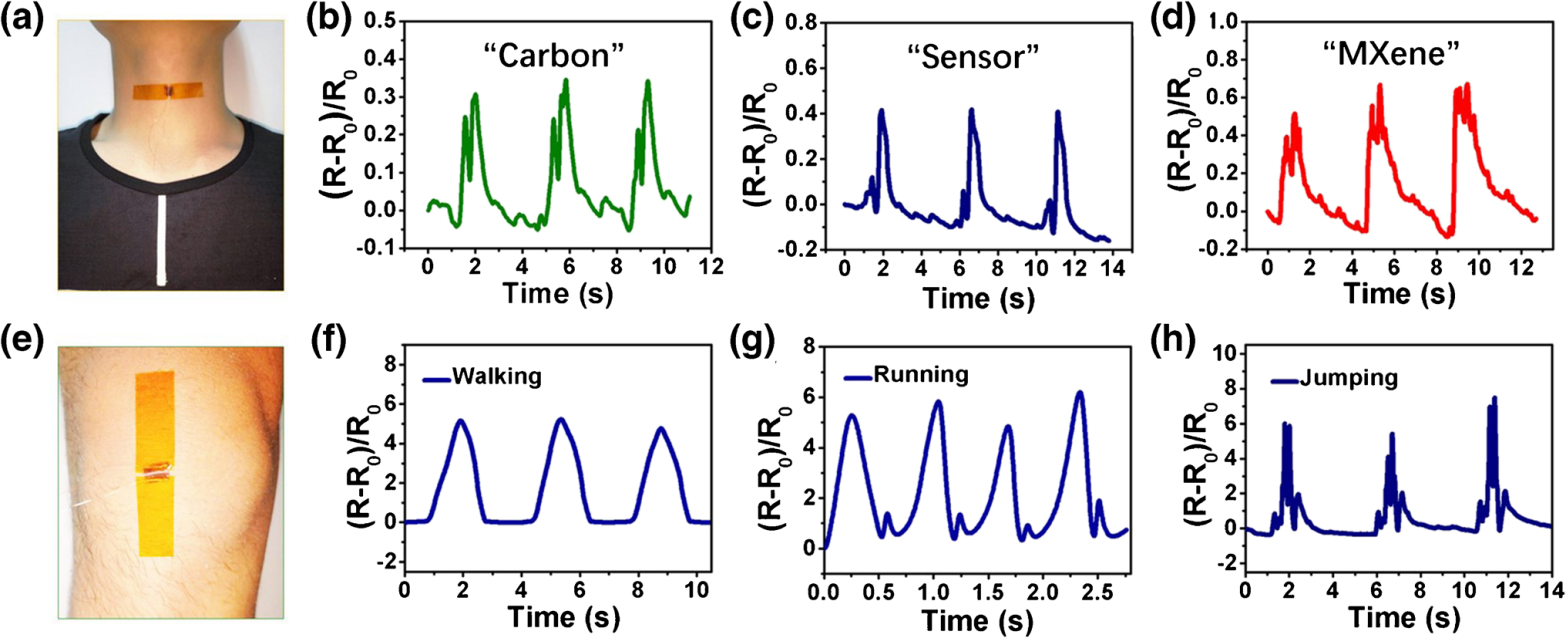
**a** Ti_3_C_2_T_*x*_ MXene/CNT strain sensor attached to a person throat; **b**–**d** response curves obtained when individual spoke “carbon,” “sensor,” and “MXene”; **e** sensor attached to the human knee; **f**–**h** resistance responses of the sensor in detecting human leg motion: walking, running, and jumping. Reproduced with permission from ref. [241]. Copyright 2018 American Chemical Society

In another instance, a percolative network consisting of Ti_3_C_2_T_*x*_ MXene/carbon nanotube (CNT) composites resulted into a versatile strain sensor (Fig. 25) [241]. A layer-by-layer spray coating technique was applied delivering an ultrathin device (device dimension < 2 mm) exhibited extremely low LOD of 0.1% strain, high sensitivity, and tunable sensing range (30–130% strain). The exceptional sensing performance allowed successful detection of both small deformations such as phonation as well as large motions such as walking, running, and jumping. Voice recognition ability of this sensor makes it potential material for voice recuperation and human–machine interfacing [241].

Another example is Ti_3_C_2_T_*x*_-based wearable electrochemical impedimetric immunosensor with a 3-D electrode network for non-invasive cortisol biomarker identification in human sweat [242]. Laser-induced graphene was the basic material used for construction of the electrode since it is stable and had good electrical properties. The cortisol sensor had a very low LOD of 3.88 pM and excellent selectivity [242].

A sensitive dopamine sensor was created using a bionanocomposite with MXene nanoparticles serving as a conductive matrix for attachment of Pd/Pt NPs [243]. The hydrophobic aromatic group adsorbed on the surface of MXenes induces the in situ growth of PdNPs and Pd/Pt NPs. The sensor showed excellent linearity for detection of dopamine in the concentration range of 0.2–1000 μM, as well as high selectivity against ascorbic acid, glucose, and uric acid [243].

*Pressure/strain sensors*

**Fig. 26:**
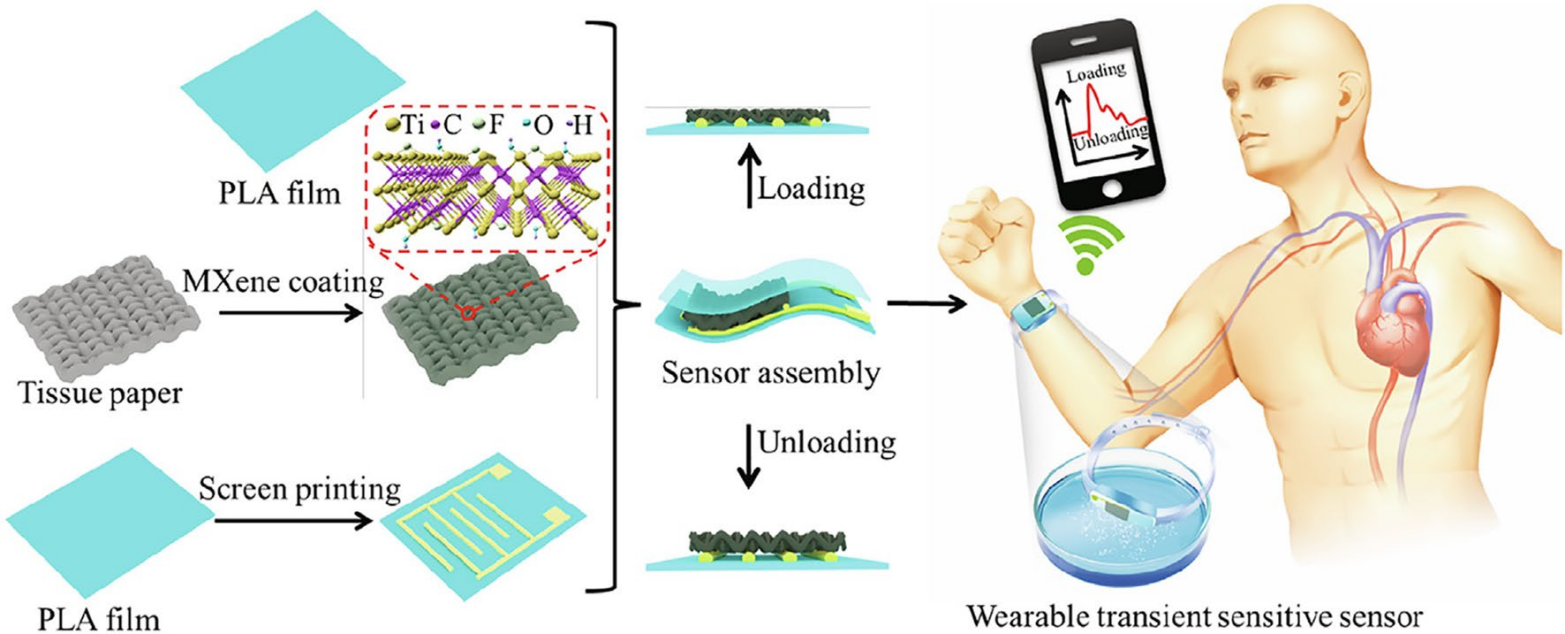
Schematic representation of the procedure to fabricate MXene nanosheet-based flexible wearable transient pressure sensors. Reproduced with permission from ref. [244]. Copyright 2019 American Chemical Society

In order to detect transient changes in pressure, a flexible, highly sensitive, and degradable wearable sensor based on Ti_3_C_2_T_*x*_ Mxene nanosheets was developed impregnated with tissue paper sandwiched between a polylactic acid sheet and an interdigitated conducting electrode coated polylactic acid sheet (Fig. 26) [244]. The as-fabricated flexible pressure sensor demonstrated high sensitivity with low LOD (10.2 Pa), wide range up to 30 kPa, fast response (11 ms), excellent reproducibility (over 10,000 cycles), low consumption of energy (10-8 W), and good degradability [244].

A newly developed microchannel restricted Ti_3_C_2_T_*x*_ MXene-derived flexible piezoresistive sensor allowed simultaneous sensing of pressure, sound, and acceleration [245]. It exhibited high sensitivity (99.5 kPa^−1^), a low LOD (9 Pa), fast response (4 ms), and exceptional durability (over 10,000 cycles). The flexible piezoresistive sensor was attached to the throat and wrist pulse for human activity monitoring. The sensor was able to record the current variations upon speaking different words and hence capable to recognize the signals of weak throat vibrations [245].

A flexible piezoresistive pressure sensor was derived from polyurethane and chitosan sponge coated with Ti_3_C_2_T_*x*_ sheet sensor providing a versatile sensing platform for monitoring small as well as large pressure signals [246]. The sensor exhibited highly compressible and stable piezoresistive response for the compressive strains up to 85% and a stress of 245.7 kPa and a reproducibility for around 5,000 loading–unloading cycles with a response time of 19 ms. The sensor was used for monitoring human physiological signals and the movements of insects as well as for detecting human voices and breaths in a non-contact mode [246].

**Fig. 27:**
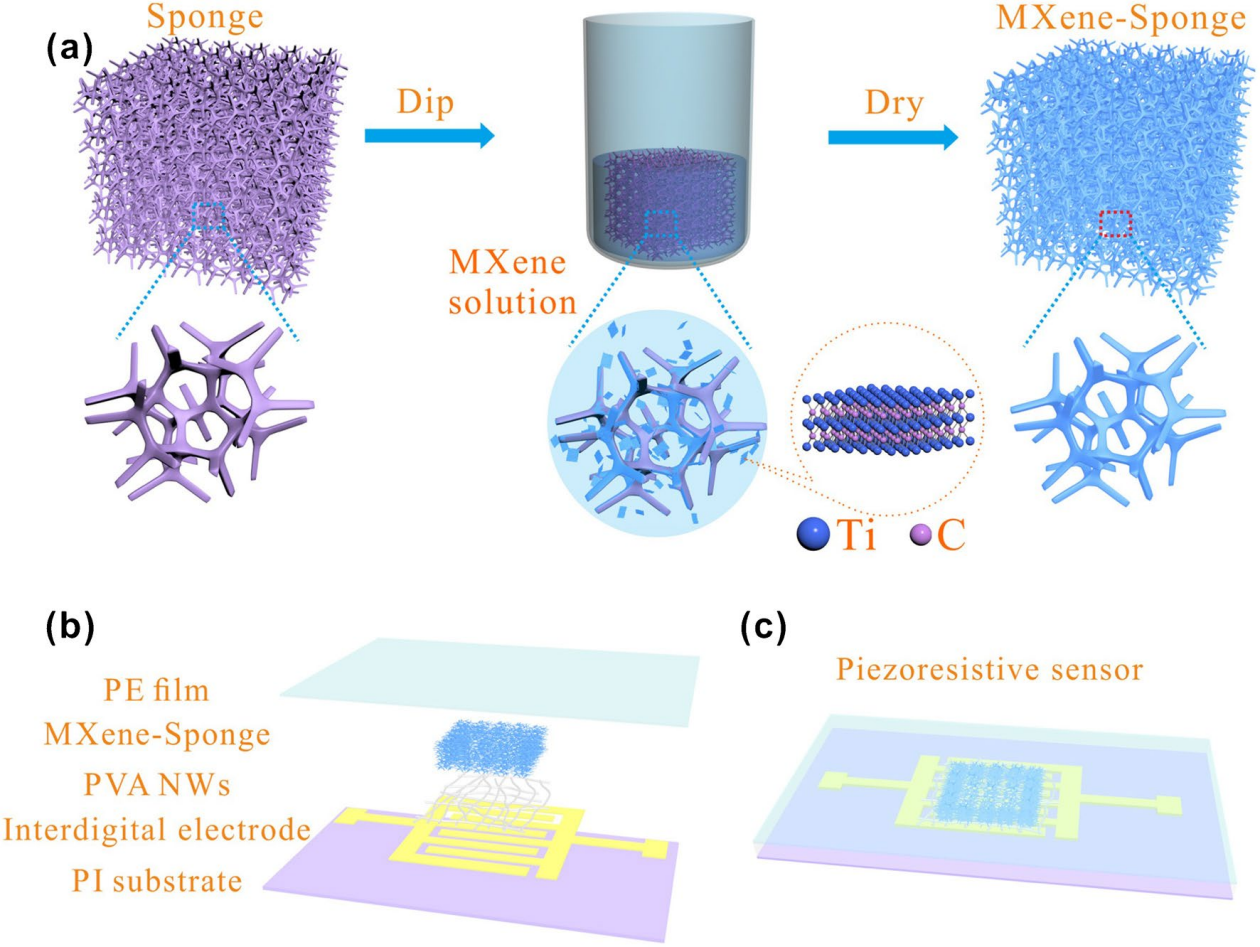
Schematic representation of **a** Ti_3_C_2_T_*x*_ MXene sponge fabrication, **b**, **c** construction of Ti_3_C_2_T_*x*_ MXene sponge/PVA NW-derived sensor. Reproduced with permission from ref. [247]. Copyright 2018 Elsevier

In yet another example, a 3D hybrid Ti_3_C_2_T_*x*_ MXene–based sponge network with porous structure was applied as a piezoresistive sensor [247]. The Ti_3_C_2_T_*x*_-based sponge was prepared by a facile and efficient dip-coating technique where semiconducting polyvinylalcohol nanowires were used as a spacer (Fig. 27). It exhibited excellent sensitivity over a broad range of pressure, a low LOD of 9 Pa, and a rapid response time of 138 ms with exceptional durability over 10,000 cycles. This Ti_3_C_2_T_*x*_ MXene sponge/PVA NW-derived sensor exhibited the higher sensitivity in comparison with the Ti_3_C_2_T_*x*_ MXene sponge sensor, additionally showing rapid response and recovery times of 138 ms and 127 ms, respectively. The sponge-sensor was further utilized for real-time monitoring of small strain, human physiological behavior, and the change in the balloon size. Specifically, characteristic peaks corresponding to three waveforms related to percussion, tidal, and diastolic can be seen which indicates excellent sensitivity of the sensor [247].

A highly sensitive piezoresistive sensor was demonstrated based on Ti_3_C_2_ MXene with bioinspired micro spine-like structure formed by a facile abrasive paper stencil printing method [248]. It exhibited high sensitivity (151.4 kPa^−1^), short response time (< 130 ms), very low LOD of 4.4 Pa, and exceptional cyclic stability (over 10,000 cycles). Besides, the fabricated piezoresistive sensor demonstrated excellent performance toward detection of physiological signals and quantitatively monitoring pressure distributions as well as remote and real-time monitoring of the motion of an intelligent robot [248].

It has been shown that compressible and elastic carbon aerogels derived from Ti_3_C_2_ MXene and cellulose nanocrystals can be applied as wearable piezoresistive sensors [249]. Cellulose nanocrystals were employed as a dispersant and nano-support to attach Ti_3_C_2_ nanosheets into a lamellar carbon aerogel with improved mechanical strength. The interaction between Ti_3_C_2_ MXene and cellulose nanocrystals resulted in a continuous wave-shaped lamellar structure which can withstand exceedingly high compression strain (95%) and long-lasting compression (10,000 cycles) at 50% strain. The aerogel sensor exhibited ultrahigh linear sensitivity in low pressure (114.6 kPa^−1^) as well as high pressure (45.5 kPa^−1^) regions with a very low LOD of pressure change detection with reproducibility for more than 2,000 cycles. All these superior characteristics of the carbon aerogel make it a prosperous material for wearable piezoresistive devices as pressure or strain sensors [249].

Another strain sensor was derived from a unique hybrid network of Ti_3_C_2_T_*x*_ MXene NPs and nanosheets [250]. The synergistic movement of NPs and nanosheets confers the hybrid network with excellent electrical and mechanical properties. The fabricated strain sensor exhibited excellent sensitivity over a broad stretching range (0–53%), extremely low LOD (0.025%), and excellent recycling durability (over 5,000 cycles). Such kind of performance renders the strain sensors capable of detecting full range of human movements [250].

**Fig. 28:**
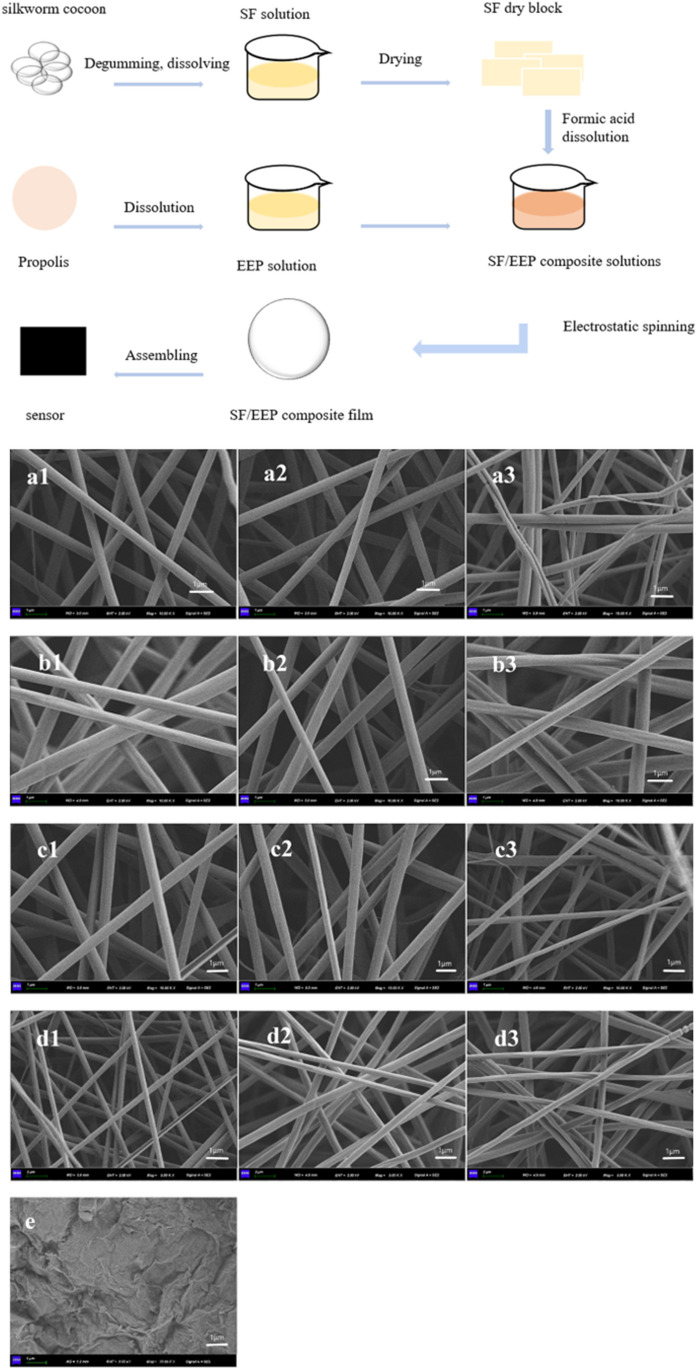
Flow chart of sensor preparation (upper image). SEM images of SF composite films. (**a**1–**a**3) Pure silk fibroin film with a concentration of 18wt%, 20wt%, and 22wt% under magnification of 10 k. (**b**1–**b**3) Under magnification of 10 k, silk fibroin concentration was 20%, propolis concentrations were 0.5wt%, 1wt%, and 2wt% of the composite films, respectively. (**c**1–**c**3) Under magnification of 10 k, composite membrane with silk fibroin concentration of 20wt%, propolis concentration of 1wt%, and voltage of 16 kV, 18 kV, and 20 kV, respectively. (**d**1–**d**3) Under magnification of 5 k, the silk fibroin concentration was 20wt%, the propolis concentration was 1wt%, the voltage was 18 kV, and the injection speed were 0.004 ml min^−1^, 0.006 ml min^−1^, and 0.008 ml min^−1^ of the composite membranes, respectively. (**e**) Under magnification of 10 k, SEM image of the composite membrane (lower image). Reproduced with permission from ref. [251]. Copyright 2023 Springer

Fan et al. came up with a biocompatible, breathable, and highly sensitive silk fibroin (SF)/propolis (EEP)/graphene(GR)/MXene nanocomposite-based flexible wearable sensor with antibacterial properties due to the inclusion of propolis [251]. Graphene and MXene dispersions were step by step sprayed onto nanocomposite fiber membranes (Fig. 28). The developed sensor exhibited a wide sensing range of 1–50 kPa, repeatability of 100 cycles and high sensitivity of 3 kPa^−1^. The movements of finger, wrist, elbow, and knee joints could be monitored with this sensor [251].

**Fig. 29:**
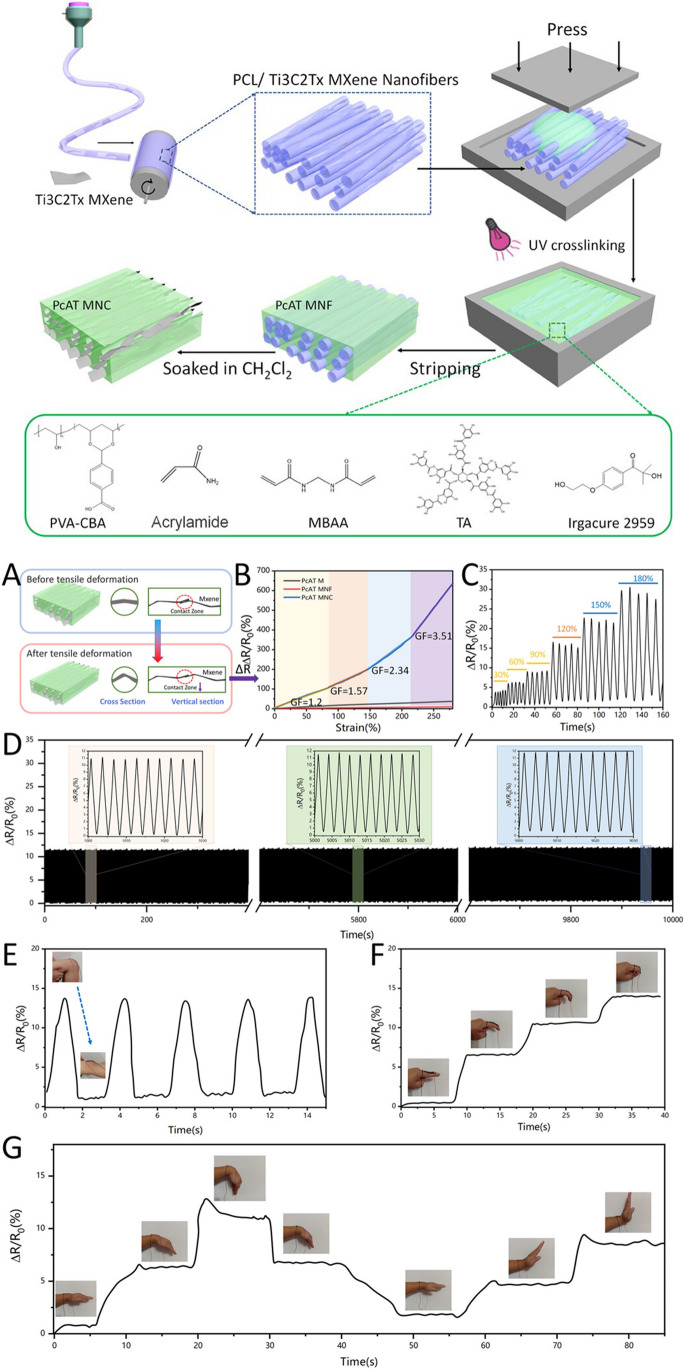
Graphic Illustration of the preparation nanofibers by combining electrospinning and the template method (upper image). Strain sensing ability of the device.) Expected strain sensing mechanism of device; **B** the sensitivity of the strain sensor at 0–280% strain; **C** resistance changes of the strain sensor under 30–180% strains; **D** resistance changes under cyclic tests (3,000 times); and resistance changes of the sensor during monitoring different human activities including **E** knee bending, **F** finger bending, and **G** wrist bending (lower image). Reproduced with permission from ref. [252]. Copyright 2023 American Chemical Society

Gong et al. fabricated a novel type of Ti_3_C_2_T_*x*_ MXene–based nanochannel hydrogel sensor taking advantage of the unique structure of electrospun fiber textile and the properties of the double network hydrogel [252]. The nanofibers were synthesized through electrostatic spinning, and then the nanochannels within the device were formed. In the cavity of the nanochannels, the Ti_3_C_2_T_*x*_ MXene nanosheets had more space for moving in response to varying degrees of deformation, which enhanced the sensor’s sensitivity. In an effort to improve the self-adhesion properties of wearable sensors, tannin (TA) was added to the hydrogel system (Fig. 29). The hydrogel sensor successfully detects different human motions and physiological signals (e.g., low pulse signals) with high stability and sensitivity [252].

**Fig. 30:**
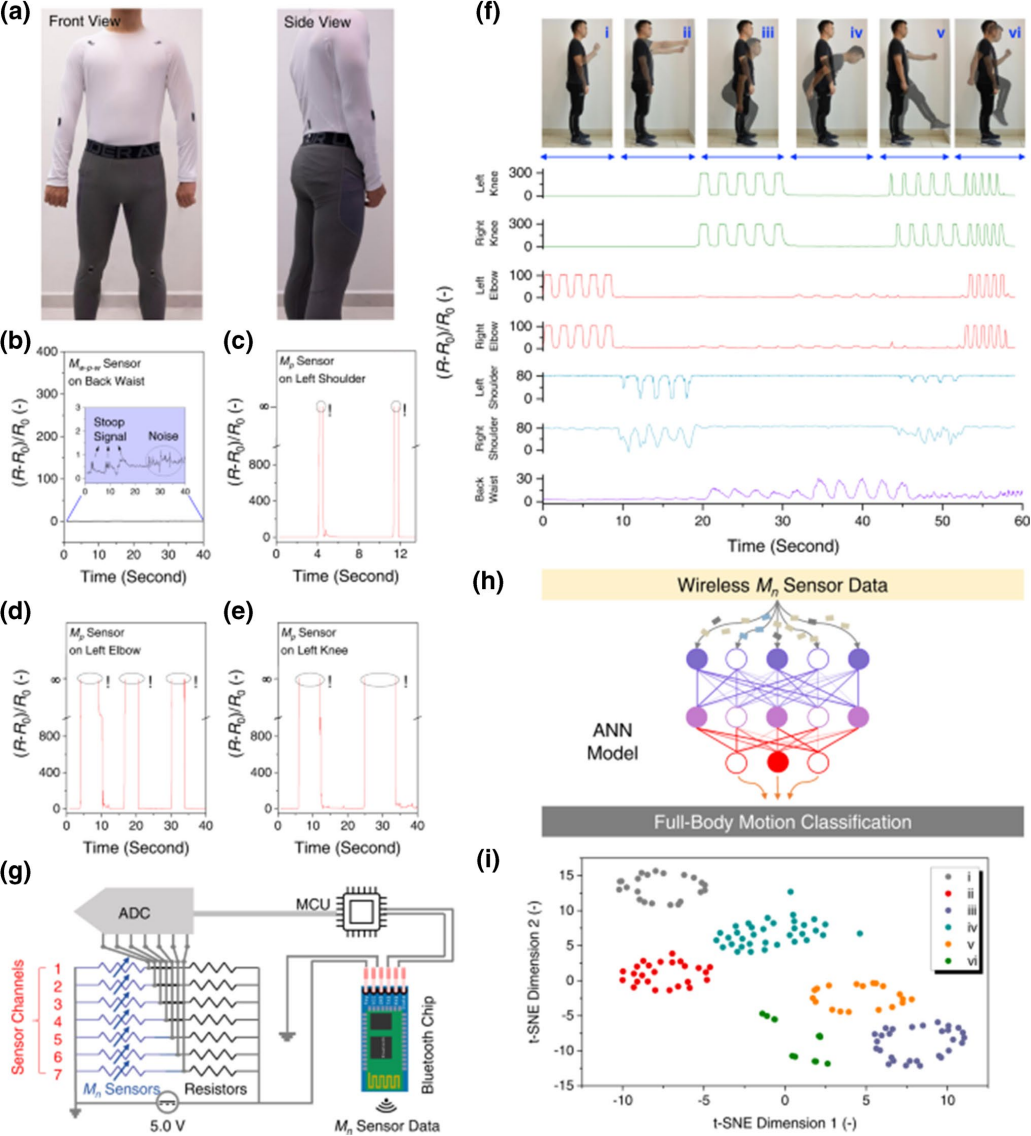
Wireless sensor module for full-body motion classification. **a** Photos of seven M_*n*_ sensors attached on the back waist (one *M*_p_), left/right shoulders (two *M*_p-w–p_), left/right elbows (two *M*_w_), and left/right knees (two *M*_w-p-w_) of a volunteer. **b** Signal outputs,* S*_*ε*_, of a *M*_w-p-w_ sensor attached on the back waist during repeated stoop motions were too small to be distinguished from noise signals. Signal outputs, *S*_ε_, of two M_*p*_ sensors attached on **c** the left shoulder, **d** the left elbow, and **e** the left knee during repeated movements. Symbol “!” indicates that the M_*p*_ sensors’ resistances increased to infinite, where M_*p*_ sensors lost their strain sensing capabilities. **f** Signal outputs, *S*_ε_, of seven *M*_*n*_ sensors for full-body motion monitoring, including (i) left/right elbow lifting, (ii) left/right shoulder lifting, (iii) squatting, (iv) stooping, (v) walking, and (vi) running. **g** Equivalent circuit of a wireless sensor module. **h** Multi-channeled *M*_*n*_ sensor data were collected to construct a high-accuracy artificial neural network model for full-body motion classification. **i** t-SNE scatterplot of six full-body motions, where the strain sensing data underwent the dimension reduction into two dimensionless parameters (i.e., t-SNE dimension 1 and dimension 2). Reproduced from open access publication [253]. Nature Publishing group

Yang et al. prepared wearable Ti_3_C_2_T_*x*_ MXene sensor modules with in-sensor machine learning models, either functioning through wireless streaming or edge computing, for full-body motion classifications and avatar reconstruction [253]. The wearable strain sensor modules due to topographic design on piezoresistive nanolayers performed ultrahigh sensitivities within the working windows that meet all joint deformation ranges. The edge sensor module was made through the integration of the wearable sensors with a machine learning chip enabling in-sensor reconstruction of high-precision avatar animations that mimic continuous full-body motions with an average avatar determination error of 3.5 cm, without additional computing devices (Fig. 30). The approach described in the article addresses the challenge in wearable sensors to enable transmission of high density data obtained from several sensors in an effective way followed by machine learning algorithms with power effective local computing [253].

### Other healthcare applications

A “hospital-on-a-chip” system has been demonstrated with multifunctional microneedle electrodes for biosensing and electrostimulation using highly stable MXene nanosheets [254]. Microneedles are composed of dozens of micron-sized needles that can be used as an effective and painless transdermal patch to puncture the skin for drug delivery or biosensing purposes since they are directly in contact with the dermal layer inside the human body. The wearable MXene nanosheet-based microneedles can sense the tiny electric potential difference generated from the human eye movements or muscle contraction from the human arm. Therefore, the diseases associated with neuromuscular abnormalities such as myasthenia gravis can be monitored—consequently, the transcutaneous electrical nerve stimulation treatment can be applied according to the feedback of the micro-sensors [254].

A self-powered, flexible, multimodal, MXene-based wearable device was developed for continuous, real-time physiological biosignal observation. The system included multipurpose electronics, very sensitive pressure sensors, and power-efficient triboelectric nanogenerators [255]. The main component was a 3D-printable MXene joined to a platform that resembled skin and had considerable stretchability and positive triboelectric characteristics. This self-powered physiological sensor device allowed for constant radial artery pulse waveform observation without the need for independent energy thanks to its sensitivity (6.03 kPa^−1^), power output (816.6 mW m^−2^), the limit of detection (9 Pa), and quick reaction time (80 ms). Near-field communication was used to transmit wireless data and power, as well as its continuous, on-demand, fully self-powered rapid assessment program supervision [255].

**Fig. 31:**
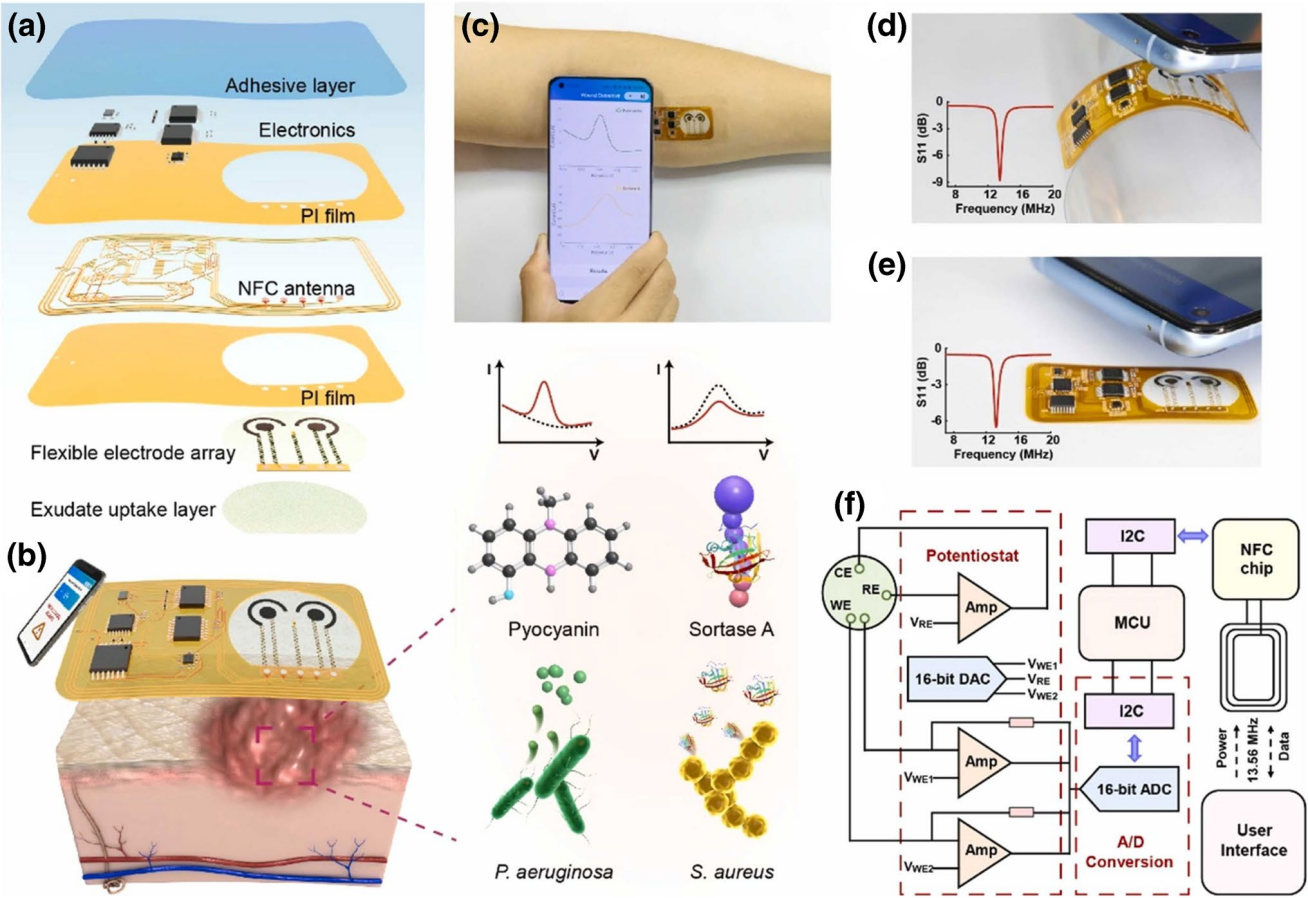
The wireless and battery-free smart bandage system. **a** Overall design of the smart bandage. **b** Schematic of the smart bandage system for in situ bacterial virulence factors detection. **c** Photo of the smart bandage interfaced on the arm, with a smartphone for wireless energy and data transmission. **d** The wireless communication between flexible circuit board and the near-field communication (NFC)–enabled mobile terminals during bending. The inset showed the resonant frequency of the circuit. **e** The wireless communication between flexible circuit board and the NFC-enabled mobile terminals under a communication distance. The inset shows the corresponding resonant frequency. **f** Block diagram and working principle of the system. WE, working electrode; CE, counter electrode; RE, reference electrode; Amp, operational amplifier; MCU, microcontroller unit; DAC, digital-to-analog converter; ADC, analog-to-digital converter; I2C, inter-integrated circuit. Reproduced with permission from ref. [256]. Copyright 2023 Elsevier

Wound infection is a life-threatening healthcare issue that can cause severe pain, sepsis, and even amputation. Typical biomarkers, sortase A and pyocyanin, corresponding to two major types of bacterial infection, Gram-positive *Staphylococcus aureus* and Gram-negative *Pseudomonas aeruginosa*, were detected with electrochemical DPV with Ti_3_C_2_T_*x*_ MXene applied to the electrode to enhance the sensitivity [256]. Integration of near-field communication module realized wireless energy harvesting and data transmission with a smartphone. The fully integrated system (Fig. 31) demonstrated good linearity and high sensitivity, with wide detection ranges from 1 pg mL^−1^ to 100 ng mL^−1^ for sortase A, and of 1 μM to 100 μM for pyocyanin. This wearable system provides a non-invasive, convenient, and efficient platform for in situ bacterial virulence factors detection, offering great potential for the management of the infected wound [256].

**Fig. 32:**
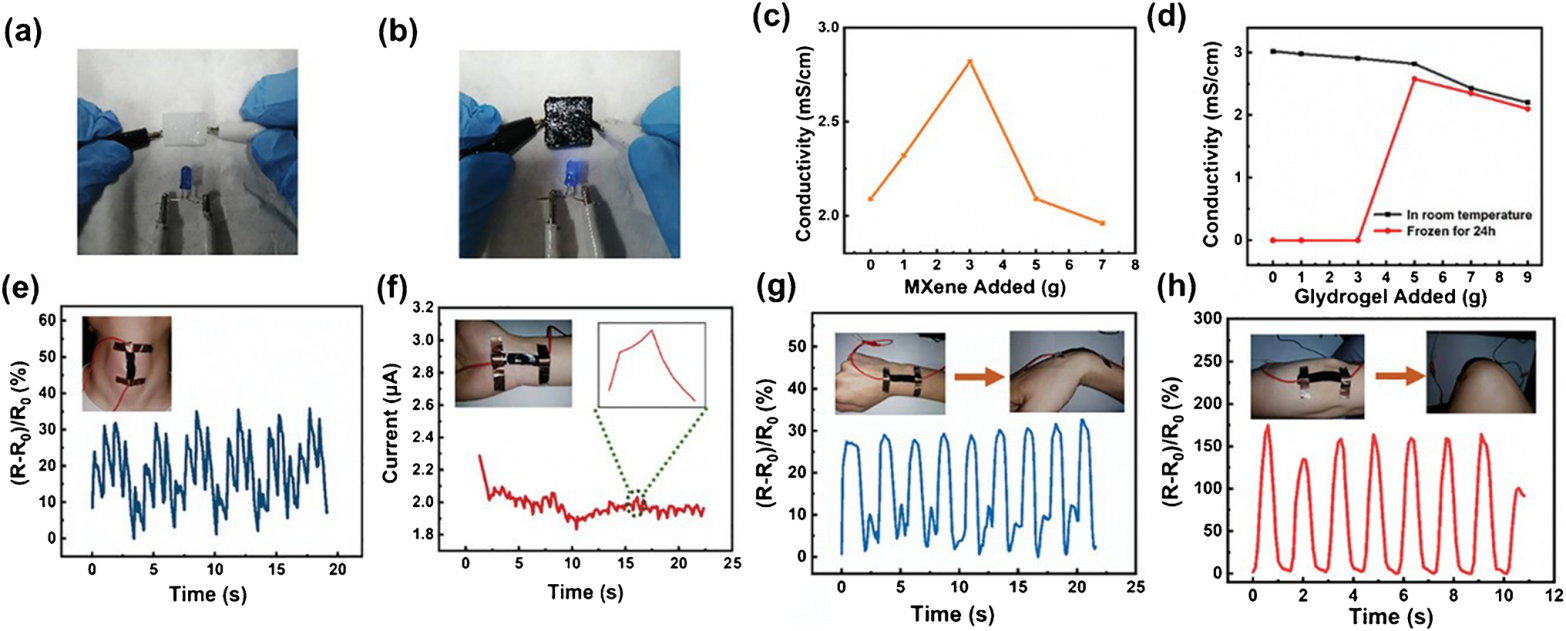
Comparison of the brightness of LEDs with **a** PVA and **b** PCMG hydrogels as conductors. **c** The conductivity of hydrogels with different MXene content. **d** The conductivity of hydrogels at room temperature and frozen at − 18 °C for 24 h with different glycerol content. Demonstration of the PCMG hydrogel-based sensors for human motion monitoring, **e** swallow, **f** wrist pulse, **g** wrist bent, and **h** elbow bent. Reproduced with permission from ref. [257]. Copyright 2021 John Wiley and Sons

Conductive hydrogels have received widespread attention in the applications of biosensors, human–machine interface, and health recording electrodes. The authors have developed the hydrogels with anti-freezing, anti-dehydration, and re-moldability using MXene as conductive material [257]. The resulting sensor had the characteristics of high sensitivity (gauge factor of 2.30), good linearity (*R*^2^ = 0.999), wide strain detection range (559%), and fast response (0.165 s). These excellent properties showed that the as-prepared conductive hydrogels have significance in promoting the construction of multifunctional wearable sensors. The hydrogel-based strain sensor can be used to monitor large strains and also has excellent sensitivity to micro strains (1–5%). They concluded that conductive PCMG hydrogels can realize the purpose of human motion detection accurately in harsh environment, opening up a new development path for flexible wearable sensors and ion skin (Fig. 32) [257].

## Summary

MXenes due to fascinating interfacial properties are 2D nanomaterials of choice for many different healthcare applications. The first MXene-based healthcare application was described in 2015 with an increasing interest to use such 2D nanomaterials for plethora of biomedical applications. Initially, MXenes were extensively applied as sensors for detection of various low-molecular-weight analytes including also hybrid nanoparticles used as nanozymes (peroxidase- and oxidase-like activities). There is, however, an increasing interest to apply MXene for construction of biosensors integrating bioaffinity probes (DNA/RNA, DNA aptamers and antibodies) for detection of high-molecular-weight analytes including also cancer biomarkers. Unfortunately, there are only few examples describing development and application of biosensors for analysis of such high-molecular-weight disease biomarkers. A separate application path is to apply MXene-based devices as wearable sensors for monitoring of human activities. Interestingly, there is already a prototype integrating several wearable sensors enabling reconstruction of avatar animation mimicking full body motions with high spatial precision/resolution. The authors believe that such approach can be applied for monitoring of movement in sports and also for underwater soft robots [253].

## Outlook

The beauty of using MXenes is their low cytotoxicity, for example upon degradation of Ti_3_C_2_T_*x*_ MXene nontoxic products (such as TiO_2_, CO_2_, or CH_4_ are produced), what can further accelerate their integration into many healthcare applications. The main challenges for MXene-based devices, which need to be addressed, are to prepare MXenes from MAX phases in a highly reproducible way with tailor made interfacial properties and to enhance stability of MXenes, when exposed to air or humidity. Furthermore, electrochemical MXene-based devices face another challenge, i.e., anodic oxidation significantly influencing electrochemical properties of such surfaces [170] (Table 1). This is why it is very important to properly choose redox mediator operating rather in a cathodic potential window such as Ru(NH_3_)_6_^3+^ [221]. The other issue is to make MXene or hybrid MXene interfaces biocompatible. MXene-based biosensors strongly rely on nanohybrid biocompatibility; thus, there should be focused research on the surface chemistry of MXenes to solve the problems based on the affinity and stability of biomolecules present on the MXene surfaces. One of the ways how to design biocompatible MXene interfaces is to use free plasmons for spontaneous grafting of (bio)polymers via aryldiazonium-based grafting [177]. In the case of wearable sensors, MXene nanomaterial is oxidized when it is continuously in contact with air. This reduces the conductivity and affects the sensing ability. However, on the other hand, the external polymer coating to prevent oxidation in the MXene affects the breathability and comfort of the wearable biosensors. Thus, an in-depth understanding is needed to design sensors that could maintain the conductivity of the MXene, while still being convenient for the user [258]. One approach toward right direction is to prepare wrinkle-free MXene layers with control of the crack propagation [253]. Furthermore, there is high potential to combine MXene affinity toward glycans (complex carbohydrates) [210] with electrochemical detection platform for detection of novel types of biomolecules, i.e., glycoproteins. Thus, we envisage that the future of MXene interfaces in combination with electrochemistry and other detection methods in the healthcare sector is very bright once the challenges described above will be properly addressed.

**Table 1:** A brief summary of electrochemical MXene patterned platforms utilized for healthcare applications

Analyte	Sensing platform	Detection method	LOD	Linear range	Ref
Glucose	GC/Ti_3_C_2_–HF/TBA/GOx/GTA	CA	23.0 μM	50–27 750 μM	[160]
Glucose, cholesterol	MXene/CTS/Cu_2_O	CV	49.8 μM glucose; 52.4 μM cholesterol	49.8–200 μM (glucose); 52.4 to 2000 μM (cholesterol)	[181]
Glucose	MXene graphene aerogel–Cu_2_O composite	CA	1.1 μM	0.1–14 and 15–40 mM	[182]
Glucose	GC/MXene (Ti_3_C_2_T_*x*_)-graphene/GOx	CV	0.10–0.13 mM	0.2–5.5 mM	[184]
Glucose	PEDOT:SCX/MXene/GOX/GCE	*i − t*	22.5 µM	0.5–8 mM	[185]
Glucose, lactate, and alcohol	Enzyme (GOx, LOx, AOx)/Ti_3_C_2_T_x_/PB/N-LSG	CA	0.3 μM glucose, 0.5 μM lactate/alcohol	10 μM–5.3 mM glucose, 0–20 mM lactate 0–50 mM alcohol	[187]
Glucose in sweat	Pt/MXene/GCE	CA	29.15 μM	0–8 mM	[189]
Glucose	Ti_3_C_2_-PLL-GOx/GCE	CV	2.6 µM	4.0–20 µM and 0.02–1.1 mM	[259]
UA, creatinine	MXene-Ti_3_C_2_T_*x*_/SPCE	SWV	5 × 10^−6^ M (UA); 1.2 × 10^−6^ M (creat.)	30–500 × 10^−6^ M (UA) and (10–400 × 10^−6^ M (creatinine)	[190]
Cholesterol	Chox-MXene/SA/SiO_2_@C22 MEPCM-GCE	CPA	0.081 µM	0.6–48.6 µM	[191]
H_2_O_2_	HRP/Ti_3_C_2_/Nafion/GCE	DPV	1 μM	5–8,000 μM	[192]
UA	NS-Ti_3_C_2_/GCE	CV	0.19 µM	2–400 µM	[196]
Kanamycin	GCE/MXene/VS_2_/capture DNA/DNA_2_’/CeCu_2_O_4_ nanozyme	SWV	0.6 pM	5 pM–5 μM	[197]
Zearalenone	SPE/MXene/Chitosan	EIS	0.4 pg mL^−1^	1 fg mL^−1^–1 ng mL^−1^	[198]
Sarcosine	SOx/MXene–chitosan/SPCE	CV	10.4 nM	up to 5 μM and 5–50 μM	[260]
Chlorpyrifos	MXene/AuPt/AChE-CS/GCE	DPV	1.55 pg mL^−1^	10^−8^–10^−3^ mg mL^−1^	[261]
epinephrine	GMA/ITO	DPV	3.5 nM	1–60 μM	[262]
Dopamine	T_i3_C_2_T_x_/GCE	*i* − *t*	3 nM	0.015–10 µM	[263]
Lactate	Ti_3_C_2_@Eu-SnO_2_/Lox/GCE	CV	3.38 × 10^−10^ mol L^−1^	1.0 × 10^−9^–1.0 × 10^−4^ mol L^−1^	[264]
SARS-CoV-2 target DNA	NH_2_-pDNA/Ti_3_C_2_T_*x*_/SPCE	EIS	0.004 pM	0.1 pM–1 μM	[83]
BRCA1 gene	HS-ssDNA/AuNP@BLM/dMXene/AuE	EIS	1 zM	10 zM to 1 μM	[215]
microRNA-21	M/MoS_2_/Thi/AuNPs/GCE		2.6 × 10^−14^ M	100 fM–100 nM	[216]
miRNA-122	ssDNA/AuHFGNs/PnBA-MXene/GCE	DPV	0.0035 aM	0.01 aM–10 nM	[217]
miRNA-141	BiOCl NSs@H2/miRNA/MCH/H1/ Ti_3_C_2_ Mxenes/CdS:W NCs/GCE	ECL	0.26 pM	0.6 pM–4000 pM	[218]
Human papillomavirus 18 DNA	AuNPs/MXene Ti_3_C_2_ based E-CRISPR	SWV	1.95 pM	10 pM–500 nM	[232]
miRNA221	GSH-MQDs/MGCE	ECL	10 fM	10 fM–10 nM	[265]
miRNA-377	DP-AuNPs/MCH/CP/MXene-Au/GCE	SWV	1.35 aM	10 aM–100 pM	[266]
miRNA-155	Exo III/miRNA-155/BSA/CDNA/AuNPs/Ti_3_C_2_ MXene/AuE	DPV	0.35 fM	1.0 fM to 10 nM	[267]
CA125	GCE-MXene/MIL-101(Fe)-NH_2_/UiO66@MB, GCE-MXene-CSMIL101-Ab1-Ag-Ab2-UiO66@MB	DPV	0.006 U mL^−1^	0.2–1000 U mL^−1^	[80]
cTnT	MXene@PAMAM/SPCE	DPV	0.069 ng mL^−1^	0.1–1000 ng mL^−1^	[201]
NGAL	Probe/NGAL/BSA/Ab1/Au/MXene/PANI/GCE (Probe: Pep/Au/Cu-MOF/SWNH)	SWV, *i* − *t*	0.0074 pg·mL^−1^ (SWV); 0.0405 pg·mL^−1^ (*i* − *t*)	0.00001–10 ng·mL^−1^	[202]
IgG	Pep/DBMH/GCE	DPV	23 pg mL^−1^	0.1 ng mL^−1^–10 μg mL	[205]
*β*-hCG	BSA/anti-*β*-hCG/EDC-NHS/Ag/Ti_3_C_2_T_*x*_	EIS	9.5 × 10^–3^ mIU mL^−1^	5.0 × 10^–2^–1.0 × 10^2^ mIU mL^−1^	[206]
PSA	Fc/peptide/MXene-Au-MB/GCE	DPV	0.83 pg mL^−1^	5 pg mL^−1^–10 ng mL^−1^	[224]
CEA	MCH/THC/Au–Pd-Pt/Ti_3_C_2_T_*x*_/GCE	DPV	0.32 fg mL^−1^	1 fg mL^−1^–1 ng mL^−1^	[225]
Osteopontin	Apt/PPy@Ti_3_C_2_T_*x*_/PMo_12_/AE	EIS	0.98 fg mL^−1^	0.05 pg mL^−1^–10.0 ng mL^−1^	[226]
CEA	*f*-graphene@Ti_3_C_2_-MXene/ITO	DPV	0.30 pg mL^−1^	0.01 pg mL^−1^–2000 ng mL^−1^	[229]
AFB1	Nafion/RLM/Au@MXene/GCE	*i* − *t*	2.8 nM	0.01 μM–50 μM	[204]
CD44	Ab1/PM/AuNPs/d-V_2_C/GCE, MB@NH_2_-Fe-MOF-Zn/Ab2/CD44/Ab1/PM/AuNPs/d-V_2_C/GCE	DPV	1.4 pg mL^−1^	0.5 ng mL^−1^–500 ng mL^−1^	[235]
PSA	BSA/anti-PSA/AuNPs/M-NTO-PEDOT/GCE	DPV	0.03 pg L^−1^	0.0001–20 ng mL^−1^	[268]
SARS-CoV-2 nucleocapsid protein (CoVNP)	BSA/AbMXene/P–BiOCl/Ru(bpy)_3_^2+/^GCE	ECL	0.49 fg mL^−1^	1 fg mL^−1^–10 ng mL^−1^	[269]
*E. coli*	NMXene-Man/SPE	EIS	10 CFU mL^−1^	10^1^–10^8^ CFU mL^−1^	[270]
*Mycobacterium. tuberculosis*	ssDNA/PPY/MXene/GCE	DPV	11.24 fM	100 fM–25 nM	[271]

*Ab* antibody; *AFB1* aflatoxin B1; *AChE* acetylcholinesterase from *Electrophorus electricus*; *AOx* alcohol oxidase; *AuE* gold electrode; *Apt* aptamer; *AuNP@BLM* gold nanoparticle-decorated biomimetic bilayer lipid membrane; *BRCA1 gene* BReast CAncer gene 1; *BSA* bovine serum albumin; *β-hCG* β-human chorionic gonadotropin; *CA* chronoamperommetry; *CA125* carbohydrate antigen 125; *c-DNA* capture DNA; *CD44* a family of cell adhesion molecules expressed on the surface of multiple cell types; *CEA* carcinoembryonic antigen; *CS* chitosan; *CP* DNA capture probe; *CPA* constant potential amperometry; *cTnT* human cardiac troponin T; *Cu-MOF/SWNH* copper metal–organic framework decorated with a single-walled carbon nanohorn; *CV* cyclic voltammetry; *DBMH* D-BSA/MXene hydrogel; *dMXene* delaminated MXene; *DP-AuNPs* G-rich detection probes modified on AuNPs; *dV*_*2*_*C* delaminated V_2_C; *DPV* differential pulse voltammetry; *ECL* electrochemiluminescence; *E. coli Escherichia coli* bacteria; *E-CRISPR* clustered regularly interspaced short palindromic repeats powered electrochemical sensor; *EIS* electrochemical impedance spectroscopy; *f-graphene* amine functionalized graphene; *Exo III* exonuclease III (Exo III)-aided cascade target recycling; *Fc* carboxyl-modified ferrocene; *GCE* glassy carbon electrode; *GMA* reduced graphene oxide/Ti_3_C_2_T_x_ MXene; *GOx* glucose oxidase; *GSH-MQDs* glutathione-capped MXene-derived quantum dots; *GTA* glutaraldehyde; *AuHFGNs/PnBA-MXene* hierarchical flower-like gold, poly (n-butyl acrylate), and MXene; *HS-ssDNA* thiolated single-stranded deoxyribonucleic acid; *HRP* horseradish peroxidase; *ChOx* cholesterol oxidase; *IgG* immunoglobulin G; *ITO* indium tin oxide; *LOx* lactate oxidase; *M* Ti_3_C_2_ MXene; *MCH* 6-Mercapto-1-hexanol; *MIL-101* chromium terephthalate metal–organic framework; trimeric chromium(III) octahedral clusters interconnected by 1,4-benzenedicarboxylates, resulting in a highly porous 3-dimentional structure; *MB* methylene blue; *MB@NH*_*2*_*-Fe-MOF-Zn* methylene blue/aminated metal organic framework; *MGCE* magnetic glassy carbon electrode; *M-NTO* 3D sodium titanate nanoribbons; *NGAL* neutrophil gelatinase–associated lipocalin; *N-LSG* nitrogen-doped laser-scribed graphene; *NH*_*2*_*-pDNA* amino-functionalized probe deoxyribonucleic acid; *NMXene-Man* nitrogen-doped MXene-mannose; *NS-Ti*_*3*_*C*_*2*_ Ti_3_C_2_ nanosheets; *PAMAM* poly(amidoamine) dendrimers; *PANI* polyaniline; *PBS* phosphate-buffered saline; *PEDOT:SCX* poly(3,4-ethylenedioxythiophene):4-sulfocalix [4]arene; *Pep* NGAL affinity peptide; *PM* platelet membrane; *PLL* poly-L-lysine; *PMo*_*12*_ phosphomolybdic acid; *PSA* prostate-specific antigen; *Ppy* polypyrrole; *RLM* rat liver microsome; *SiO*_*2*_*@C*_*22*_* MEPCM* core–shell structural microcapsules with an n-docosane core and a silica shell; *SOx* sarcosine oxidase; *SPCE* screen-printed carbon electrode; *SPE* screen-printed electrode; *SPR* surface plasmon resonance; *SWV* square wave voltammetry; *UiO66* a metal organic framework made up of [Zr_6_O_4_(OH)_4_] clusters with 1,4-benzodicarboxylic acid struts; *UV* ultraviolet; *Ti*_*3*_*C*_*2*_* − HF/TBA* Ti_3_C_2_ synthesized via HF etching and subsequent delamination by tetrabutylammonium hydroxide; *Thi* thionine; *THC* triple-helix complex.

## Data Availability

All data generated or analysed during this study are included in this published article.

## References

[1] Alam S, Chowdhury MA, Shahid A, Alam R, Rahim A (2021). Synthesis of emerging two-dimensional (2D) materials–advances, challenges and prospects. FlatChem.

[2] Kumbhakar P, Jayan JS, Sreedevi Madhavikutty A, Sreeram PR, Saritha A, Ito T, Tiwary CS (2023). Prospective applications of two-dimensional materials beyond laboratory frontiers: a review. IScience.

[3] Huang W, Zhang Y, Song M, Wang B, Hou H, Hu X, Chen X, Zhai T (2022). Encapsulation strategies on 2D materials for field effect transistors and photodetectors. Chin Chem Lett.

[4] Yang J, Liu X, Dong Q, Shen Y, Pan Y, Wang Z, Tang K, Dai X, Wu R, Jin Y (2022). Oxidations of two-dimensional semiconductors: fundamentals and applications. Chin Chem Lett.

[5] Gant P, Carrascoso F, Zhao Q, Ryu YK, Seitz M, Prins F, Frisenda R, Castellanos-Gomez A (2020). A system for the deterministic transfer of 2D materials under inert environmental conditions. 2D Materials.

[6] Naguib M, Kurtoglu M, Presser V, Lu J, Niu J, Heon M, Hultman L, Gogotsi Y, Barsoum MW (2011). Two-dimensional nanocrystals produced by exfoliation of Ti3AlC2. Adv Mater.

[7] Balendhran S, Walia S, Nili H, Sriram S, Bhaskaran M (2015). Elemental analogues of graphene: silicene, germanene, stanene, and phosphorene. Small.

[8] Ezawa M (2018) Electronic and topological properties of silicene, germanene and stanene. Silicene: Prediction, Synthesis, Application:43–71. Springer International Publishing. Eds. Vogt P and Le Lay G

[9] Lemme MC, Akinwande D, Huyghebaert C, Stampfer C (2022). 2D materials for future heterogeneous electronics. Nat Commun.

[10] Zhang C, Kremer MP, Seral-Ascaso A, Park SH, McEvoy N, Anasori B, Gogotsi Y, Nicolosi V (2018). Stamping of flexible, coplanar micro-supercapacitors using MXene inks. Adv Funct Mater.

[11] Verger L, Natu V, Carey M, Barsoum MW (2019). MXenes: an introduction of their synthesis, select properties, and applications. Trends Chem.

[12] VahidMohammadi A, Rosen J, Gogotsi Y (2021). The world of two-dimensional carbides and nitrides (MXenes). Science.

[13] Momma K, Izumi F (2011). VESTA 3 for three-dimensional visualization of crystal, volumetric and morphology data. J Appl Crystallogr.

[14] Brette F, Kourati D, Paris M, Loupias L, Célérier S, Cabioc’h T, Deschamps M, Boucher F, Mauchamp V (2023). Assessing the surface chemistry of 2D transition metal carbides (MXenes): a combined experimental/theoretical 13C solid state NMR approach. J Am Chem Soc.

[15] Jiang X, Kuklin AV, Baev A, Ge Y, Ågren H, Zhang H, Prasad PN (2020). Two-dimensional MXenes: from morphological to optical, electric, and magnetic properties and applications. Phys Rep.

[16] Shekhirev M, Shuck CE, Sarycheva A, Gogotsi Y (2021). Characterization of MXenes at every step, from their precursors to single flakes and assembled films. Prog Mater Sci.

[17] Naguib M, Barsoum MW, Gogotsi Y (2021). Ten years of progress in the synthesis and development of mxenes. Adv Mater.

[18] Abdul Ghani A, Shahzad A, Moztahida M, Tahir K, Jeon H, Kim B, Lee D (2021) Adsorption and electrochemical regeneration of intercalated Ti3C2Tx MXene for the removal of ciprofloxacin from wastewater. Chemical Engineering Journal 421(2):127780. 10.1016/j.cej.2020.127780

[19] Ciou J-H, Li S, Lee PS (2019). Ti3C2 MXene paper for the effective adsorption and controllable release of aroma molecules. Small.

[20] Ding L, Wei Y, Wang Y, Chen H, Caro J, Wang H (2017). A two-dimensional lamellar membrane: MXene nanosheet stacks. Angew Chem Int Ed.

[21] Karthikeyan P, Elanchezhiyan SS, Preethi J, Talukdar K, Meenakshi S, Park CM (2021). Two-dimensional (2D) Ti3C2Tx MXene nanosheets with superior adsorption behavior for phosphate and nitrate ions from the aqueous environment. Ceram Int.

[22] Kim S, Gholamirad F, Yu M, Park CM, Jang A, Jang M, Taheri-Qazvini N, Yoon Y (2021). Enhanced adsorption performance for selected pharmaceutical compounds by sonicated Ti3C2TX MXene. Chem Eng J.

[23] Le T, Jamshidi E, Beidaghi M, Esfahani MR (2022). Functionalized-MXene thin-film nanocomposite hollow fiber membranes for enhanced PFAS removal from water. ACS Appl Mater Interfaces.

[24] Li Z-K, Wei Y, Gao X, Ding L, Lu Z, Deng J, Yang X, Caro J, Wang H (2020). Antibiotics separation with MXene membranes based on regularly stacked high-aspect-ratio nanosheets. Angew Chem Int Ed.

[25] Meng F, Seredych M, Chen C, Gura V, Mikhalovsky S, Sandeman S, Ingavle G, Ozulumba T, Miao L, Anasori B, Gogotsi Y (2018). MXene sorbents for removal of urea from dialysate: a step toward the wearable artificial kidney. ACS Nano.

[26] Ren CE, Hatzell KB, Alhabeb M, Ling Z, Mahmoud KA, Gogotsi Y (2015). Charge- and size-selective ion sieving through Ti3C2Tx MXene membranes. The J Physical Chem Lett.

[27] Ren J, Zhu Z, Qiu Y, Yu F, Zhou T, Ma J, Zhao J (2021). Enhanced adsorption performance of alginate/MXene/CoFe2O4 for antibiotic and heavy metal under rotating magnetic field. Chemosphere.

[28] Tan YZ, Wang H, Han L, Tanis-Kanbur MB, Pranav MV, Chew JW (2018). Photothermal-enhanced and fouling-resistant membrane for solar-assisted membrane distillation. J Membr Sci.

[29] Unal MA, Bayrakdar F, Fusco L, Besbinar O, Shuck CE, Yalcin S, Erken MT, Ozkul A, Gurcan C, Panatli O, Summak GY, Gokce C, Orecchioni M, Gazzi A, Vitale F, Somers J, Demir E, Yildiz SS, Nazir H, Grivel J-C, Bedognetti D, Crisanti A, Akcali KC, Gogotsi Y, Delogu LG, Yilmazer A (2021). 2D MXenes with antiviral and immunomodulatory properties: a pilot study against SARS-CoV-2. Nano Today.

[30] Wang G, Zhang M, Chen D, Guo Q, Feng X, Niu T, Liu X, Li A, Lai J, Sun D, Liao Z, Wang Y, Chu PK, Ding G, Xie X, Di Z, Wang X (2018). Seamless lateral graphene p–n junctions formed by selective in situ doping for high-performance photodetectors. Nat Commun.

[31] Wang J, Chen P, Shi B, Guo W, Jaroniec M, Qiao S-Z (2018). A regularly channeled lamellar membrane for unparalleled water and organics permeation. Angew Chem Int Ed.

[32] Wang L, Yuan L, Chen K, Zhang Y, Deng Q, Du S, Huang Q, Zheng L, Zhang J, Chai Z, Barsoum MW, Wang X, Shi W (2016). Loading actinides in multilayered structures for nuclear waste treatment: the first case study of uranium capture with vanadium carbide MXene. ACS Appl Mater Interfaces.

[33] Ying Y, Liu Y, Wang X, Mao Y, Cao W, Hu P, Peng X (2015). Two-dimensional titanium carbide for efficiently reductive removal of highly toxic chromium(VI) from water. ACS Appl Mater Interfaces.

[34] Zandi P, Ghasemy E, Khedri M, Rashidi A, Maleki R, Miri Jahromi A (2021). Shedding light on miniaturized dialysis using MXene 2D materials: a computational chemistry approach. ACS Omega.

[35] Zhang Q, Teng J, Zou G, Peng Q, Du Q, Jiao T, Xiang J (2016). Efficient phosphate sequestration for water purification by unique sandwich-like MXene/magnetic iron oxide nanocomposites. Nanoscale.

[36] Zhao Q, Seredych M, Precetti E, Shuck CE, Harhay M, Pang R, Shan C-X, Gogotsi Y (2020). Adsorption of uremic toxins using Ti3C2Tx MXene for dialysate regeneration. ACS Nano.

[37] Massoumılari Ş, Velioǧlu S (2023). Can MXene be the effective nanomaterial family for the membrane and adsorption technologies to reach a sustainable green world?. ACS Omega.

[38] Kim Y-J, Kim SJ, Seo D, Chae Y, Anayee M, Lee Y, Gogotsi Y, Ahn CW, Jung H-T (2021). Etching mechanism of monoatomic aluminum layers during MXene synthesis. Chem Mater.

[39] Persson I, Halim J, Hansen TW, Wagner JB, Darakchieva V, Palisaitis J, Rosen J, Persson POÅ (2020). How much oxygen can a MXene surface take before it breaks?. Adv Func Mater.

[40] Iqbal A, Kwon J, Kim M-K, Koo C (2021). MXenes for electromagnetic interference shielding: experimental and theoretical perspectives. Mater Today Adv.

[41] He P, Liu Z-Y, Mao G-B, Liu Q, Zheng M-J, Zuo R-Z, Cao W-Q, Hou Z-L, Yuan J, Cao M-S (2022). MXene films: toward high-performance electromagnetic interference shielding and supercapacitor electrode. Compos Part A-Appl Sci.

[42] Yu Y, Yi P, Xu W, Sun X, Deng G, Liu X, Shui J, Yu R (2022). Environmentally tough and stretchable MXene organohydrogel with exceptionally enhanced electromagnetic interference shielding performances. Nano-Micro Lett.

[43] Oliveira FM, Azadmanjiri J, Wang X, Yu M, Sofer Z (2023) Structure design and processing strategies of MXene‐based materials for electromagnetic interference shielding. Small Methods 7(7):2300112 10.1002/smtd.202300112 37129581

[44] Li L, Fu X, Chen S, Uzun S, Levitt AS, Shuck CE, Han W, Gogotsi Y (2020). Hydrophobic and stable MXene–polymer pressure sensors for wearable electronics. ACS Appl Mater Interf.

[45] Wu L, Yuan X, Tang Y, Wageh S, Al-Hartomy OA, Al-Sehemi AG, Yang J, Xiang Y, Zhang H, Qin Y (2023). MXene sensors based on optical and electrical sensing signals: from biological, chemical, and physical sensing to emerging intelligent and bionic devices. PhotoniX.

[46] Mariano M, Mashtalir O, Antonio FQ, Ryu W-H, Deng B, Xia F, Gogotsi Y, Taylor AD (2016). Solution-processed titanium carbide MXene films examined as highly transparent conductors. Nanoscale.

[47] Hantanasirisakul K, Gogotsi Y (2018). Electronic and optical properties of 2D transition metal carbides and nitrides (MXenes). Adv Mater.

[48] Han M, Maleski K, Shuck CE, Yang Y, Glazar JT, Foucher AC, Hantanasirisakul K, Sarycheva A, Frey NC, May SJ (2020). Tailoring electronic and optical properties of MXenes through forming solid solutions. J Am Chem Soc.

[49] Yang Z, Yang Q, Tian Y, Ren X, Li C, Zu Y, Din SZU, Gao L, Wu J, Chen H (2023). Few-layer Ti3CN MXene for ultrafast photonics applications in visible band. J Materiom.

[50] Shang C, Zhang Y, Wang G, Sun J, Cheng Y, Zhang Y-B, Yao B, Fu B, Li J (2022). Nonlinear optical properties of MXene and applications in broadband ultrafast photonics. J Alloys Compd.

[51] Pacheco-Peña V, Hallam T, Healy N (2022). MXene supported surface plasmons on telecommunications optical fibers. Light Sci Appl.

[52] Wang Y, Xu Y, Hu M, Ling H, Zhu X (2020). MXenes: focus on optical and electronic properties and corresponding applications. Nanophotonics.

[53] Patra A, MB B, Manasa G, Samal AK, Rout CS (2023). 2D MXenes as a promising candidate for surface enhanced raman spectroscopy: state of the art, recent trends, and future prospects. Adv Funct Mater.

[54] He Z, Rong T, Li Y, Ma J, Li Q, Wu F, Wang Y, Wang F (2022). Two-dimensional TiVC solid-solution MXene as surface-enhanced Raman scattering substrate. ACS Nano.

[55] Zhou X, Wen J, Wang Z, Ma X, Wu H (2022). Broadband high-performance microwave absorption of the single-layer Ti3C2Tx MXene. J Mater Sci Technol.

[56] Li R, Zhang L, Shi L, Wang P (2017). MXene Ti3C2: an effective 2D light-to-heat conversion material. ACS Nano.

[57] Fang M, Li Q, Yang D, Zhou B, Feng Y, Liu C (2023). Synergistic light-to-heat conversion effect of MXene-based transparent film with insulating PDMS/Fe3O4 coating. Compos Part A-Appl Sci.

[58] Sobolčiak P, Ali A, Hassan MK, Helal MI, Tanvir A, Popelka A, Al-Maadeed MA, Krupa I, Mahmoud KA (2017). 2D Ti3C2Tx (MXene)-reinforced polyvinyl alcohol (PVA) nanofibers with enhanced mechanical and electrical properties. PLoS ONE.

[59] Filip J, Zavahir S, Lorencova L, Bertók T, Bin Yousaf A, Mahmoud K, Tkac J, Kasák P (2019). Tailoring electrocatalytic properties of Pt nanoparticles grown on Ti 3 C 2 T X MXene surface. J Electrochem Soc.

[60] Tanvir A, Sobolčiak P, Popelka A, Mrlik M, Spitalsky Z, Micusik M, Prokes J, Krupa I (2019). Electrically conductive, transparent polymeric nanocomposites modified by 2D Ti3C2Tx (MXene). Polymers.

[61] Zavahir S, Sobolčiak P, Krupa I, Han DS, Tkac J, Kasak P (2020). Ti3C2T x MXene-based light-responsive hydrogel composite for bendable bilayer photoactuator. Nanomaterials.

[62] Popelka A, Padmanabhan AC, Elgendy AS, Sobolciak P, Krupa I, Yousaf AB, Šebesta M, Tkac J, Kasak P (2023). Perfluoroctylsilane grafted Ti3C2X-based hydrogel liquid marble for controlled movement, self-assembly, light-induced release, and water evaporation system. Mater Today Commun.

[63] Bilibana MP (2023). Electrochemical properties of MXenes and applications. Adv Sens Energy Mater.

[64] Iravani S, Varma RS (2023). MXene-based wearable supercapacitors and their transformative impact on healthcare. Mater Adv.

[65] Rabiee N, Iravani S (2023). MXenes and their composites: a versatile platform for biomedical applications. Mater Chem Horizons.

[66] Anasori B, Lukatskaya MR, Gogotsi Y (2017). 2D metal carbides and nitrides (MXenes) for energy storage. Nat Rev Mater.

[67] Li X, Huang Z, Shuck CE, Liang G, Gogotsi Y, Zhi C (2022). MXene chemistry, electrochemistry and energy storage applications. Nat Rev Chem.

[68] Lokhande P, Pakdel A, Pathan H, Kumar D, Vo D-VN, Al-Gheethi A, Sharma A, Goel S, Singh PP, Lee B-K (2022). Prospects of MXenes in energy storage applications. Chemosphere.

[69] Shinde PA, Patil AM, Lee S, Jung E, Jun SC (2022). Two-dimensional MXenes for electrochemical energy storage applications. J Mater Chem A.

[70] Chen Y, Yang H, Han Z, Bo Z, Yan J, Cen K, Ostrikov KK (2022). MXene-based electrodes for supercapacitor energy storage. Energy Fuels.

[71] Shamsudeen Seenath J (2022) Energy storage applications of MXene. In: Khalid M, Grace AN, Arulraj A, Numan A (eds) Fundamental Aspects and Perspectives of MXenes. Engineering Materials. Springer International Publishing, Cham, 139–169. 10.1007/978-3-031-05006-0_7

[72] Nahirniak S, Ray A, Saruhan B (2023). Challenges and future prospects of the MXene-based materials for energy storage applications. Batteries.

[73] Gupta M, Verma A, Chaudhary P, Yadav B (2023). MXene and their integrated composite-based acetone sensors for monitoring of diabetes. Mater Adv.

[74] Sobolčiak P, Tanvir A, Sadasivuni KK, Krupa I (2019). Piezoresistive sensors based on electrospun mats modified by 2D Ti3C2Tx MXene. Sensors.

[75] Hajian S, Maddipatla D, Narakathu BB, Atashbar MZ (2022). MXene-based flexible sensors: a review. Front Sens.

[76] Xie K, Wang J, Xu S, Hao W, Zhao L, Huang L, Wei Z (2023). Application of two-dimensional MXene materials in sensors. Mater Des.

[77] Babar ZUD, Della Ventura B, Velotta R, Iannotti V (2022). Advances and emerging challenges in MXenes and their nanocomposites for biosensing applications. RSC Adv.

[78] Yang G, Liu F, Zhao J, Fu L, Gu Y, Qu L, Zhu C, Zhu J-J, Lin Y (2023). MXenes-based nanomaterials for biosensing and biomedicine. Coord Chem Rev.

[79] Amara U, Hussain I, Ahmad M, Mahmood K, Zhang K (2023). 2D MXene-Based Biosensing: A Review. Small.

[80] Qu L, Wu M, Zhao L, Li J, Pan H (2023). A sandwich electrochemical immunosensor based on MXene@ dual MOFs for detection of tumor marker CA125. Microchim Acta.

[81] Bai Z, Zhao L, Feng H, Xin Z, Wang C, Liu Z, Tian M, Zhang H, Bai Y, Feng F (2023). Aptamer modified Ti3C2 nanosheets application in smart targeted photothermal therapy for cancer. Cancer Nanotechnol.

[82] Bilal M, Singh AK, Iqbal HM, Boczkaj G (2023). Enzyme-conjugated MXene nanocomposites for biosensing and biocatalysis acuities. Chem Eng J.

[83] Bharti A, Singh S, Munthala D, Roy S, Pojprapai S, Suksaweang S, Sain S, Roy SS, Mohamed JJ, Avasthi DK (2023). Development of a nucleic acid-based screen printed electrochemical biosensor using Ti3C2Tx-MXene for the detection of SARS-CoV-2. Microchem J.

[84] Li X, Bai Y, Shi X, Su N, Nie G, Zhang R, Nie H, Ye L (2021). Applications of MXene (Ti 3 C 2 T x) in photocatalysis: a review. Mater Adv.

[85] Kitchamsetti N, de Barros AL (2023). Recent advances in MXenes based composites as photocatalysts: synthesis, properties and photocatalytic removal of organic contaminants from wastewater. ChemCatChem.

[86] Sun J, Kong W, Jin Z, Han Y, Ma L, Ding X, Niu Y, Xu Y (2020). Recent advances of MXene as promising catalysts for electrochemical nitrogen reduction reaction. Chin Chem Lett.

[87] Pan X, Yang X, Yu M, Lu X, Kang H, Yang M-Q, Qian Q, Zhao X, Liang S, Bian Z (2023). 2D MXenes polar catalysts for multi-renewable energy harvesting applications. Nat Commun.

[88] Sun Y, Meng X, Dall’Agnese Y, Dall’Agnese C, Duan S, Gao Y, Chen G, Wang X-F (2019). 2D MXenes as co-catalysts in photocatalysis: synthetic methods. Nano-Micro Lett.

[89] Wang C, Guan S, Zhang H, Shen R, Yuan H, Li B (2023). Perspectives on two-dimensional ultra-thin materials in energy catalysis and storage. APL Mater.

[90] Kuznetsov DA, Chen Z, Kumar PV, Tsoukalou A, Kierzkowska A, Abdala PM, Safonova OV, Fedorov A, Müller CR (2019). Single site cobalt substitution in 2D molybdenum carbide (MXene) enhances catalytic activity in the hydrogen evolution reaction. J Am Chem Soc.

[91] Chen J, Li Z, Ni F, Ouyang W, Fang X (2020). Bio-inspired transparent MXene electrodes for flexible UV photodetectors. Mater Horizons.

[92] Guo T, Zhou D, Deng S, Jafarpour M, Avaro J, Neels A, Heier J, Zhang C (2023). Rational design of Ti3C2T x MXene inks for conductive, transparent films. ACS Nano.

[93] Bhat A, Anwer S, Bhat KS, Mohideen MIH, Liao K, Qurashi A (2021). Prospects challenges and stability of 2D MXenes for clean energy conversion and storage applications. NPJ 2D Mater Appl.

[94] An H, Habib T, Shah S, Gao H, Radovic M, Green MJ, Lutkenhaus JL (2018). Surface-agnostic highly stretchable and bendable conductive MXene multilayers. Sci Adv.

[95] Perini G, Rosenkranz A, Friggeri G, Zambrano D, Rosa E, Augello A, Palmieri V, De Spirito M, Papi M (2022). Advanced usage of Ti3C2Tx MXenes for photothermal therapy on different 3D breast cancer models. Biomed Pharmacother.

[96] Lu H, Wang J, Li H, Zhou W, Yuan Q, Liu S (2023). Efficient photothermal conversion of MXenes and their application in biomedicine. Mater Chem Front.

[97] Velusamy DB, El-Demellawi JK, El-Zohry AM, Giugni A, Lopatin S, Hedhili MN, Mansour AE, Fabrizio ED, Mohammed OF, Alshareef HN (2019). MXenes for plasmonic photodetection. Adv Mater.

[98] Liu Z, El-Demellawi JK, Bakr OM, Ooi BS, Alshareef HN (2022). Plasmonic Nb2C T x MXene-MAPbI3 heterostructure for self-powered visible-NIR photodiodes. ACS Nano.

[99] Huang D, Kim H, Zou G, Xu X, Zhu Y, Ahmad K, Almutairi ZA, Alshareef HN (2022). All-MXene thermoelectric nanogenerator. Mater Today. Energy.

[100] Lu X, Zhang Q, Liao J, Chen H, Fan Y, Xing J, Gu S, Huang J, Ma J, Wang J (2020). High-efficiency thermoelectric power generation enabled by homogeneous incorporation of MXene in (Bi, Sb) 2Te3 matrix. Adv Energy Mater.

[101] Zhu M, Lu C, Liu L (2023). Progress and challenges of emerging MXene based materials for thermoelectric applications. Iscience.

[102] Moghaddasi A, Sobolčiak P, Popelka A, Krupa I (2020). Separation of water/oil emulsions by an electrospun copolyamide mat covered with a 2D Ti3C2Tx MXene. Materials.

[103] Huang L, Ding L, Caro J, Wang H (2023) MXene‐based membranes for drinking water production. Angew Chem Int Ed 62(52):e202311138 10.1002/anie.202311138 37615530

[104] Yousaf T, Areeb A, Murtaza M, Munir A, Khan Y, Waseem A (2022). Silane-grafted MXene (Ti3C2T X) membranes for enhanced water purification performance. ACS Omega.

[105] Al-Hamadani YA, Jun B-M, Yoon M, Taheri-Qazvini N, Snyder SA, Jang M, Heo J, Yoon Y (2020). Applications of MXene-based membranes in water purification: a review. Chemosphere.

[106] Xie X, Chen C, Zhang N, Tang Z-R, Jiang J, Xu Y-J (2019). Microstructure and surface control of MXene films for water purification. Nat Sustainab.

[107] Li E, Gao C, Yu R, Wang X, He L, Hu Y, Chen H, Chen H, Guo T (2022). MXene based saturation organic vertical photoelectric transistors with low subthreshold swing. Nat Commun.

[108] Guha S, Kabiraj A, Mahapatra S (2022). High-throughput design of functional-engineered MXene transistors with low-resistive contacts. NPJ Comput Mater.

[109] Baraneedharan P, Shankari D, Arulraj A, Sephra PJ, Mangalaraja R, Khalid M (2023). Nanoengineering of MXene-Based Field-Effect transistor gas sensors: advancements in next-generation electronic devices. J Electrochem Soc.

[110] Liu F, Zhou A, Chen J, Jia J, Zhou W, Wang L, Hu Q (2017). Preparation of Ti3C2 and Ti2C MXenes by fluoride salts etching and methane adsorptive properties. Appl Surf Sci.

[111] Anayee M, Kurra N, Alhabeb M, Seredych M, Hedhili MN, Emwas A-H, Alshareef HN, Anasori B, Gogotsi Y (2020). Role of acid mixtures etching on the surface chemistry and sodium ion storage in Ti 3 C 2 T x MXene. Chem Commun.

[112] Natu V, Pai R, Sokol M, Carey M, Kalra V, Barsoum MW (2020). 2D Ti3C2Tz MXene synthesized by water-free etching of Ti3AlC2 in polar organic solvents. Chem.

[113] Zhao X, Radovic M, Green MJ (2020). Synthesizing MXene nanosheets by water-free etching. Chem.

[114] Jawaid A, Hassan A, Neher G, Nepal D, Pachter R, Kennedy WJ, Ramakrishnan S, Vaia RA (2021). Halogen etch of Ti3AlC2 MAX phase for MXene fabrication. ACS Nano.

[115] Khan U, Luo Y, Kong LB, Que W (2022). Synthesis of fluorine free MXene through lewis acidic etching for application as electrode of proton supercapacitors. J Alloys Compd.

[116] Alhabeb M, Maleski K, Anasori B, Lelyukh P, Clark L, Sin S, Gogotsi Y (2017). Guidelines for synthesis and processing of two-dimensional titanium carbide (Ti3C2Tx MXene). Chem Mater.

[117] Li H, Li X, Liang J, Chen Y (2019). Hydrous RuO2-decorated MXene coordinating with silver nanowire inks enabling fully printed micro-supercapacitors with extraordinary volumetric performance. Adv Energy Mater.

[118] Zhang C, McKeon L, Kremer MP, Park S-H, Ronan O, Seral-Ascaso A, Barwich S, Coileáin CÓ, McEvoy N, Nerl HC (2019). Additive-free MXene inks and direct printing of micro-supercapacitors. Nat Commun.

[119] Wu Z, Liu S, Hao Z, Liu X (2023). MXene contact engineering for printed electronics. Adv Sci.

[120] Raagulan K, Braveenth R, Kim BM, Lim KJ, Lee SB, Kim M, Chai KY (2020). An effective utilization of MXene and its effect on electromagnetic interference shielding: flexible, free-standing and thermally conductive composite from MXene–PAT–poly (p-aminophenol)–polyaniline co-polymer. RSC Adv.

[121] Oh HG, Park S-K (2023). Facile spray-drying process for the synthesis of hollow 3D MXene-encapsulated CoSnO3 nanoboxes with enhanced lithium storage properties. J Alloys Compd.

[122] Yarali E, El-Demellawi JK, Faber H, Naphade D, Lin Y, Loganathan K, Alghamdi WS, Xu X, Rehman Au, Aydin E (2023). Fully sprayed metal oxide transistors utilizing Ti3C2T x MXene contacts. ACS Appl Electron Mater.

[123] Zhou T, Yu Y, He B, Wang Z, Xiong T, Wang Z, Liu Y, Xin J, Qi M, Zhang H (2022). Ultra-compact MXene fibers by continuous and controllable synergy of interfacial interactions and thermal drawing-induced stresses. Nat Commun.

[124] Zhang J, Uzun S, Seyedin S, Lynch PA, Akuzum B, Wang Z, Qin S, Alhabeb M, Shuck CE, Lei W (2020). Additive-free MXene liquid crystals and fibers. ACS Central Sci.

[125] Wang J, He J, Kan D, Chen K, Song M, Huo W (2022). MXene film prepared by vacuum-assisted filtration: properties and applications. Crystals.

[126] Wang S, Li Z, Wang G, Wang Y, Ling Z, Li C (2021). Freestanding Ti3C2T x MXene/Prussian Blue analogues films with superior ion uptake for efficient capacitive deionization by a dual pseudocapacitance effect. ACS Nano.

[127] Ling Z, Ren CE, Zhao M-Q, Yang J, Giammarco JM, Qiu J, Barsoum MW, Gogotsi Y (2014). Flexible and conductive MXene films and nanocomposites with high capacitance. Proc Natl Acad Sci USA.

[128] Etman AS, Halim J, Rosen J (2021). Mo1. 33CTz–Ti3C2Tz mixed MXene freestanding films for zinc-ion hybrid supercapacitors. Mater Today Energy.

[129] Seredych M, Shuck CE, Pinto D, Alhabeb M, Precetti E, Deysher G, Anasori B, Kurra N, Gogotsi Y (2019). High-temperature behavior and surface chemistry of carbide MXenes studied by thermal analysis. Chem Mater.

[130] Deysher G, Shuck CE, Hantanasirisakul K, Frey NC, Foucher AC, Maleski K, Sarycheva A, Shenoy VB, Stach EA, Anasori B (2019). Synthesis of Mo4VAlC4 MAX phase and two-dimensional Mo4VC4 MXene with five atomic layers of transition metals. ACS Nano.

[131] Shuck CE, Sarycheva A, Anayee M, Levitt A, Zhu Y, Uzun S, Balitskiy V, Zahorodna V, Gogotsi O, Gogotsi Y (2020). Scalable synthesis of Ti3C2Tx mxene. Adv Eng Mater.

[132] Wang X, Zhou Y (2002). Microstructure and properties of Ti3AlC2 prepared by the solid–liquid reaction synthesis and simultaneous in-situ hot pressing process. Acta Mater.

[133] Li Z, Wang L, Sun D, Zhang Y, Liu B, Hu Q, Zhou A (2015). Synthesis and thermal stability of two-dimensional carbide MXene Ti3C2. Mat Sci Eng B.

[134] Tzenov NV, Barsoum MW (2000). Synthesis and characterization of Ti3AlC2. J Am Ceram Soc.

[135] Zhou A, Barsoum M (2010) Kinking nonlinear elastic deformation of Ti_3_AlC_2_, Ti_2_AlC, Ti_3_Al(C_0.5_, N_0.5_)_2_ and Ti_2_Al(C_0.5_, N_0.5_). J Alloys Compd 498(1):62–70

[136] Hendaoui A, Andasmas M, Amara A, Benaldjia A, Langlois P, Vrel D (2008). SHS of high-purity MAX compounds in the Ti-Al-C system. Int J Self-Propag High-Temp Synth.

[137] Yeh C, Kuo C, Chu Y (2010). Formation of Ti3AlC2/Al2O3 and Ti2AlC/Al2O3 composites by combustion synthesis in Ti–Al–C–TiO2 systems. J Alloys Compd.

[138] Mukasyan A, Shuck C (2017). Kinetics of SHS reactions: a review. Int J Self-Propag High-Temp Synth.

[139] Akhlaghi M, Tayebifard SA, Salahi E, Asl MS, Schmidt G (2018). Self-propagating high-temperature synthesis of Ti3AlC2 MAX phase from mechanically-activated Ti/Al/graphite powder mixture. Ceram Int.

[140] Sergiienko SA, Lopes DV, Constantinescu G, Ferro MC, Shchaerban ND, Tursunov OB, Shkepu VI, Pazniak H, Tabachkova NY, Castellón ER (2021). MXene-containing composite electrodes for hydrogen evolution: material design aspects and approaches for electrode fabrication. Int J Hydrogen Energy.

[141] Hamm CM, Schäfer T, Zhang H, Birkel CS (2016). Non-conventional synthesis of the 413 MAX phase V4AlC3. Z Anorg Allg Chem.

[142] Tran MH, Schäfer T, Shahraei A, Dürrschnabel M, Molina-Luna L, Kramm UI, Birkel CS (2018). Adding a new member to the MXene family: synthesis, structure, and electrocatalytic activity for the hydrogen evolution reaction of V4C3T x. ACS Appl Energy Mater.

[143] Zhu J, Zhang J, Lin R, Fu B, Song C, Shang W, Tao P, Deng T (2021). Rapid one-step scalable microwave synthesis of Ti 3 C 2 T x MXene. Chem Commun.

[144] Abdah MAAM, Cherusseri J, Dzulkarnain NA, Mokhtar M, Su'ait MS, Tan YS, Mustafa MN, Khalid M, Numan A, Radwan A (2023). Facile synthesis of microwave-etched Ti3C2 MXene/activated carbon hybrid for lithium-ion battery anode. J Electroanal Chem.

[145] Wang D, Si J, Lin S, Zhang R, Huang Y, Yang J, Lu W, Zhu X, Sun Y (2020). Achieving Macroscopic V4C3T x MXene by Selectively Etching Al from V4AlC3 Single Crystals. Inorg Chem.

[146] Wang F, Wang S, Tian F, Wang F, Xia X, Zhang Q, Pang Z, Yu X, Li G, Hsu H-Y (2023) Advances in molten-salt-assisted synthesis of 2D MXenes and their applications in electrochemical energy storage and conversion. Chem Eng J 470:144185

[147] Liu L, Zschiesche H, Antonietti M, Gibilaro M, Chamelot P, Massot L, Rozier P, Taberna PL, Simon P (2023). In situ synthesis of MXene with tunable morphology by electrochemical etching of MAX phase prepared in molten salt. Adv Energy Mater.

[148] Zhou A, Wang C-A, Hunag Y (2003). Synthesis and mechanical properties of Ti 3 AlC 2 by spark plasma sintering. J Mater Sci.

[149] Zhou W, Mei B, Zhu J, Hong X (2005). Synthesis of high-purity Ti 3 SiC 2 and Ti 3 AlC 2 by spark plasma sintering (SPS) technique. J Mater Sci.

[150] Zou Y, Sun Z, Tada S, Hashimoto H (2007). Rapid synthesis of single-phase Ti3AlC2 through pulse discharge sintering a TiH2/Al/TiC powder mixture. Scripta Mater.

[151] Petrus M, Woźniak J, Cygan T, Lachowski A, Moszczyńska D, Adamczyk-Cieślak B, Rozmysłowska-Wojciechowska A, Wojciechowski T, Ziemkowska W, Jastrzębska A (2021). Influence of Ti3C2Tx MXene and surface-modified Ti3C2Tx MXene addition on microstructure and mechanical properties of silicon carbide composites sintered via spark plasma sintering method. Materials.

[152] Hossein-Zadeh M, Mirzaee O, Mohammadian-Semnani H (2019). An investigation into the microstructure and mechanical properties of V4AlC3 MAX phase prepared by spark plasma sintering. Ceram Int.

[153] Siebert JP, Bischoff L, Lepple M, Zintler A, Molina-Luna L, Wiedwald U, Birkel CS (2019). Sol–gel based synthesis and enhanced processability of MAX phase Cr 2 GaC. J Mater Chem C.

[154] Pang Z, Zou X, Li S, Tang W, Xu Q, Lu X (2020). Molten Salt Electrochemical Synthesis of Ternary Carbide Ti3AlC2 from Titanium-Rich Slag. Adv Eng Mater.

[155] Istomin P, Istomina E, Nadutkin A, Grass V, Presniakov M (2016). Synthesis of a bulk Ti4SiC3 MAX phase by reduction of TiO2 with SiC. Inorg Chem.

[156] Hamm CM, Bocarsly JD, Seward G, Kramm UI, Birkel CS (2017). Non-conventional synthesis and magnetic properties of MAX phases (Cr/Mn) 2 AlC and (Cr/Fe) 2 AlC. J Mater Chem C.

[157] Hamm CM, Dürrschnabel M, Molina-Luna L, Salikhov R, Spoddig D, Farle M, Wiedwald U, Birkel CS (2018). Structural, magnetic and electrical transport properties of non-conventionally prepared MAX phases V 2 AlC and (V/Mn) 2 AlC. Mater Chem Front.

[158] Chen Q, Zhang D, Pan J, Fan W (2020). Optical properties of two-dimensional semi-conductive MXene Sc2COx produced by sputtering. Optik.

[159] Horak P, Bakardjieva S, Vacik J, Rui X, Klie R (2020). Preparation of Ti2C MXene phase by ion beam sputtering and ion irradiation. Nucl Instrum Methods Phys Res, Sect B.

[160] Chia HL, Mayorga-Martinez CC, Antonatos N, Sofer Z, Gonzalez-Julian JJ, Webster RD, Pumera M (2020). MXene titanium carbide-based biosensor: strong dependence of exfoliation method on performance. Anal Chem.

[161] Thakur A, Chandran BSN, Davidson K, Bedford A, Fang H, Im Y, Kanduri V, Wyatt BC, Nemani SK, Poliukhova V (2023). Step-by-step guide for synthesis and delamination of Ti3C2Tx MXene. Small Methods.

[162] Gajdosova V, Lorencova L, Prochazka M, Omastova M, Micusik M, Prochazkova S, Kveton F, Jerigova M, Velic D, Kasak P, Tkac J (2020) Remarkable differences in the voltammetric response towards hydrogen peroxide, oxygen and Ru(NH3)6 3+ of electrode interfaces modified with HF or LiF-HCl etched Ti3C2Tx MXene. Microchimica Acta 187(1):52. 10.1007/s00604-019-4049-6 10.1007/s00604-019-4049-6 31848717

[163] Gogotsi Y, Huang Q (2021). MXenes: two-dimensional building blocks for future materials and devices. ACS Nano.

[164] Bhardwaj SK, Singh H, Khatri M, Kim K-H, Bhardwaj N (2022). Advances in MXenes-based optical biosensors: a review. Biosens Bioelectron.

[165] Liu H, Xing X, Tan Y, Dong H (2022). Two-dimensional transition metal carbides and nitrides (MXenes) based biosensing and molecular imaging. Nanophotonics.

[166] Li H, Fan R, Zou B, Yan J, Shi Q, Guo G (2023). Roles of MXenes in biomedical applications: recent developments and prospects. J Nanobiotechnology.

[167] Zhang J, Usman KAS, Judicpa MAN, Hegh D, Lynch PA, Razal JM (2023). Applications of X-ray-based characterization in MXene research. Small Methods.

[168] Sarycheva A, Gogotsi Y (2020). Raman spectroscopy analysis of the structure and surface chemistry of Ti3C2Tx MXene. Chem Mater.

[169] Vargas FL, Klie RF (2023). Electron probe interactions in single species-terminated MXenes. Microsc Microanal.

[170] Lorencova L, Bertok T, Dosekova E, Holazova A, Paprckova D, Vikartovska A, Sasinkova V, Filip J, Kasak P, Jerigova M, Velic D, Mahmoud KA, Tkac J (2017). Electrochemical performance of Ti3C2Tx MXene in aqueous media: towards ultrasensitive H2O2 sensing. Electrochim Acta.

[171] Michałowski PP, Anayee M, Mathis TS, Kozdra S, Wójcik A, Hantanasirisakul K, Jóźwik I, Piątkowska A, Możdżonek M, Malinowska A, Diduszko R, Wierzbicka E, Gogotsi Y (2022). Oxycarbide MXenes and MAX phases identification using monoatomic layer-by-layer analysis with ultralow-energy secondary-ion mass spectrometry. Nat Nanotechnol.

[172] Alhabeb M, Maleski K, Mathis TS, Sarycheva A, Hatter CB, Uzun S, Levitt A, Gogotsi Y (2018). Selective etching of silicon from Ti3SiC2 (MAX) to obtain 2D titanium carbide (MXene). Angew Chem.

[173] Lorencova L, Bertok T, Filip J, Jerigova M, Velic D, Kasak P, Mahmoud KA, Tkac J (2018). Highly stable Ti3C2Tx (MXene)/Pt nanoparticles-modified glassy carbon electrode for H2O2 and small molecules sensing applications. Sens Actuators, B Chem.

[174] Lorencova L, Gajdosova V, Hroncekova S, Bertok T, Blahutova J, Vikartovska A, Parrakova L, Gemeiner P, Kasak P, Tkac J (2019). 2D MXenes as perspective immobilization platforms for design of electrochemical nanobiosensors. Electroanalysis.

[175] Aguedo J, Lorencova L, Barath M, Farkas P, Tkac J (2020). Electrochemical impedance spectroscopy on 2D nanomaterial MXene modified interfaces: application as a characterization and transducing tool. Chemosensors.

[176] Sarycheva A, Makaryan T, Maleski K, Satheeshkumar E, Melikyan A, Minassian H, Yoshimura M, Gogotsi Y (2017). Two-dimensional titanium carbide (MXene) as surface-enhanced raman scattering substrate. J Phys Chem C.

[177] Lorencova L, Gajdosova V, Hroncekova S, Bertok T, Jerigova M, Velic D, Sobolciak P, Krupa I, Kasak P, Tkac J (2020) Electrochemical investigation of interfacial properties of Ti3C2Tx MXene modified by aryldiazonium betaine derivatives. Frontiers in Chemistry 8:553 10.3389/fchem.2020.00553 10.3389/fchem.2020.00553 PMC7393994 32793549

[178] Do HH, Cho JH, Han SM, Ahn SH, Kim SY (2021). Metal–organic-framework- and MXene-based taste sensors and glucose detection. Sensors.

[179] Kumar R, Singh L (2022) Ti3C2Tx MXene as electrocatalyst for designing robust glucose biosensors. Advanced Materials Technologies 7(12):2200151. 10.1002/admt.202200151

[180] Wang S, Zheng M, Zhang X, Zhuo M, Zhou Q, Su Y, Zheng M, Yuan G, Wang Z (2021). Flowerlike CuO/Au nanoparticle heterostructures for nonenzymatic glucose detection. ACS Appl Nano Mater.

[181] Hu T, Zhang M, Dong H, Li T, Zang X-b, Li X, Ni Z-h (2022). Free-standing MXene/chitosan/Cu2O electrode: an enzyme-free and efficient biosensor for simultaneous determination of glucose and cholesterol. J Zhejiang Uni-Sci A.

[182] Alanazi N, Selvi Gopal T, Muthuramamoorthy M, Alobaidi AAE, Alsaigh RA, Aldosary MH, Pandiaraj S, Almutairi M, Grace AN, Alodhayb A (2023). Cu2O/MXene/rGO ternary nanocomposites as sensing electrodes for nonenzymatic glucose sensors. ACS Appl Nano Mater.

[183] Rakhi RB, Nayak P, Xia C, Alshareef HN (2016). Novel amperometric glucose biosensor based on MXene nanocomposite. Sci Rep.

[184] Gu H, Xing Y, Xiong P, Tang H, Li C, Chen S, Zeng R, Han K, Shi G (2019). Three-dimensional porous Ti3C2Tx MXene-graphene hybrid films for glucose biosensing. ACS App Nano Mat.

[185] Murugan P, Annamalai J, Atchudan R, Govindasamy M, Nallaswamy D, Ganapathy D, Reshetilov A, Sundramoorthy AK (2022). Electrochemical sensing of glucose using glucose oxidase/PEDOT:4-Sulfocalix [4]arene/MXene composite modified electrode. Micromachines.

[186] Gao R, Yang X, Yang Q, Wu Y, Wang F, Xia Q, Bao S-J (2021). Design of an amperometric glucose oxidase biosensor with added protective and adhesion layers. Microchim Acta.

[187] Lei Y, Alshareef AH, Zhao W, Inal S (2020). Laser-scribed graphene electrodes derived from lignin for biochemical sensing. ACS Applied Nano Materials.

[188] Lei Y, Zhao W, Zhang Y, Jiang Q, He J-H, Baeumner AJ, Wolfbeis OS, Wang ZL, Salama KN, Alshareef HN (2019). A MXene-based wearable biosensor system for high-performance in vitro perspiration analysis. Small.

[189] Li Q-F, Chen X, Wang H, Liu M, Peng H-L (2023). Pt/MXene-based flexible wearable non-enzymatic electrochemical sensor for continuous glucose detection in sweat. ACS Appl Mater Interf.

[190] Liu J, Jiang X, Zhang R, Zhang Y, Wu L, Lu W, Li J, Li Y, Zhang H (2019). MXene-enabled electrochemical microfluidic biosensor: applications toward multicomponent continuous monitoring in whole blood. Adv Func Mater.

[191] Zhang M, Liu H, Wang X (2023). Cholesterol oxidase-immobilized MXene/sodium alginate/silica@n-docosane hierarchical microcapsules for ultrasensitive electrochemical biosensing detection of cholesterol. J Mater Chem B.

[192] Xu W, Sakran M, Fei J, Li X, Weng C, Yang W, Zhu G, Zhu W, Zhou X (2021). Electrochemical biosensor based on HRP/Ti3C2/Nafion film for determination of hydrogen peroxide in serum samples of patients with acute myocardial infarction. ACS Biomater Sci Eng.

[193] Deshmukh K, Kovářík T, Khadheer Pasha SK (2020). State of the art recent progress in two dimensional MXenes based gas sensors and biosensors: a comprehensive review. Coord Chem Rev.

[194] Tran VA, Tran NT, Doan VD, Nguyen T-Q, Pham Thi HH, Vo GNL (2023). Application prospects of MXenes materials modifications for sensors. Micromachines.

[195] Zhang Y, Jiang X, Zhang J, Zhang H, Li Y (2019). Simultaneous voltammetric determination of acetaminophen and isoniazid using MXene modified screen-printed electrode. Biosens Bioelectron.

[196] Chen D, Shao S, Zhang W, Zhao J, Lian M (2022). Nitrogen and sulfur co-doping strategy to trigger the peroxidase-like and electrochemical activity of Ti3C2 nanosheets for sensitive uric acid detection. Anal Chim Acta.

[197] Tian L, Zhang J, Fan H, Zhang Y, Wang Z, Oderinde O, Wang Y, Cui J (2023). High efficient electrochemical biosensor based on exonuclease-III-assisted dual-recycling amplification for ultrasensitive detection of kanamycin. Anal Biochem.

[198] Siva Sangu S, Chandra Bose Gopinath S, Abdul Shukur MF, Mohamed Saheed MS (2022). An electrochemical approach for ultrasensitive detection of zearalenone in commodity using disposable screen-printed electrode coated with MXene/chitosan film. BioNanoScience.

[199] Koyappayil A, Chavan SG, Mohammadniaei M, Go A, Hwang SY, Lee M-H (2020). β-Hydroxybutyrate dehydrogenase decorated MXene nanosheets for the amperometric determination of β-hydroxybutyrate. Microchim Acta.

[200] Elumalai S, Mani V, Jeromiyas N, Ponnusamy VK, Yoshimura M (2019). A composite film prepared from titanium carbide Ti3C2Tx (MXene) and gold nanoparticles for voltammetric determination of uric acid and folic acid. Microchim Acta.

[201] Liu X, Qiu Y, Jiang D, Li F, Gan Y, Zhu Y, Pan Y, Wan H, Wang P (2022). Covalently grafting first-generation PAMAM dendrimers onto MXenes with self-adsorbed AuNPs for use as a functional nanoplatform for highly sensitive electrochemical biosensing of cTnT. Microsyst Nanoeng.

[202] Liang H, Chen C, Zeng J, Zhou M, Wang L, Ning G, Duan Q, Han R, Liu H, Zhao H, Li C-P (2022). Dual-signal electrochemical biosensor for neutrophil gelatinase-associated lipocalin based on MXene-polyaniline and Cu-MOF/single-walled carbon nanohorn nanostructures. ACS Appl Nano Mater.

[203] Wang L, Zhang H, Zhuang T, Liu J, Sojic N, Wang Z (2022). Sensitive electrochemiluminescence biosensing of polynucleotide kinase using the versatility of two-dimensional Ti3C2TX MXene nanomaterials. Anal Chim Acta.

[204] Sun X, Sun J, Ye Y, Ji J, Sheng L, Yang D, Sun X (2023). Metabolic pathway-based self-assembled Au@MXene liver microsome electrochemical biosensor for rapid screening of aflatoxin B1. Bioelectrochem.

[205] Yang H, Hou Q, Ding C (2022). Denatured bovine serum albumin hydrogel–based electrochemical biosensors for detection of IgG. Microchim Acta.

[206] Yang J, Xu C, Yang Q, Wei W, Wang C (2022). Ag nanoparticle in situ decorated on Ti3C2Tx with excellent SERS and EIS immunoassay performance for beta-human chorionic gonadotropin. Microchim Acta.

[207] Sung H, Ferlay J, Siegel RL, Laversanne M, Soerjomataram I, Jemal A, Bray F (2021) Global Cancer Statistics 2020: GLOBOCAN Estimates of Incidence and Mortality Worldwide for 36 Cancers in 185 Countries. CA: A Cancer Journal for Clinicians 71(3):209–249. 10.3322/caac.21660 10.3322/caac.21660 33538338

[208] Mohammadniaei M, Nguyen HV, Tieu MV, Lee M-H (2019). 2D materials in development of electrochemical point-of-care cancer screening devices. Micromachines.

[209] Parihar A, Singhal A, Kumar N, Khan R, Khan MA, Srivastava AK (2022). Next-generation intelligent MXene-based electrochemical aptasensors for point-of-care cancer diagnostics. Nano-Micro Letters.

[210] Aguedo J, Pakanova Z, Lorencova L, Nemcovic M, Kasak P, Barath M, Farkas P, Tkac J (2022). MXene as a novel cartridge for N-glycan enrichment. Anal Chim Acta.

[211] Hroncekova S, Bertók T, Hires M, Jane E, Lorencova L, Vikartovská A, Tanvir A, Kasák P, Tkac J (2020). Ultrasensitive Ti3C2TX MXene/chitosan nanocomposite-based amperometric biosensor for detection of potential prostate cancer marker in urine samples. Processes.

[212] Bertok T, Bertokova A, Hroncekova S, Chocholova E, Svecova N, Lorencova L, Kasak P, Tkac J (2021) Novel prostate cancer biomarkers: aetiology, clinical performance and sensing applications. CHEMOSENSORS 9(8):205. 10.3390/chemosensors9080205

[213] Hroncekova S, Lorencova L, Bertok T, Hires M, Jane E, Bučko M, Kasak P, Tkac J (2023) Amperometric miniaturised portable enzymatic nanobiosensor for the ultrasensitive analysis of a prostate cancer biomarker. J Funct Biomater 14(3):161. 10.3390/jfb14030161 10.3390/jfb14030161 PMC10056543 36976085

[214] Ran B, Chen C, Liu B, Lan M, Chen H, Zhu Y (2022). A Ti3C2TX/Pt–Pd based amperometric biosensor for sensitive cancer biomarker detection. Electrophoresis.

[215] Divya KP, Keerthana S, Viswanathan C, Ponpandian N (2023). MXene supported biomimetic bilayer lipid membrane biosensor for zeptomole detection of BRCA1 gene. Microchim Acta.

[216] Zhao J, He C, Wu W, Yang H, Dong J, Wen L, Hu Z, Yang M, Hou C, Huo D (2022). MXene-MoS2 heterostructure collaborated with catalyzed hairpin assembly for label-free electrochemical detection of microRNA-21. Talanta.

[217] Ranjbari S, Rezayi M, Arefinia R, Aghaee-Bakhtiari SH, Hatamluyi B, Pasdar A (2023). A novel electrochemical biosensor based on signal amplification of Au HFGNs/PnBA-MXene nanocomposite for the detection of miRNA-122 as a biomarker of breast cancer. Talanta.

[218] Du JF, Chen JS, Liu XP, Mao CJ, Jin BK (2022). Coupled electrochemiluminescent and resonance energy transfer determination of microRNA-141 using functionalized Mxene composite. Mikrochim Acta.

[219] Mohammadniaei M, Koyappayil A, Sun Y, Min J, Lee M-H (2020). Gold nanoparticle/MXene for multiple and sensitive detection of oncomiRs based on synergetic signal amplification. Biosens Bioelectron.

[220] Meng Y, Qin N, Hun X (2021). ZnSe nanodisks:Ti3C2 MXenes-modified electrode for nucleic acid liquid biopsy with photoelectrochemical strategy. Microchim Acta.

[221] Gajdosova VP, Lorencova L, Kasak P, Jerigova M, Velic D, Orovcik L, Barath M, Farkas P, Tkac J (2022). Redox features of hexaammineruthenium(III) on MXene modified interface: three options for affinity biosensing. Anal Chim Acta.

[222] Gajdosova V, Lorencova L, Kasak P, Tkac J (2020). Electrochemical nanobiosensors for detection of breast cancer biomarkers. Sensors.

[223] Soomro RA, Jawaid S, Zhang P, Han X, Hallam KR, Karakuş S, Kilislioğlu A, Xu B, Willander M (2021). NiWO4-induced partial oxidation of MXene for photo-electrochemical detection of prostate-specific antigen. Sens Actuators, B Chem.

[224] Xu Y, Wang X, Ding C, Luo X (2021). Ratiometric antifouling electrochemical biosensors based on multifunctional peptides and MXene loaded with Au nanoparticles and methylene blue. ACS Appl Mater Interf.

[225] Song X, Gao H, Yuan R, Xiang Y (2022). Trimetallic nanoparticle-decorated MXene nanosheets for catalytic electrochemical detection of carcinoembryonic antigen via Exo III-aided dual recycling amplifications. Sens Actuators, B Chem.

[226] Zhou S, Gu C, Li Z, Yang L, He L, Wang M, Huang X, Zhou N, Zhang Z (2019). Ti3C2Tx MXene and polyoxometalate nanohybrid embedded with polypyrrole: ultra-sensitive platform for the detection of osteopontin. Appl Surf Sci.

[227] Kumar S, Lei Y, Alshareef NH, Quevedo-Lopez MA, Salama KN (2018). Biofunctionalized two-dimensional Ti3C2 MXenes for ultrasensitive detection of cancer biomarker. Biosens Bioelectron.

[228] Xu P, Lu C, Wang D, Fu D (2021). Combination of ultrathin micro-patterned MXene and PEDOT: poly(styrenesulfonate) enables organic electrochemical transistor for amperometric determination of survivin protein in children osteosarcoma. Microchim Acta.

[229] Kalkal A, Tiwari A, Sharma D, Baghel MK, Kumar P, Pradhan R, Packirisamy G (2023). Air-brush spray coated Ti3C2-MXene-graphene nanohybrid thin film based electrochemical biosensor for cancer biomarker detection. Int J Biol Macromol.

[230] Zhang H, Wang Z, Wang F, Zhang Y, Wang H, Liu Y (2021). Ti3C2 MXene mediated Prussian blue in situ hybridization and electrochemical signal amplification for the detection of exosomes. Talanta.

[231] Li X, Lu Y, Liu Q (2021). Electrochemical and optical biosensors based on multifunctional MXene nanoplatforms: progress and prospects. Talanta.

[232] Duan H, Wang Y, Tang S-Y, Xiao T-H, Goda K, Li M (2023). A CRISPR-Cas12a powered electrochemical sensor based on gold nanoparticles and MXene composite for enhanced nucleic acid detection. Sens Actuat B: Chem.

[233] Wang Y, Sun W, Li Y, Zhuang X, Tian C, Luan F, Fu X (2021). Imidazole metal-organic frameworks embedded in layered Ti3C2Tx Mxene as a high-performance electrochemiluminescence biosensor for sensitive detection of HIV-1 protein. Microchem J.

[234] Liu Y, Huang S, Li J, Wang M, Wang C, Hu B, Zhou N, Zhang Z (2021). 0D/2D heteronanostructure–integrated bimetallic CoCu-ZIF nanosheets and MXene-derived carbon dots for impedimetric cytosensing of melanoma B16–F10 cells. Microchim Acta.

[235] Lian M, Shi Y, Chen L, Qin Y, Zhang W, Zhao J, Chen D (2022). Cell membrane and V2C MXene-based electrochemical immunosensor with enhanced antifouling capability for detection of CD44. ACS Sens.

[236] Kedambaimoole V, Harsh K, Rajanna K, Sen P, Nayak MM, Kumar S (2022). MXene wearables: properties, fabrication strategies, sensing mechanism and applications. Materials Advances.

[237] Meng Q, Yang C, Tai X, Cheng K, Li P, Li H, Liu X, Liu S (2022). Recent advances in MXenes and their composites for wearable sensors. J Phys: Condens Matter.

[238] Li Y, Huang S, Peng S, Jia H, Pang J, Ibarlucea B, Hou C, Cao Y, Zhou W, Liu H, Cuniberti G (2023). Toward smart sensing by MXene. Small.

[239] Aziz A, Asif M, Ashraf G, Iftikhar T, Hussain W, Wang S (2022). Environmental significance of wearable sensors based on MXene and graphene. Trends in Environ Anal Chem.

[240] Li T, Chen L, Yang X, Chen X, Zhang Z, Zhao T, Li X, Zhang J (2019). A flexible pressure sensor based on an MXene–textile network structure. J Mater Chem C.

[241] Cai Y, Shen J, Ge G, Zhang Y, Jin W, Huang W, Shao J, Yang J, Dong X (2018). Stretchable Ti3C2Tx MXene/carbon nanotube composite based strain sensor with ultrahigh sensitivity and tunable sensing range. ACS Nano.

[242] Raza T, Qu L, Khokhar WA, Andrews B, Ali A, Tian M (2021). Progress of wearable and flexible electrochemical biosensors with the aid of conductive nanomaterials. Front Bioeng Biotechnol.

[243] Zheng J, Wang B, Ding A, Weng B, Chen J (2018). Synthesis of MXene/DNA/Pd/Pt nanocomposite for sensitive detection of dopamine. J Electroanal Chem.

[244] Guo Y, Zhong M, Fang Z, Wan P, Yu G (2019). A wearable transient pressure sensor made with MXene nanosheets for sensitive broad-range human–machine interfacing. Nano Lett.

[245] Gao Y, Yan C, Huang H, Yang T, Tian G, Xiong D, Chen N, Chu X, Zhong S, Deng W, Fang Y, Yang W (2020). Microchannel-confined MXene based flexible piezoresistive multifunctional micro-force sensor. Adv Funct Mater.

[246] Li X-P, Li Y, Li X, Song D, Min P, Hu C, Zhang H-B, Koratkar N, Yu Z-Z (2019). Highly sensitive, reliable and flexible piezoresistive pressure sensors featuring polyurethane sponge coated with MXene sheets. J Colloid Interface Sci.

[247] Yue Y, Liu N, Liu W, Li M, Ma Y, Luo C, Wang S, Rao J, Hu X, Su J, Zhang Z, Huang Q, Gao Y (2018). 3D hybrid porous Mxene-sponge network and its application in piezoresistive sensor. Nano Energy.

[248] Cheng Y, Ma Y, Li L, Zhu M, Yue Y, Liu W, Wang L, Jia S, Li C, Qi T, Wang J, Gao Y (2020). Bioinspired microspines for a high-performance spray Ti3C2Tx MXene-based piezoresistive sensor. ACS Nano.

[249] Zhuo H, Hu Y, Chen Z, Peng X, Liu L, Luo Q, Yi J, Liu C, Zhong L (2019). A carbon aerogel with super mechanical and sensing performances for wearable piezoresistive sensors. J Mater Chem A.

[250] Yang Y, Shi L, Cao Z, Wang R, Sun J (2019). Strain sensors with a high sensitivity and a wide sensing range based on a Ti3C2Tx (MXene) nanoparticle–nanosheet hybrid network. Adv Funct Mater.

[251] Fan Y, Kong F, Yang J, Xiong X, Gao S, Yuan J, Meng S, Chen L (2023). Flexible wearable sensor based on SF/EEP/GR/MXene nanocomposites. Appl Phys A.

[252] Gong T, Zn Li, Liang H, Li Y, Tang X, Chen F, Hu Q, Wang H (2023). High-sensitivity wearable sensor based on a MXene nanochannel self-adhesive hydrogel. ACS Appl Mater Interf.

[253] Yang H, Li J, Xiao X, Wang J, Li Y, Li K, Li Z, Yang H, Wang Q, Yang J (2022). Topographic design in wearable MXene sensors with in-sensor machine learning for full-body avatar reconstruction. Nat Commun.

[254] Yang Y-C, Lin Y-T, Yu J, Chang H-T, Lu T-Y, Huang T-Y, Preet A, Hsu Y-J, Wang L, Lin T-E (2021). MXene nanosheet-based microneedles for monitoring muscle contraction and electrostimulation treatment. ACS Applied Nano Materials.

[255] Yi Q, Pei X, Das P, Qin H, Lee SW, Esfandyarpour R (2022). A self-powered triboelectric MXene-based 3D-printed wearable physiological biosignal sensing system for on-demand, wireless, and real-time health monitoring. Nano Energy.

[256] Shi Z, Dai C, Deng P, Li X, Wu Y, Lv J, Xiong C, Shuai Y, Zhang F, Wang D, Liang H, He Y, Chen Q, Lu Y, Liu Q (2023). Wearable battery-free smart bandage with peptide functionalized biosensors based on MXene for bacterial wound infection detection. Sens Actuators, B Chem.

[257] Zhang W, Ma C, Huang L-Z, Guo W-Y, Li D-D, Bian J, Ma M-G (2021). Stretchable, antifreezing, non-drying, and fast-response sensors based on cellulose nanocomposite hydrogels for signal detection. Macromol Mater Eng.

[258] Ganesan S, Ramajayam K, Kokulnathan T, Palaniappan A (2023). Recent advances in two-dimensional MXene-based electrochemical biosensors for sweat analysis. Molecules.

[259] Wu M, Zhang Q, Fang Y, Deng C, Zhou F, Zhang Y, Wang X, Tang Y, Wang Y (2021). Polylysine-modified MXene nanosheets with highly loaded glucose oxidase as cascade nanoreactor for glucose decomposition and electrochemical sensing. J Colloid Interface Sci.

[260] Hroncekova S, Lorencova L, Bertok T, Hires M, Jane E, Bučko M, Kasak P, Tkac J (2023). Amperometric miniaturised portable enzymatic nanobiosensor for the ultrasensitive analysis of a prostate cancer biomarker. Journal of Functional Biomaterials.

[261] Ding R, Jiang W, Ma Y, Yang Q, Han X, Hou X (2023). A highly sensitive MXene/AuPt/AChE-based electrochemical platform for the detection of chlorpyrifos. Microchem J.

[262] Li Z, Guo Y, Yue H, Gao X, Huang S, Zhang X, Yu Y, Zhang H, Zhang H (2021). Electrochemical determination of epinephrine based on Ti3C2Tx MXene-reduced graphene oxide/ITO electrode. J Electroanal Chem.

[263] Shahzad F, Iqbal A, Zaidi SA, Hwang S-W, Koo CM (2019). Nafion-stabilized two-dimensional transition metal carbide (Ti3C2Tx MXene) as a high-performance electrochemical sensor for neurotransmitter. J Ind Eng Chem.

[264] Liu G, Xia T, Liang X, Hou S, Hou S (2022). Enzymatic electrochemical biosensor from Eu-doped SnO2 embedded in MXene for high performance sensing lactate. ChemElectroChem.

[265] Li Z, Wang Z, Nie Y, Wang P, Ma Q (2022). A novel GSH-capping MXene QD-based ECL biosensor for the detection of miRNA221 in triple-negative breast cancer tumor. Chem Eng J.

[266] Wu Q, Li Z, Liang Q, Ye R, Guo S, Zeng X, Hu J, Li A (2022). Ultrasensitive electrochemical biosensor for microRNA-377 detection based on MXene-Au nanocomposite and G-quadruplex nano-amplification strategy. Electrochim Acta.

[267] Yang X, Feng M, Xia J, Zhang F, Wang Z (2020). An electrochemical biosensor based on AuNPs/Ti3C2 MXene three-dimensional nanocomposite for microRNA-155 detection by exonuclease III-aided cascade target recycling. J Electroanal Chem.

[268] Xu Q, Xu J, Jia H, Tian Q, Liu P, Chen S, Cai Y, Lu X, Duan X, Lu L (2020). Hierarchical Ti3C2 MXene-derived sodium titanate nanoribbons/PEDOT for signal amplified electrochemical immunoassay of prostate specific antigen. J Electroanal Chem.

[269] Liu X, Bai L, Cao X, Wu F, Yin T, Lu W (2022). Rapid determination of SARS-CoV-2 nucleocapsid proteins based on 2D/2D MXene/P–BiOCl/Ru (bpy) 32+ heterojunction composites to enhance electrochemiluminescence performance. Anal Chim Acta.

[270] Ranjbar S, Astani NA, Atabay M, Naseri N, Esfandiar A, Ejtehadi MR (2022). Electrochemical and computational studies of bio-mimicked Ti3C2Tx MXene-based sensor with multivalent interface. J Colloid Interface Sci.

[271] Rizi KS, Hatamluyi B, Darroudi M, Meshkat Z, Aryan E, Soleimanpour S, Rezayi M (2022). PCR-free electrochemical genosensor for Mycobacterium tuberculosis complex detection based on two-dimensional Ti3C2 Mxene-polypyrrole signal amplification. Microchem J.

